# Analysis of Differentiation Protocols Defines a Common Pancreatic Progenitor Molecular Signature and Guides Refinement of Endocrine Differentiation

**DOI:** 10.1016/j.stemcr.2019.11.010

**Published:** 2019-12-26

**Authors:** Agata Wesolowska-Andersen, Rikke Rejnholdt Jensen, Marta Pérez Alcántara, Nicola L. Beer, Claire Duff, Vibe Nylander, Matthew Gosden, Lorna Witty, Rory Bowden, Mark I. McCarthy, Mattias Hansson, Anna L. Gloyn, Christian Honore

**Affiliations:** 1 Wellcome Centre Human Genetics, University of Oxford, OX3 7BN Oxford, UK; 2 Oxford Centre for Diabetes, Endocrinology and Metabolism, University of Oxford, OX3 7LE Oxford, UK; 3 NIHR Oxford Biomedical Research Centre, Churchill Hospital, OX3 7LE Oxford, UK; 4 The MRC Weatherall Institute of Molecular Medicine, University of Oxford, OX3 9DS Oxford, UK; 5 Stem Cell R&D, Novo Nordisk A/S, 2760 Måløv, Denmark

**Keywords:** pluripotent stem cells, directed differentiation, pancreatic progenitors, pancreatic endoderm, endocrine differentiation, multi-omics analysis, transcriptomics, open chromatin, cell identity, disease modeling

## Abstract

Several distinct differentiation protocols for deriving pancreatic progenitors (PPs) from human pluripotent stem cells have been described, but it remains to be shown how similar the PPs are across protocols and how well they resemble their *in vivo* counterparts. Here, we evaluated three differentiation protocols, performed RNA and assay for transposase-accessible chromatin using sequencing on isolated PPs derived with these, and compared them with fetal human pancreas populations. This enabled us to define a shared transcriptional and epigenomic signature of the PPs, including several genes not previously implicated in pancreas development. Furthermore, we identified a significant and previously unappreciated cross-protocol variation of the PPs through multi-omics analysis and demonstrate how such information can be applied to refine differentiation protocols for derivation of insulin-producing beta-like cells. Together, our study highlights the importance of a detailed characterization of defined cell populations derived from distinct differentiation protocols and provides a valuable resource for exploring human pancreatic development.

## Highlights

• Systematic comparison of three protocols for hPSC-pancreatic progenitor differentiation
• Individual hPSC lines differentiate preferentially with different protocols
• Shared transcriptomic and epigenomic signatures of hPSC-pancreatic progenitors
• Molecular characterization of hPSC-pancreatic progenitors guides protocol refinement

## Teaser

Wesolowska-Andersen and colleagues present a comprehensive molecular and functional analysis of human PSC-derived pancreatic progenitors derived using three differentiation protocols. They define their shared transcriptomic and epigenomic signatures but also highlight significant differences between protocols, which are then used to guide protocol modifications to enhance further differentiation toward endocrine lineage.

## Introduction

Human pluripotent stem cells (hPSCs) have tremendous potential for modeling human diseases *in vitro* as well as for regenerative medicine in degenerative diseases. However, the realization of both these applications of hPSCs is dependent on the ability to derive the relevant cell lineages from hPSCs by directed differentiation.

In the context of pancreas development, studies in mice have demonstrated that exocrine, ductal, and endocrine lineages all derive from multipotent pancreatic progenitor (PP) cells, defined by co-expression of several transcription factors (TFs), including PDX1, NKX6.1, PTF1a, and SOX9 (Larsen and Grapin-Botton, 2017). Despite noteworthy differences in human pancreas development compared with mouse (Jennings et al., 2015, Nair and Hebrok, 2015), human PPs express a similar core network of TFs, including PDX1 and NKX6.1 (Petersen et al., 2018). When transplanted into immunocompromised mice, the hPSC-derived PPs are able to give rise to all lineages of the pancreas (Kelly et al., 2011, Kroon et al., 2008, Rezania et al., 2012, Rezania et al., 2013), supporting their similarity to multipotent PPs observed during development. Knowledge gained from rodent models of pancreas development facilitated many of the advancements in differentiation protocols. For example, retinoic acid and fibroblast growth factor signaling are indispensable for the specification and expansion of PPs during development (Bhushan et al., 2001, Molotkov et al., 2005), and the majority of current differentiation protocols include agonists of these signaling pathways. However, there are also notable differences in protocols reported to differentiate hPSCs to PPs. For example, bone morphogenetic protein (BMP) signaling has been shown to promote a liver fate choice rather than pancreas development (Wandzioch and Zaret, 2009), and thus several protocols include BMP inhibitors during differentiation. However, a recent report argued for the exclusion of BMP inhibitors, since these were shown to promote a premature endocrine differentiation at the expense of PDX1/NKX6.1-positive PPs (Russ et al., 2015). There is also no consensus on inclusion of other pathway modulators, such as epidermal growth factor (EGF) or protein kinase C (PKC) agonists, in the differentiation protocols (Nostro et al., 2015, Rezania et al., 2014, Russ et al., 2015).

As hPSC-derived PPs are often defined by co-expression of a limited set of genes (e.g., *PDX1* and *NXK6.1*), it remains unclear how similar the PP cells derived from various differentiation protocols are and how well they represent embryonic development and subsequent development of more mature cell types of the pancreatic islet. To address these questions, we performed a detailed characterization of PPs derived using three differentiation protocols adapted from recent publications (Nostro et al., 2015, Rezania et al., 2014, Russ et al., 2015), although with several differences compared with the original publications. We were able to recapitulate several aspects of the reported protocols and achieved efficient differentiation toward PPs across all three protocols. Isolation of PPs allowed us to define their comprehensive gene expression and chromatin accessibility signatures shared across all protocols, which shed light on new endocrine pancreas development biology and will serve as a valuable resource for future studies in disease modeling and cellular replacement therapies. We also highlight several notable differences in the omics profiles of the PPs derived with the different protocols, which translate to differences in their ability to differentiate further toward the endocrine lineage. Finally, we illustrate the utility of these datasets to further optimize various stages of the differentiation protocols to improve the endocrine differentiation and the derivation of beta-like cells from hPSCs.

## Results

### Efficient Derivation of PPs from Multiple hPSC Lines Using Three Distinct Differentiation Protocols

**Figure 1:**
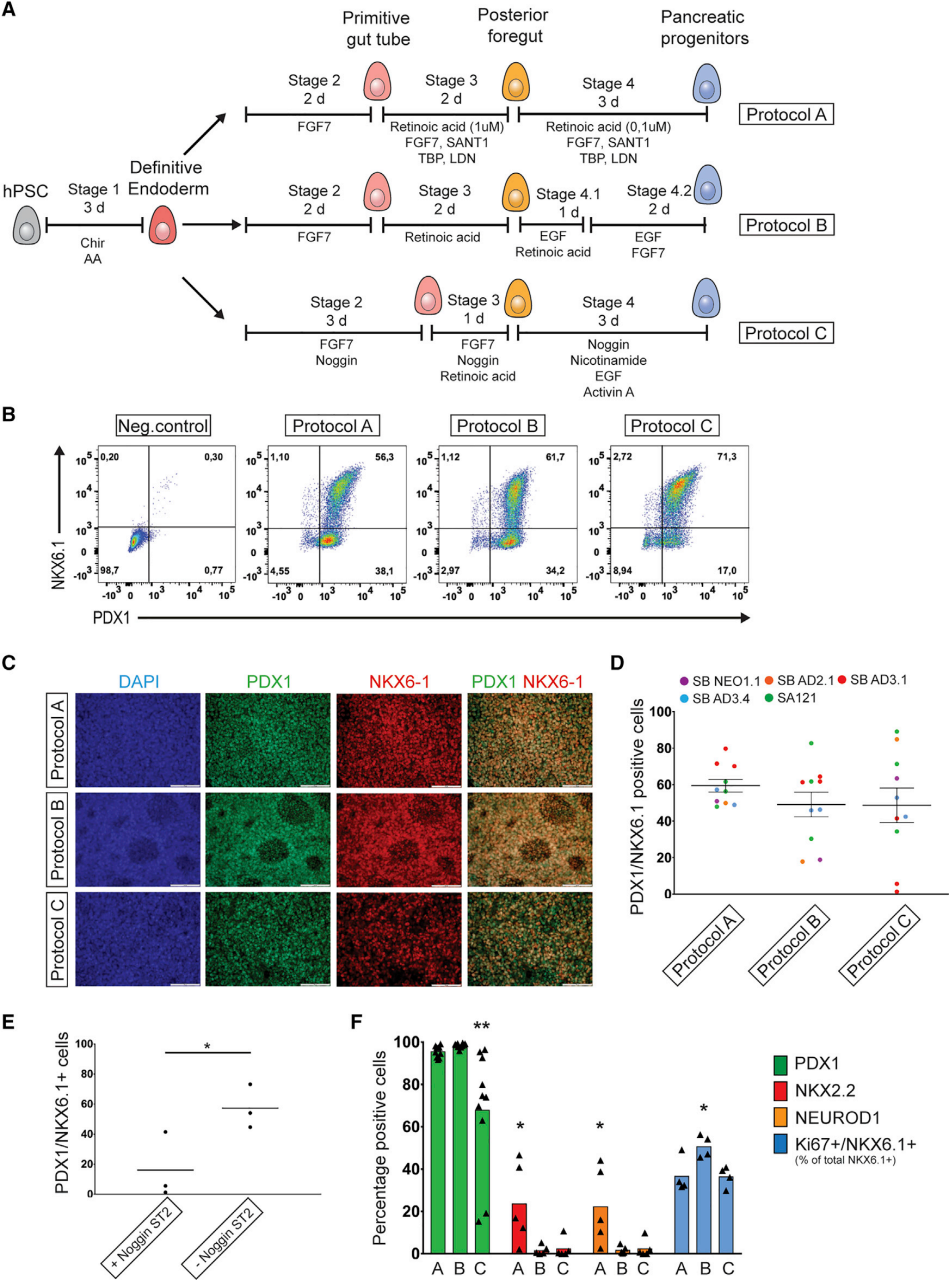
Derivation of PPs from hPSCs Using Multiple Differentiation Protocols (A) Schematic outline of the three PP protocols applied. (B) Representative examples of flow cytometry pseudo color dot plots of PPs from the three protocols stained for PDX1 and NKX6.1. Negative control is definitive endoderm cells. (C) Immunofluorescence images of PPs stained for PDX1 and NKX6.1. Scale bar, 100 μm. (D) Quantification of PDX1 and NKX6.1 co-expressing cells based on the flow cytometric analysis shown in (B). Graph shows a scatterplot of the mean ± SEM of five individual hPSC lines. Dots are color coded according to individual cell lines (details in Figure S1C). n = 10 independent experiments. (E) Percentage of PDX1 and NKX6.1 co-expressing cells from the SB AD3.1 hiPSC line differentiated with protocol C with or without 50 ng/mL Noggin included during stage two; n = 3 independent experiments; ^∗^p < 0.05, paired t test. (F) Quantification of PDX1, NKX2.2, NEUROD1, and percentage NKX6.1+ cells co-expressing Ki67. Bars show means and dots represent individual differentiations. One-way ANOVA with the Tukey test for multiple comparisons, ^∗^p < 0.05, ^∗∗^p < 0.01, different from the two other groups. PDX1, n = 10 independent experiments, same hPSC lines as in (D). NKX2.2 and NEUROD1, n = 5 independent experiments, one for each of the following hPSC lines: SA121 hESC, SB NEO1.1 hiPSC, SB AD2.1 hiPSC, SB AD3.1 hiPSC, SB AD3.4 hiPSC. Ki67, n = 4 independent experiments, three using SB AD3.1 hiPSC and one using SA121 hESC.

To compare PP differentiation protocols, we first applied a common protocol for efficient derivation of definitive endoderm (DE) from hPSCs (Rezania et al., 2014, Perez-Alcantara et al., 2018) (Figures S1A and S1B). DE was differentiated toward PPs using protocols outlined in Figure 1A. The three differentiation protocols (A, B, and C) were adapted from previously established protocols (Nostro et al., 2015, Rezania et al., 2014, Russ et al., 2015), but with several differences compared with the original publications (protocol details outlined in Supplemental Experimental Procedures, including key differences from the published protocols). Differentiation efficiency was assessed by flow cytometer and immunofluorescence imaging analysis of expression of the two key PP TFs: PDX1 and NKX6.1 (Figures 1B–1D). Across five hPSC lines we observed an average differentiation efficiency of 59%, 49%, and 48% PDX1/NKX6.1-positive cells derived using protocols A, B, and C, respectively (Figure 1D). A notable variation in the efficiency of PDX1/NKX6.1-positive cells was observed across differentiations (Figure 1D), which may be attributed to the different abilities of individual hPSC lines to differentiate to PPs across these protocols. The hPSC line SB AD3.1 displayed poor differentiation efficiency using protocol C (Figures 1D and S1C), but omission of Noggin during stage 2 of protocol C led to a significant improvement of its differentiation (Figure 1E). Extending this observation, we evaluated the effect of Noggin during stage 2 of all three protocols on the PP differentiation efficiency; however, we found that the effect was not consistent across either cell lines or differentiation protocols (Figure S1C).

A large proportion of the PDX1/NKX6.1-positive cells across the three protocols were proliferating, as demonstrated by the expression of the proliferation marker Ki67 (Figure 1F). Protocols B and C were originally reported to generate a high percentage of PPs, while limiting the commitment to the endocrine lineage (Russ et al., 2015, Nostro et al., 2015). In agreement with this, we observed very few cells expressing the endocrine markers NEUROD1 and NKX2.2 in protocol B and C, whereas protocol A gave rise to a significantly higher number of endocrine cells at this stage of the protocol (Figure 1F). Most of the endocrine progenitors generated were NKX6.1 negative (Figures S1D and S1E), in agreement with previous studies (Nostro et al., 2015, Petersen et al., 2017, Russ et al., 2015). In summary, we are able to recapitulate key aspects of the original reports and we show that all three differentiation protocols efficiently derive PDX1/NKX6.1-positive PPs across multiple hPSC lines.

### Global Gene Expression and Chromatin Accessibility Analysis of hPSC-Derived PPs

**Figure 2:**
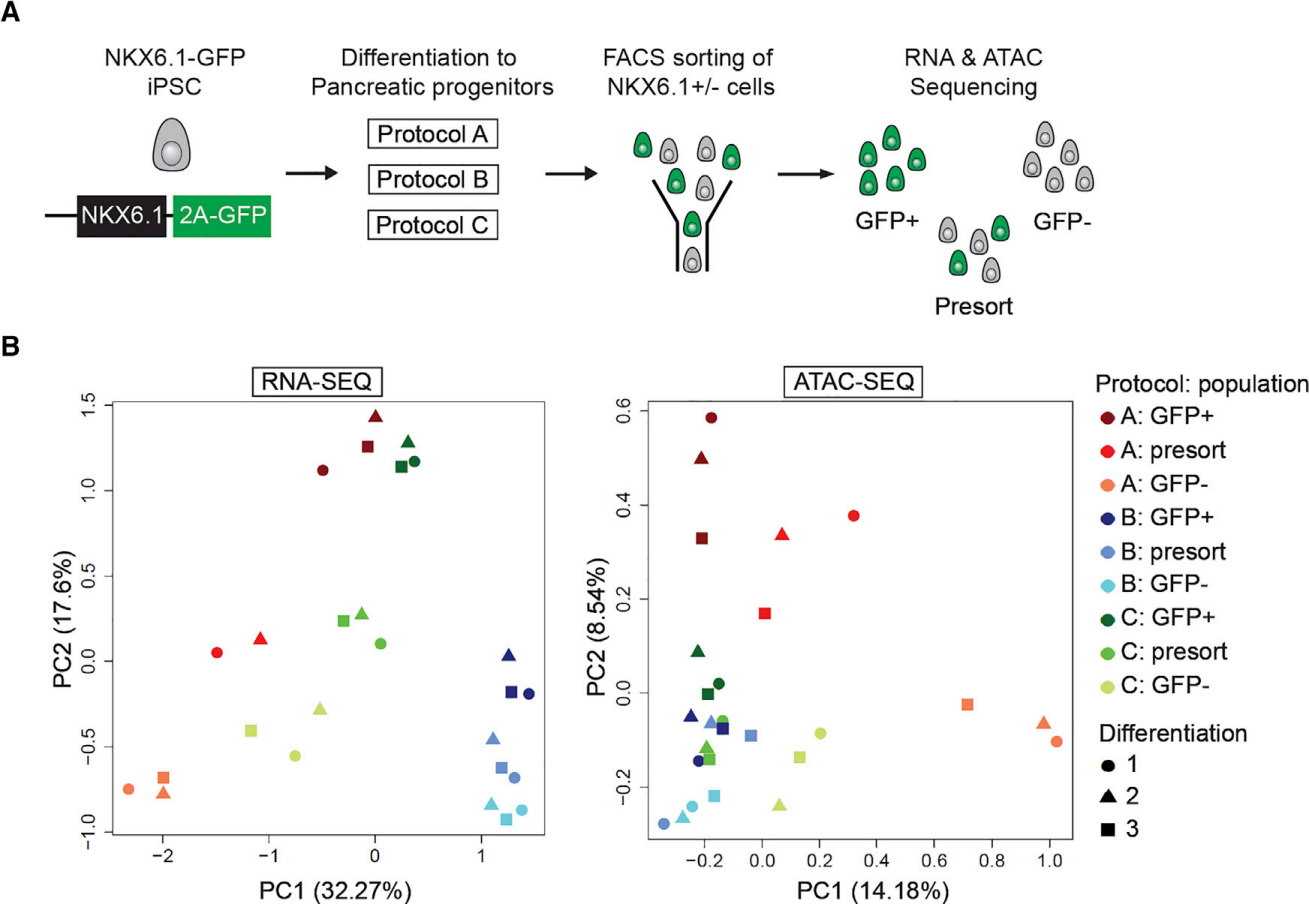
Global Gene Expression and Chromatin Accessibility Analysis of FACS-Isolated PP Populations (A) Schematic showing the experimental setup. NKX6.1-GFP hiPSCs were differentiated side by side using all three protocols and GFP+ and GFP− cells as well as unsorted cells were collected following FACS for RNA and ATAC sequencing. Cells were collected from three independent differentiations of all three protocols. (B) Principal component analysis (PCA) of RNA-seq (left) and ATAC-seq data (right). Legend applies to both PCA plots.

We next aimed to characterize the PPs derived from the three differentiation protocols in more detail. We differentiated an NKX6.1-GFP reporter human induced PSC (hiPSC) line (Gupta et al., 2018) to PPs using the three differentiation protocols and isolated both GFP+ and GFP− cells using fluorescence-activated cell sorting (FACS) (Figure 2A). Post-sort analysis of live cells demonstrated isolation of highly pure GFP+ and GFP− populations across all three protocols (Figures S2A and S2B) and efficient differentiation of the reporter line to PPs by all protocols was confirmed by flow cytometry analysis of GFP and NKX6.1 expression (Figures S2C and S2D). The isolated populations were processed for genome-wide transcriptome and chromatin accessibility analysis by RNA sequencing (RNA-seq) and assay for transposase-accessible chromatin (ATAC) using sequencing (ATAC-seq), respectively. Global principal component analysis (PCA) of the datasets (Figure 2B) revealed that samples clustered by protocol and by sorted cell population. Samples generated with protocol A were the most distinct and showed the largest separation of the GFP-sorted cell populations in both datasets. For all three protocols, we observed the presort samples clustering in between the sorted populations, as expected. In addition, we confirmed that expression of *NKX6.1* and *PDX1* was enriched in the GFP+ population for all protocols (Figures S2E and S2F).

### Cross-Protocol PP Gene Expression and Chromatin Accessibility Signature

**Figure 3:**
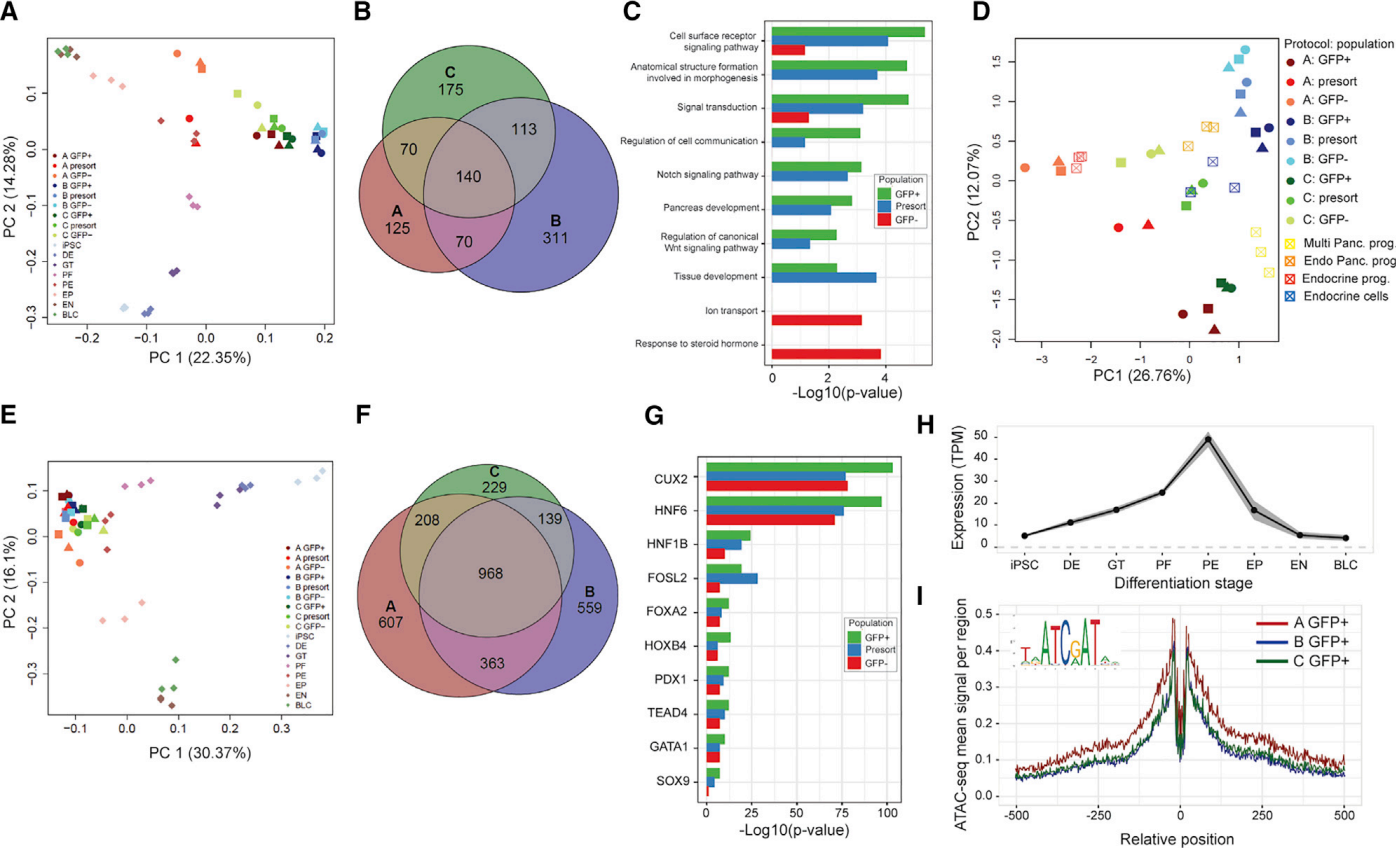
Common Transcriptomic and Epigenomic PP Signatures across Three Differentiation Protocols (A) PCA of RNA-seq samples together with data collected at all stages of the hPSC differentiation toward beta-like cells generated with protocol A (Perez-Alcantara et al., 2018). (B) Venn diagram of the RNA-seq GFP+ PP signatures generated separately for each of the protocols by comparison with cells at the other differentiation stages. (C) Selected gene ontology enrichment of the common PP signature genes across the three protocols; shown separately for signatures derived with presort cells (in blue), GFP+ (in green), and GFP− (in red) cell populations. The length of the bar represents −log10 of the enrichment p value. (D) PCA of RNA-seq samples from this study, together with transcriptomes of fetal pancreas cell subpopulations (Ramond et al., 2018). (E) PCA of ATAC-seq samples from this study, together with data collected at all stages of the hPSC differentiation toward beta-like cells, generated with protocol A (Perez-Alcantara et al., 2018). (F) Venn diagram of the ATAC-seq GFP+ PP signatures generated separately for each of the protocols by comparison with cells at the other differentiation stages. (G) Enrichment of selected TFs within the common PP signature open chromatin peaks across the three protocols; shown separately for signatures derived with presort cells (in blue), GFP+ (in green), and GFP− (in red) cell populations. (H) Mean expression of CUX2 gene across all stages of hPSC differentiation toward beta-like cells. Shaded gray area indicates ± SEM (Perez-Alcantara et al., 2018). (I) Footprinting analysis of CUX2 binding motifs within open chromatin peaks of the GFP+ populations generated with the three differentiation protocols. BLC, beta-like cells; EN, endocrine cells; ; EP, endocrine progenitors; GT, gut tube; PE, pancreatic endoderm/progenitors; PF, posterior foregut.

We then explored the similarities of the omics profiles of the PPs derived with the different protocols. For that purpose, we compared the samples generated in this study with gene expression and open chromatin profiles of our previously published differentiation model (Perez-Alcantara et al., 2018) across all seven stages of hPSC differentiation toward beta-like cells. The PCA analysis for both the RNA-seq (Figure 3A) and ATAC-seq (Figure 3E) datasets revealed that all the samples characterized in this study clustered, as expected, together with the pancreatic endoderm stage samples from the control dataset. Interestingly, the GFP− cells generated with protocol A clustered out close to the cells from the subsequent differentiation stage, the endocrine progenitors. This was most pronounced in the RNA-seq data, but also apparent in the ATAC-seq data, and is in line with the higher percentage of endocrine cells observed in the NKX6.1− cells with this protocol (Figures 1F, S1D, S1E, S2C, and S2D).

We then sought to derive a PP gene expression signature common to all three protocols by comparing the transcriptome profiles of the GFP+ cell populations from each of the three protocols separately with the gene expression profiles of the remaining differentiation stages. This resulted in 405, 634, and 498 genes defining the PPs in protocols A, B, and C, respectively, and we next defined the 140-gene common cross-protocol signature by intersecting the three individual protocol-specific signatures (Figure 3B). In a similar manner, we defined a 112-gene PP signature within the presorted cells, and a 129-gene signature within the GFP− cell populations (Table S1). Functional enrichment analysis revealed that several genes forming the signature were involved in cell surface receptor signaling pathways, including Notch and Wnt pathways, and included several genes previously implicated in pancreas development (Figure 3C, Table S1). In addition, for an unbiased comparison with the transcriptomes of PPs *in vivo*, we compared the expression of the samples generated in this study with recently published transcriptomes of human fetal pancreas subpopulations (Ramond et al., 2018) (Figure 3D). We observed that the GFP− population derived with protocol A resembled closely the fetal endocrine progenitor population, in agreement with the high proportion of endocrine differentiation observed with this protocol. Interestingly, the GFP+ populations of protocols A and C clustered close to the fetal multipotent PPs, suggesting a high level of similarity between these *in vitro*-generated PP and human fetal multipotent PP cells. The other two fetal pancreatic cell subpopulations, comprising endocrine-biased PP and endocrine cells, clustered in between the presorted cell populations generated by protocols B and C.

In a similar manner, we derived the open chromatin PP signature, comprising a total of 968 ATAC-seq peaks within the GFP+ population (Figure 3F, Table S1). These open chromatin regions were significantly enriched in binding sites of CUX2 and HNF6 as well as several other TFs with previously established functions in pancreatic development and links to monogenic diabetes, including HNF1B, FOXA2, and PDX1 (Figure 3G). CUX2 was the most significantly enriched TF; however, it has to our knowledge not been implicated in pancreatic development. *CUX2* was expressed in the PP cell populations derived with all three protocols, and its expression was highest at this stage of differentiation in the control lines (Figure 3H). We have subsequently performed a footprinting analysis of the ATAC-seq peaks containing the CUX2 binding motif (Figure 3I) and observed a clear dip in the sequencing coverage around the investigated CUX2 sites, supporting the hypothesis that this TF is bound within the open chromatin at this stage.

### Correlated Modules of Gene Expression and Open Chromatin Highlight Between-Protocol Differences

**Figure 4:**
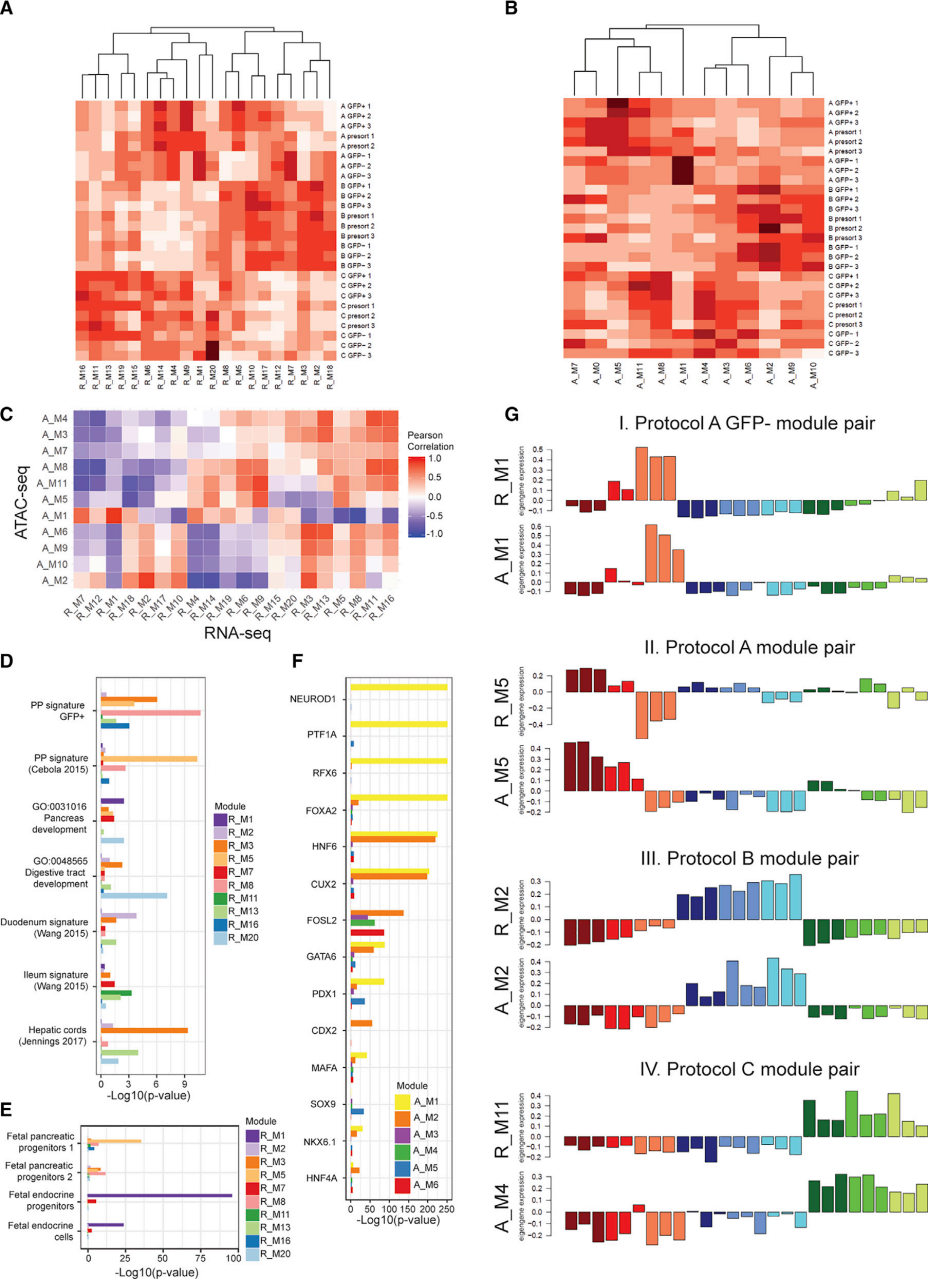
Protocol-Specific Differences in Transcriptomic and Epigenomic Profiles of PPs Generated with Three Differentiation Protocols (A) Heatmap of RNA-seq co-expressed gene modules eigengenes across all RNA-seq samples. Higher red color intensities indicate higher eigengene values. (B) Heatmap of ATAC-seq co-open chromatin modules eigengenes across all ATAC-seq samples. Higher red color intensities indicate higher eigengene values. (C) Pairwise Pearson correlation heatmap of module eigengene values for RNA-seq and ATAC-seq modules. (D) Hypergeometric enrichment p values for gene signatures of PP signatures, selected developmental gene ontologies, and gene signatures of selected intestinal and hepatic tissue/cell types. Only modules with enrichment p values <0.01 for any of the selected categories in (D) and (E) are plotted. Full list is available in Table S1. (E) Hypergeometric enrichment p values for transcriptomic signatures of *in vivo* fetal pancreatic cell subpopulations from Ramond et al. (2018). Only modules with enrichment p values <0.01 for any of the selected categories in (D) and (E) are plotted. (F) Enrichment p values of selected known TF binding sites within modules of co-open chromatin. Only modules with enrichment p values <1e−20 for any of the selected TFs are plotted. (G) Selected highly correlated pairs of RNA-seq and ATAC-seq modules eigengenes. The height of the bars represents the eigengene values for each sample in the presented module. Bars are colored by each protocol/cell population, as outlined in figure legends for PCA plots in Figure 2B, and in other subsequent figures in this paper. I, protocol A GFP− module pair; II, protocol A GFP+ module pair; III, protocol B module pair; IV, protocol C module pair.

We next sought to determine the differences in the transcriptional and chromatin accessibility profiles of the PPs derived with the different protocols. We applied weighted gene co-expression network analysis (WGCNA) (Langfelder and Horvath, 2008) to our datasets, and conducted further analyses on the dimensionality reduced sets of modules combining correlated patterns of gene expression and open chromatin. We defined 20 modules of co-expressed genes from the RNA-seq data (Figure 4A, Table S2), as well as 11 modules of co-open chromatin sites from the ATAC-seq data (Figure 4B, Table S2). We then identified highly correlated pairs of RNA-seq and ATAC-seq modules, likely representing the modules of active open chromatin regulating the correlated genes’ expression (Figure 4C). For each of the gene expression modules, we performed hypergeometric testing of enrichment for significant overlap with gene signatures of PPs from this and previous studies (Cebola et al., 2015), genes involved in selected developmental processes (Figure 4D, Table S2), as well as gene signatures of fetal pancreas cell subpopulations (Ramond et al., 2018) (Figure 4E, Table S2). For each of the open chromatin modules, we identified TF binding motifs enriched within the module's peaks (Figure 4F). We highlight a few of the most interesting correlated RNA-ATAC gene module pairs in Figure 4G.

The most distinct gene expression and correlated open chromatin pattern observed was for RNA module R_M1 and ATAC module A_M1 (Figure 4GI). Both these modules had the highest eigengene values, corresponding to the first principal component and summarizing the overall pattern of gene expression for all genes in the module, in the GFP− cell population from protocol A. The R_M1 genes were enriched in genes involved in pancreas development, and very closely resembled the *in vivo* fetal endocrine progenitors, which was in line with the previous PCA analysis (Figure 3D). The A_M1 peaks were significantly enriched in binding sites of several TFs with known roles in pancreatic and endocrine development, including NEUROD1, PTF1A, RFX6, and FOXA2. This evidence corroborates that the GFP− population of protocol A represents a population of early endocrine progenitors, which is absent in the other two protocols.

Genes preferentially expressed in the GFP+ population of protocol A, and correlated open chromatin peaks, were captured in modules R_M5 and A_M5 (Figure 4GII). Importantly, these genes were also expressed in the GFP+ cell populations generated with protocols B and C, albeit at lower depth. The R_M5 genes were enriched in PP signatures derived earlier in this study, as well as in a previous study by Cebola and colleagues (Cebola et al., 2015), and were enriched in the transcriptomic signature of the *in vivo* multipotent PPs (Ramond et al., 2018). Open chromatin in module A_M5 was enriched in binding motifs of PDX1, SOX9, and PTF1a, all known as markers of PPs. This module pair represents the PP signatures preferential to protocol A but also present in the GFP+ populations from the other two protocols.

RNA modules R_M2 and R_M3 were both highly correlated with ATAC module A_M2 (Figure 4GIII) and represented genes preferentially expressed in all cell populations generated with protocol B. We noticed that the corresponding A_M2 open chromatin peaks were enriched in binding sites of HNF6 and CUX2, characteristic of the previously described PP open chromatin signature. However, we also noticed that the A_M2 module was the only module with significant enrichment in CDX2 binding sites. Based on previous reports of CDX2 regulating patterning of the intestinal epithelium (Grainger et al., 2010), we hypothesized that some of the cells differentiated with protocol B might be more likely to assume an intestinal fate. In line with this hypothesis, we found that R_M2 module genes were enriched in the intestinal gene signature of duodenum, and M3 genes in genes involved in digestive tract development and hepatic cords signature.

Finally, RNA gene modules M11, M13, and M16 were preferentially expressed in cells generated with protocol C. All these gene modules were correlated with ATAC module M4, particularly enriched for the FOSL2 binding sites. FOSL2 has not previously been implicated in pancreatic development. M16 module genes were enriched in the transcriptomic signature of the *in vivo* fetal multipotent PPs; however, both modules M11 and M16 showed enrichment in ileum gene signature, and M13 additionally in hepatic cords signature.

Given the magnitude of differences between the PPs derived with the different protocols reported here, we hypothesized that among the differentially expressed genes we might also find genes with well-established roles in pancreatic and endocrine development, and therefore of particular interest to the scientific community. We therefore investigated in more detail the gene expression profiles of genes previously implicated in maturity-onset diabetes of the young and neonatal diabetes (Flanagan et al., 2014, Yang and Chan, 2016) (Figure S3). For several genes we observed significant differences in the magnitude of expression between protocols, but we also found that some genes were only highly expressed in one or two of the protocols (e.g., *NKX2.2* in protocol A, *PPARG* in protocol B, *NEUROG3* in protocols A and C). We also observed some notable differences in the sorted cell population where a gene was preferentially expressed (e.g., the *RFX6* gene was enriched in the GFP− population of protocols A and C, while for protocol B both the GFP+ and GFP− cell population expressed *RFX6*). We anticipate that findings of any hPSC disease modeling studies for the genes highlighted here would be highly dependent on the choice of differentiation protocol.

In summary, analysis of pairs of correlated modules of gene expression and open chromatin provide an objective way to characterize the cell populations common and specific to each protocol. In addition, through gene ontology and transcription motif enrichment analyses, we were able to highlight the distinct developmental programs active in different protocols, as well as the TFs likely to control them.

### Variable Efficiency of Differentiation of PPs Toward the Endocrine Lineage

We next sought to evaluate the ability of the PPs derived from the three protocols to differentiate toward the pancreatic endocrine lineage. To this end, we applied stage 5 and stage 6 of the pancreas endocrine differentiation protocol developed by Rezania et al. (2014) (Figure S4A). While this protocol did not give rise to mature endocrine cells in our hands (Figure S4B), we have previously shown it to be useful for studying endocrine development *in vitro* (Petersen et al., 2017, Ramond et al., 2018). The endocrine differentiation was evaluated for expression of the endocrine markers NEUROD1 and NKX2.2 at stage 5 and for expression of C-peptide, a marker of endogenous insulin production, at stage 6. Interestingly, protocol A displayed expression of NEUROD1 and NKX2.2 in a significant percentage of cells after three days of differentiation, whereas the endocrine induction from protocol B and C was significantly lower (Figures S4C and S4D). The poor endocrine induction of protocols B and C was also evident at stage 6 of the differentiation protocol, where only very few C-peptide+ cells were observed from protocols B and C (Figures S4E–S4G). In contrast, protocol A displayed a robust induction of C-peptide+ cells at stage 6 of the protocol, with several of C-peptide+ cells co-expressing NKX6.1, indicative of differentiation toward beta-like cells (Figures S4E–S4G).

### Improved Pancreatic Endocrine Differentiation Following Reduction of the Intestinal Marker CDX2

**Figure 5:**
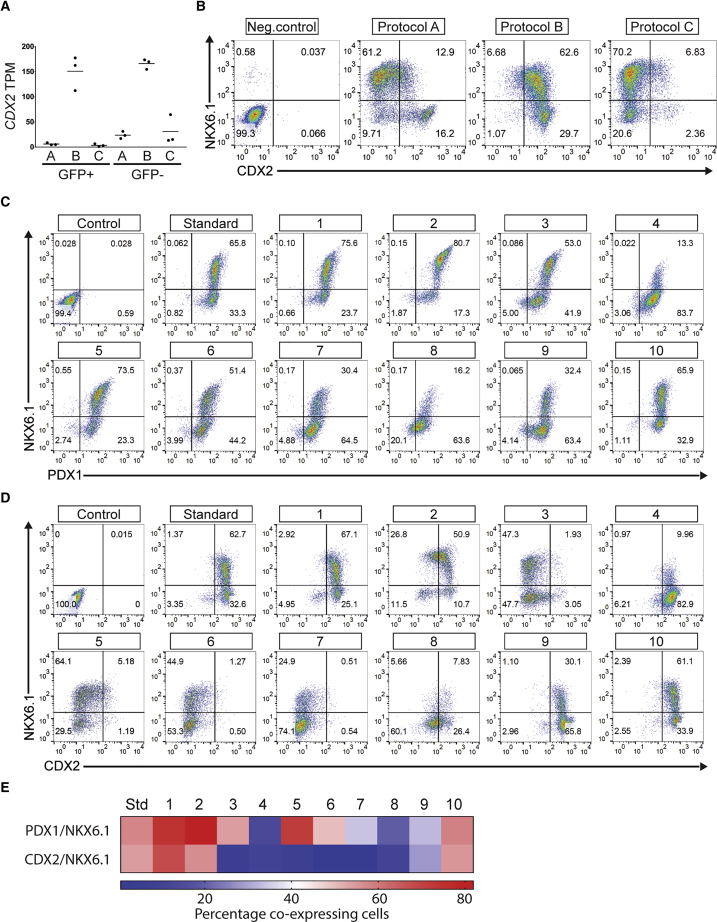
Reduction of Expression of the Intestinal Marker CDX2 in PPs (A) TPM for *CDX2* in GFP− and GFP+ sorted cell populations from differentiation protocols A, B, and C. Graph shows scatterplot of mean with each dot representing individual differentiations. (B) Flow cytometry analysis of NKX6.1 and CDX2 of PPs from the three differentiation protocols. DE was used as negative control. Representative pseudo color dot plots of five individual differentiations. (C and D) Representative pseudo color dot plots of two individual differentiations. Ten modifications of protocol B were assessed for the ability to maintain PDX1 and NKX6.1 expression (C) while simultaneously reducing expression of CDX2 (D). Representative pseudo color dot plots of cells stained for NKX6.1 and PDX1 (C) or NKX6.1 and CDX2 (D). DE cells were used as negative controls. (E) Heatmap summarizing the percentage of PDX1/NKX6.1 and CDX2/NKX6.1 co-expressing cells. Average percentage of one differentiation each of SB AD3.1 and SB AD3.4 hiPSC lines (n = 2 independent differentiations). Conditions tested were (1) 50 ng/mL Noggin, ST2; (2) 50 ng/mL Noggin, ST2-3; (3) 50 ng/mL Noggin, ST2-4; (4) ST4 only 2 days; (5) 100 nM LDN, ST2; (6) 100 nM LDN, ST2-3; (7) 100 nM LDN, ST2-4; (8) 50 ng/mL Activin A, ST4.2; (9) without KGF, ST4.2; (10) reduced retinoic concentration (0.2 μM) second day of ST3.

We next examined whether the RNA-seq and ATAC-seq profiles of the PPs could inform us on how to improve the endocrine differentiation. As described previously, we identified a gene module R_M2 preferentially expressed in protocol B that showed enrichment in an intestinal gene expression signature. We found that the correlated open chromatin module A_M2 was enriched in binding sites of the canonical intestinal TF CDX2. We confirmed that CDX2 was a member of the R_M2 gene module, and its expression was enriched in the NKX6.1+ cells of protocol B compared with protocols A and C (Figures 5A and 5B). We also observed CDX2-positive cells in protocol A, but in contrast to protocol B these were primarily NKX6.1-negative cells (Figures 5A, 5B, and S5). Omission of the BMP antagonist from protocol A led to a substantial increase in the CDX2-positive cells at the expense of NKX6.1-positive cells, suggesting that BMP signaling must be actively inhibited to prevent differentiation toward intestinal lineages. In contrast, addition of EGF during stage 4 of protocol A resulted in a significant reduction in CDX2-positive cells compared with the standard protocol A (Figure S5).

We hypothesized that the intestinal gene expression signature detected in PPs of protocol B could explain the low efficiency of differentiation toward the endocrine lineage (Figures S4C and S4D). To test this hypothesis, we evaluated ten modifications of protocol B, including several conditions with BMP antagonists aiming at maintaining the expression of the PP markers PDX1 and NKX6.1, while reducing the expression of CDX2. Interestingly, several of the modified conditions of protocol B maintained a high proportion of PDX1 and NKX6.1-positive cells (Figures 5C and 5E), but only two of these conditions (condition 3 and 5) also demonstrated a substantial reduction in CDX2 expression (Figures 5D and 5E). We subsequently evaluated the expression of select markers of the pancreatic and intestinal lineage in one of the modifications of protocol B resulting in reduction of CDX2 expression (condition 3). As expected, *CDX2* mRNA expression was downregulated in the modified protocol, as were several other genes from the intestinal gene expression signature, while several genes related to pancreas development were upregulated (Figure S5D).

**Figure 6:**
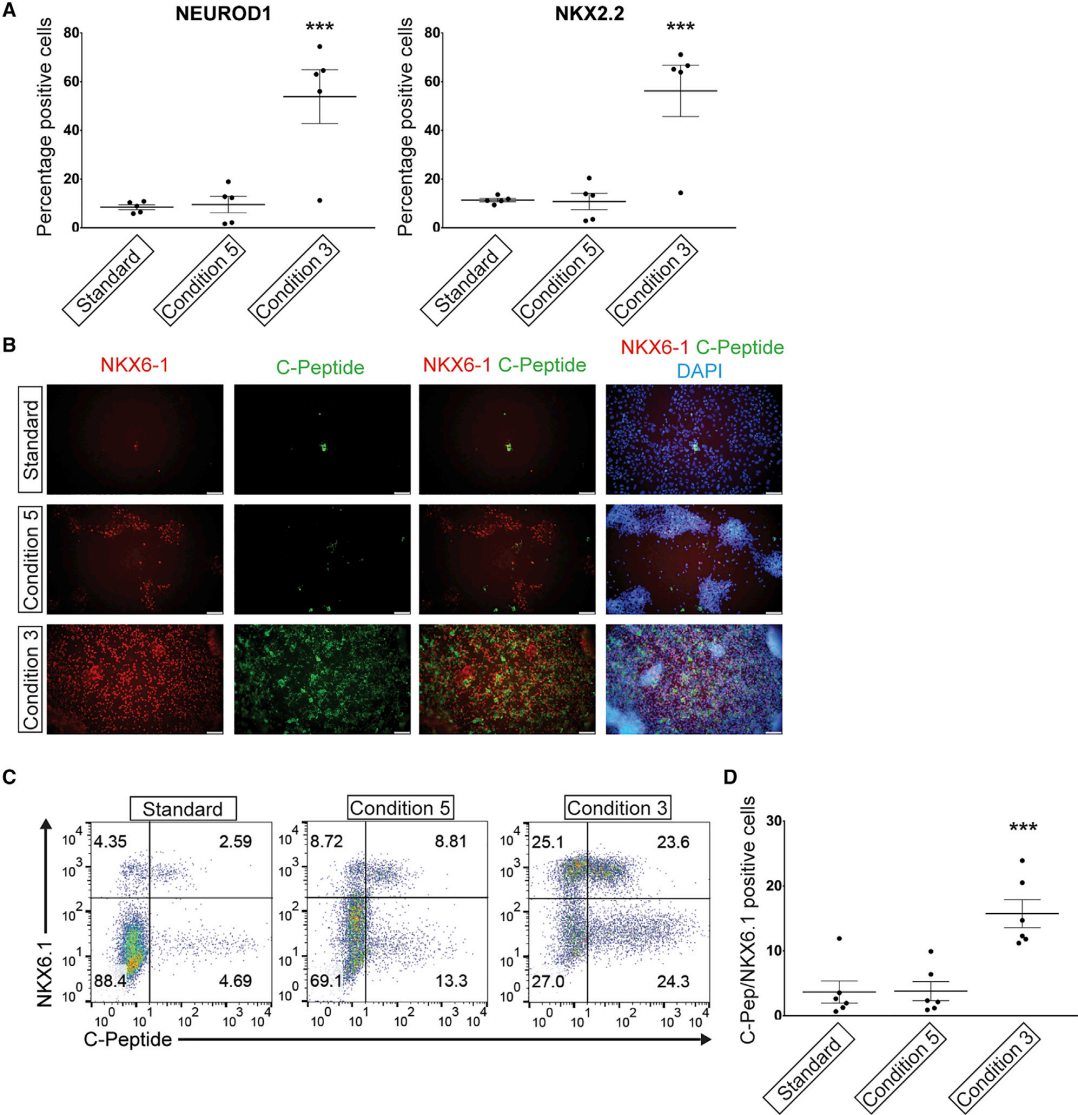
Improved Endocrine Differentiation Following Reduction of CDX2 Expression in PPs (A) Flow cytometry-based quantifications of stage 5 endocrine progenitor differentiation of protocol B and the two modified conditions (condition 5 and 3). Scatterplots show percentage of NEUROD1 and NKX2.2-positive cells as means ± SEM, n = 5 independent experiments (three with SB AD3.1 hiPSC, one with SB AD3.4 hiPSC, and one with SA121 hESC). (B) Immunofluorescence microscopy images of stage 6 cells stained with NKX6.1 and C-peptide antibodies. DAPI is used to visualize the nuclei of all cells. Scale bar, 100 μm. (C) Representative pseudo color dot plots of stage 6 beta-like cells stained for C-peptide and NKX6.1. Numbers mark the percentage of cells in each quadrant. (D) Quantification of C-peptide/NKX6.1 double positive by flow cytometry as shown in (C) (conditions 5 and 3). Scatterplot shows percentage of C-peptide/NKX6.1 double-positive cells as means ± SEM, n = 6 independent experiments (four with SB AD3.1 hiPSC, two with SA121 hESC). (A and D) One-way ANOVA with Tukey test for multiple comparisons, ^∗∗∗^p < 0.001, different from the two other groups.

We then tested whether the reduced CDX2 expression in protocol B resulted in improved ability to differentiate toward the pancreatic endocrine lineage. To this end, we differentiated PPs with protocol B, as well as its two modifications resulting in reduced CDX2 expression toward endocrine progenitors and endocrine cells, as described above (Figure S4A). Interestingly, inclusion of Noggin in stage 2–4 of protocol B (condition 3) resulted in a significant increase in the number of cells expressing the endocrine progenitor markers NEUROD1 and NKX2.2 (Figure 6A), compared with protocol B without the presence of a BMP inhibitor. Despite the reduced CDX2 expression in PPs in condition 5, this did not result in an improved differentiation toward the endocrine lineage (Figure 6A). To test whether the increased endocrine differentiation observed with the inclusion of Noggin in stage 2–4 of protocol B led to an increase in generation of beta-like cells, the cells were differentiated to the end of stage 6 (Rezania et al., 2014) (Figure S4A). We observed a significant increase in cells co-expressing the beta cell markers C-peptide and NKX6.1, when Noggin was included during stage 2–4 of protocol B (condition 3) compared with the standard protocol B (Figures 6B–6D).

## Discussion

In the present study, we applied three differentiation protocols for deriving PPs from hPSCs (Nostro et al., 2015, Rezania et al., 2014, Russ et al., 2015). All three protocols allowed for efficient generation of hPSC-derived PPs at levels comparable with the original reports. Interestingly, we noticed that certain hPSC lines displayed varying efficiency depending on the protocol applied. Variation in the ability to differentiate toward various lineages across hPSC lines has previously been reported (Osafune et al., 2008, Rouhani et al., 2014); however, our study highlights that such differences could possibly also be specific to the protocol used for deriving a particular cell lineage. We further demonstrated that modifications can be made to protocols, allowing for increasing the efficiency of otherwise poorly differentiating hPSC lines. Exclusion of Noggin during stage 2 rescued the ability of one hPSC line to generate PPs with protocol C. Previous studies investigated the effect of modulating the BMP signaling pathway during specification to the pancreatic lineage (Nostro et al., 2011, Shahjalal et al., 2014) but our results suggest that BMP signaling may influence the cell lineage choice at even earlier stages of differentiation. The effect of Noggin appeared to be dependent on the differentiation protocols but also of the hPSC lines applied. This suggests that variation in endogenous BMP signaling during the early stages of differentiation can influence the choice of fate of hPSC lines and modulation thereof could be an avenue for further protocol optimization for hPSC lines that prove difficult to differentiate toward the pancreatic lineage.

We performed RNA-seq and ATAC-seq of isolated NKX6.1-positive cells, which allowed us to define a transcriptomic and epigenomic signature for *in vitro*-generated PPs from the three differentiation protocols, through comparison with equivalent data from all the stages of hPSC differentiation toward beta-like cells. Of note, this approach excludes genes expressed at multiple stages of the hPSC differentiation toward beta-like cells and therefore not unique to the PP stage, including *PDX1* and *NKX6.1*, which are continuously expressed from the posterior foregut and pancreatic progenitor stages of the differentiation protocol, respectively. Globally, the gene expression and chromatin accessibility profiles for cells generated with all three protocols clustered together with the pancreatic endoderm stage samples of the control lines. *In vivo* differentiation of hPSC-derived PPs to the specific lineages of the pancreas is considered the most stringent method for evaluating the competence of these cells. Thus, one limitation in the current study is that we did not evaluate the competence of the PPs from the three protocols *in vivo*. Nonetheless, the PPs of protocols A and C showed significant similarities to the *in vivo* PPs derived from human fetal pancreas, suggesting that these protocols recapitulate several aspects of human pancreas development. Among the genes forming the *in vitro* PP signature we found several genes involved in pancreas development, including *ONECUT1* (Jacquemin et al., 2003) and *SOX9* (Seymour et al., 2007), as well as genes involved in Notch and Wnt signaling pathways, with previously well-established functions in pancreatic development (Pan and Wright, 2011). The open chromatin regions forming the epigenomic PP signature were significantly enriched in binding motifs of several known pancreatic TFs, including HNF6, HNF1B, FOXA2, PDX1, and SOX9. We observed that CUX2 motifs were the most significantly enriched in these common open chromatin regions. In line with this, we found that the *CUX2* gene's expression during differentiation peaks at the pancreatic endoderm stage, and we observed evidence for CUX2 being bound within the open chromatin of the samples generated in this study through ATAC-seq footprinting analysis. Interestingly, a previous study demonstrated the expression of *CUX2* in early fetal human dorsal pancreas (Jennings et al., 2017), and more recently the presence of CUX2 motifs in accessible chromatin regions were shown to be differentially enriched in mouse endocrine progenitors across different stages of development (Scavuzzo et al., 2018). Based on this evidence, we suggest CUX2 as a potential novel regulator of pancreatic development.

We then sought to highlight the protocol-specific differences in the omics profiles of the PPs. We focused on modules of co-expressed genes and correlated open chromatin grouping genes preferentially expressed in each of the protocols. We observed a significantly higher endocrine commitment in the NKX6.1-negative compartment of protocol A compared with protocols B and C. This is in agreement with the original reports, where Nostro et al. (2015) and Russ et al. (2015) developed protocols with the specific purpose of limiting the endocrine differentiation in place of PPs. Thus, we were able to recapitulate important features of the three differentiation protocols. Interestingly, the NKX6.1-negative population of protocol A clustered closely together with isolated human fetal endocrine progenitors (Ramond et al., 2018), suggesting that the endocrine differentiation observed *in vitro* shares many similarities with fetal endocrine development. On the other hand, genes with the highest expression in the GFP+ population from protocol A were enriched in gene signatures of the *in vivo* fetal PPs from the same study, as well as in the signatures of *in vitro* PPs from this and a previous study (Cebola et al., 2015).

Further, we noted that some of the genes preferentially expressed in cells generated with protocol B were suggestive of these cells assuming a non-endocrine fate. We noted enrichments in genes forming previously reported intestinal signatures, and we observed that open chromatin regions correlated with expression of these genes were enriched in binding motifs of CDX2, a marker of intestinal development, and *CDX2* gene expression was also most highly expressed in protocol B cells. A recent study reported the expression of *CDX2* in the dorsal pancreas of human fetal tissue (Jennings et al., 2017). However, we have previously performed single-cell gene expression analysis of human fetal pancreata and found no evidence of *CDX2* expression in human PPs (Ramond et al., 2018). This discrepancy could perhaps be explained by the different stages of human pancreas development interrogated in the two studies. Thus, it remains unclear whether PDX1, NKX6.1, and CDX2 co-expressing progenitor cells occur at some point during human pancreas development. CDX2-positive cells were also observed in protocol A but the majority of these were NKX6.1 negative. Excluding the BMP antagonists from protocol A resulted in a significant increase of CDX2-positive cells at the expense of NKX6.1-positive PPs, illustrating that inhibiting BMP signaling is necessary to prevent differentiation toward an intestinal fate. This observation is in agreement with a previous study demonstrating that BMP signaling inhibition during differentiation of hPSCs to PPs resulted in reduced *CDX2* expression (Shahjalal et al., 2014). Interestingly, addition of EGF to the final stage of protocol A resulted in a reduction of the percentage of CDX2-positive cells. These results illustrate how evaluating individual components of a differentiation protocol can elucidate their specific roles in the differentiation and guide improving the protocol efficiency.

We hypothesized that *CDX2* expression and the intestinal gene signature enriched in the PPs from protocol B could suggest a more posterior patterning during the differentiation, which might explain the inability of the PPs from protocol B to differentiate further toward the pancreatic endocrine lineage. In the present study, we found that inclusion of the BMP antagonist Noggin resulted in reduced expression of *CDX2* and other genes specific to the intestinal gene expression module, and subsequently in successful differentiation down the endocrine lineage. Interestingly, another BMP antagonist, LDN193189, also reduced CDX2 expression, but this was not accompanied by an improved differentiation toward the endocrine lineage. The reason for this difference is unclear but may relate to the different mechanisms of the two BMP antagonists. Together, these results illustrate the importance of monitoring CDX2 expression during differentiation toward PPs. Interestingly, the original report describing protocol B argued for the omission of BMP antagonist during pancreatic specification in order for efficient derivation of PDX1/NKX6.1 co-expressing PPs (Russ et al., 2015); however, CDX2 expression was not assessed in this study. It thus remains unclear whether CDX2 expression in the PPs is inherent to this protocol or a consequence of differences in experimental conditions and cell lines applied in this study compared with the original study (Russ et al., 2015). It is also plausible that, since the endocrine differentiation protocol applied in this study was originally developed in combination with protocol A (Rezania et al., 2014), it may not be directly applicable to other protocols. Whether there are differences in the signaling pathways that promote endocrine differentiation from the PPs derived from protocols A, B, and C remains to be shown.

There are many challenges associated with faithfully reproducing differentiation protocols, most recently demonstrated in a study comparing iPSC-derived neurons using a well-defined protocol across five different laboratories (Volpato et al., 2018). In addition, several modifications were made to the protocols applied in the current study compared with the original publications. Thus, it should be emphasized that our study should not be seen as a direct head-to-head comparison of the three protocols. Nonetheless, our work demonstrates the importance of assessing apparently similar cell populations derived using distinct differentiation protocols. This approach allowed us to define a comprehensive transcriptional and epigenomic signature for PPs that will serve as a valuable resource for studying pancreas development. However, our findings also highlighted significant variation in the PPs derived from the three protocols, which should warrant caution for using hPSCs to interrogate roles of specific genes in disease and development. Finally, we demonstrate the utility of benchmarking *in vitro*-derived cell populations to their *in vivo* counterparts, which allows for the identification of markers useful for improving the differentiation of hPSCs toward beta-like cells.

## Experimental Procedures

Please refer to Supplemental Experimental Procedures for detailed description of experimental procedures.

### Maintenance and Differentiation of hPSC Lines

All hPSCs were maintained on human embryonic stem cell-qualified Matrigel in mTeSR1 medium, except for the NKX6.1-GFP hiPSC line, which was cultured in TeSR1-E8 medium, at 37°C, 5% CO_2_, and passaged at 90%–95% confluence using TrypLE select, as previously described (Perez-Alcantara et al., 2018).

### FACS

Briefly, differentiated cells were harvested to a single-cell suspension using TrypLE select, pelleted and resuspended in the stage 4 medium of the respective protocols (without factors) containing 1 μg/mL DAPI solution, and directly proceeded for sorting. Cells were sorted using a BD FACSAria Fusion (BD Biosciences).

### RNA-seq

For RNA-seq, cells were pelleted immediately following sorting and medium was removed. Cells were harvested and RNA extracted using TRIzol Reagent (ThermoFisher Scientific, Paisley, UK) as per the manufacturer's guidelines. Smart-Seq2 paired-end RNA-seq libraries were sequenced on Illumina HiSeq4000 to a mean depth of 37.7 (± 1.8) million 75 bp reads pairs per sample.

### Transposition Reaction and Purification for ATAC-Seq

Following sorting, cells were pelleted and washed in cold PBS. Cell pellets were gently resuspended in cold lysis buffer (10 mM Tris-HCL, pH 7.4, 10 mM NaCl, 3 mM MgCl_2_, 0.1% IGEPAL CA-630) and immediately pelleted by centrifugation. The supernatant was discarded and the cell pellets were gently resuspended in 50 μL of transposition reaction mix (25 μL 2 of TD buffer, 2.5 μL of Tn5 Transposase [Illumina], 22.5 μL of nuclease-free H_2_O) and incubated at 37°C for 30 min. Following the transposition reaction, the transposed DNA was purified using QIAGEN's MinElute Kit according to manufacturer's instructions and eluted in 10 μL of elution buffer (10 mM Tris buffer, pH 8).

### Statistics

Statistical analyses were performed using GraphPad Prism (V8.0.2). Means were compared with unpaired Student's t test and one-way ANOVA with Tukey test for multiple comparisons.

## Author Contributions

A.W.A. performed all bioinformatics analysis, interpreted the data, and wrote the manuscript. R.R.J. performed *in vitro* differentiation experiments, data analysis, and interpreted data. M.P.A. provided data for ATAC-seq analysis of control iPSC lines and performed bioinformatics analysis of these. N.L.B., C.D., V.N., M.G., L.W., and R.B. provided experimental and scientific input. M.I.M., M.H. and A.L.G. provided scientific input and edited the manuscript. C.H. performed *in vitro* differentiation experiments, data analysis, interpreted data, and wrote the manuscript. All authors approved the manuscript.

## Accession Numbers

RNA-seq and ATAC-seq raw data have been deposited in the European Genome-phenome Archive, accession number EGAS00001003513. Dataset (RNA-seq): EGAS00001004823. Dataset (ATAC-seq): EGAS00001004824.

## Supplemental Information

Document S1. Supplemental Experimental Procedures and Figures S1–S5

Table S1. Common Pancreatic Progenitor Signature

Table S2. Modules of Co-expression and Open Chromatin

Document S2. Article plus Supplemental Information
