# Supplementary material for: Analysis of Differentiation Protocols Defines a Common Pancreatic Progenitor Molecular Signature and Guides Refinement of Endocrine Differentiation
Source: Stem Cell Reports. 2019 Dec 26;14(1):138–53. doi: 10.1016/j.stemcr.2019.11.010 (PMC6962645; doi:10.1016/j.stemcr.2019.11.010)
Supplement: Document S1. Supplemental Experimental Procedures and Figures S1–S5 [file mmc1.pdf]

Stem Cell Reports, Volume *14*

Supplemental Information

Analysis of Differentiation Protocols Defines a Common Pancreatic Progenitor Molecular Signature and Guides Refinement of Endocrine Differentiation

Agata Wesolowska-Andersen, Rikke Rejnholdt Jensen, Marta Pérez Alcántara, Nicola L. Beer, Claire Duff, Vibe Nylander, Matthew Gosden, Lorna Witty, Rory Bowden, Mark I. McCarthy, Mattias Hansson, Anna L. Gloyn, and Christian Honore

SUPPLEMENTAL FIGURES

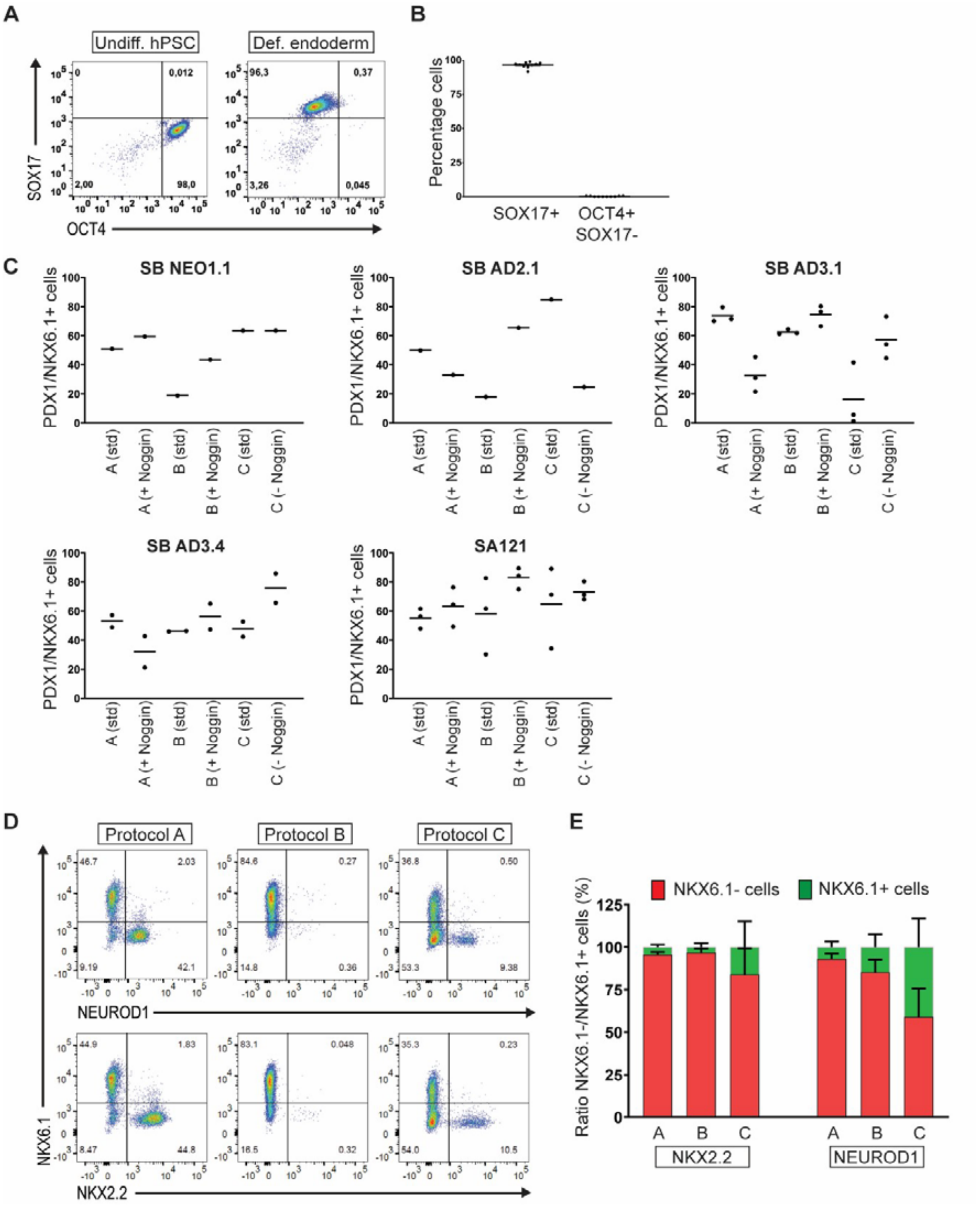

Supplementary figure 1. Definitive endoderm and pancreatic progenitor differentiation efficiency. Related to figure 1.

(A) Representative example of flow cytometry pseudo color dot plots of undifferentiated hPSC and definitive endoderm cells stained for OCT4 and SOX17. (B) Percentage of SOX17+ cells and OCT4+, SOX17- cells at the definitive endoderm stage of the protocol. Data presented as mean ± SEM, n = 10 independent experiments (SA121 hESC n = 3, SB NEO1.1 hiPSC n = 1, SB AD2.1 hiPSC n = 1, SB AD3.1 hiPSC n = 3, SB AD3.4 hiPSC n = 2). (C) Quantification of PDX1 and NKX6.1 co-expressing cells across five individual hPSC lines differentiated with protocol A, B and C. The effect of inclusion/omission of Noggin during stage 2 of each protocol was evaluated side-by-side compared to the standard protocols (outlined in figure 1A). Graphs show scatter plot of mean, n = 1-3 independent experiments, depending on cell line. (D) representative example of flow cytometry pseudo color dot plots of hPSC-derived pancreatic progenitors from protocol A, B and C. Cells are analysed for NKX6.1/NEUROD1 (top) and NKX6,1/NKX2.2 expression (bottom). (E) Ratio of NKX2.2 and NEUROD1 expressing cells negative (red) or positive (green) for NKX6.1 expression. Data presented as mean ± SEM, n = 5 independent experiments.

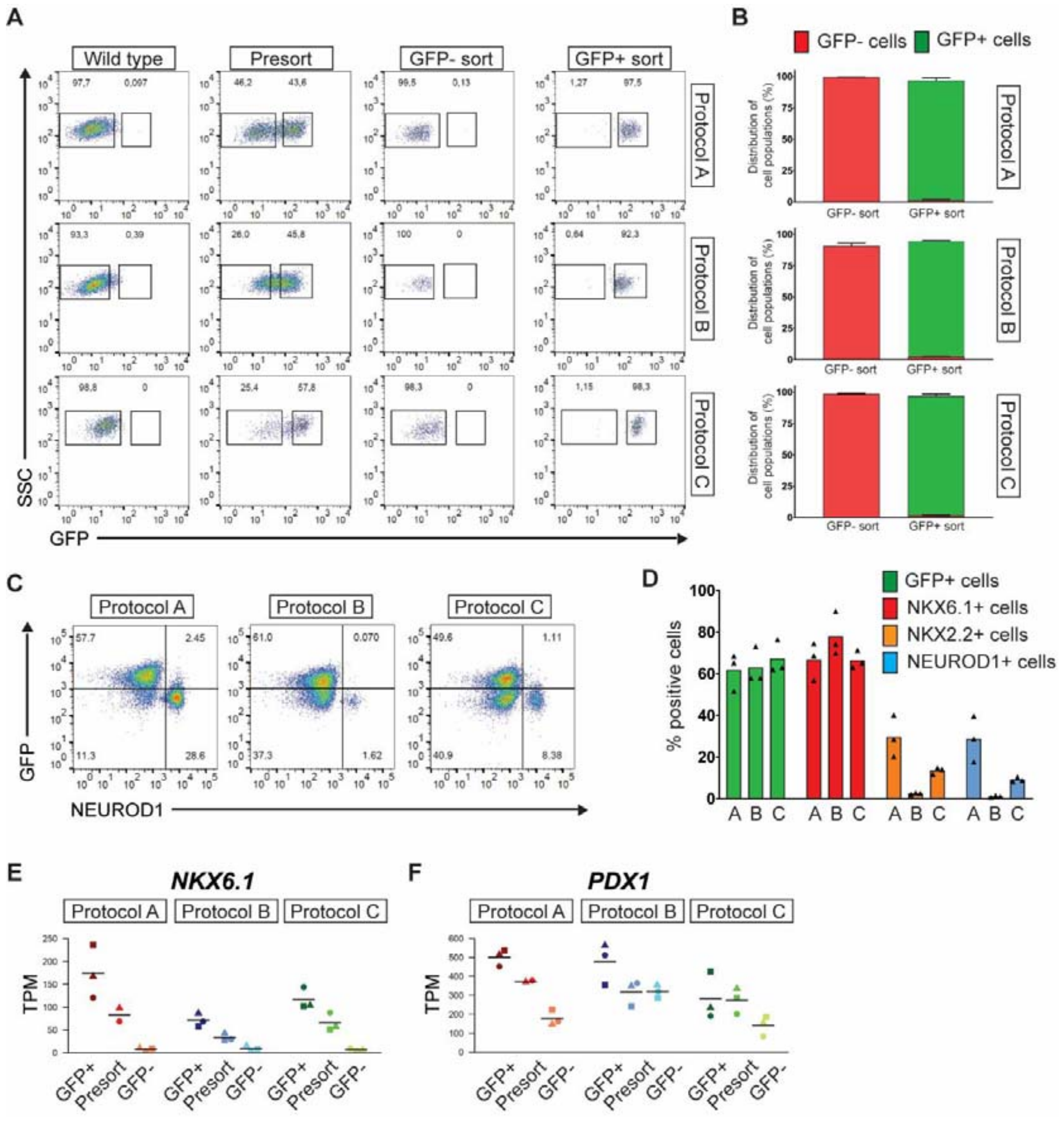

Supplementary figure 2. Confirmation of isolation of NKX6.1+ and NKX6.1- cell populations by FACS. Related to figure 2.

(A) Representative example of flow cytometry pseudo color dot plots of wild type hiPSC line (wild type), presorted NKX6.1-GFP iPSC (presort) and the GFP- and GFP+ sorted fractions from all three protocols analyzed for purity post sorting. Boxes indicates gates labelling GFP+ and GFP- cell fractions (B) Graph showing the percentage of GFP- and GFP+ cells in each of the two sorted fractions across all three differentiation protocols. Graphs shows mean ± SEM of three independent differentiations/sorting experiments (n = 3). (C) Representative examples of pseudo color dot plots of NKX6.1-GFP hiPSC line differentiated to pancreatic progenitors using the three protocols. Cells are analysed for GFP and NEUROD1 expression and illustrates that the endocrine cells (NEUROD1+ cells) are mainly found in the GFP- (NKX6.1-) compartment. (D) Flow cytometry quantification of GFP+, NKX6.1+, NKX2.2+ and NEUROD1+ cells from the three experiments used for the cell sorting experiments. Bars show mean and dots represent individual differentiations (n = 3). GFP+ expression was assessed on live cells, whereas NKX6.1, NKX2.2 and NEUROD1 expression analysis was performed on fixed and permeabilized cells. (E-F) Transcript per kilobase million (TPM) for *NKX6.1* and *PDX1* in presorted, GFP- and GFP+ sorted cell populations across all three differentiation protocols. Graphs shows scatter plot and mean, with each symbol representing an independent differentiation. *NKX6.1*: p_{adjA}=4.05e-101, p_{adjB}=1.60e-27, p_{adjC}=5.97e-64; *PDX1*: p_{adjA} =2.70e-19, p_{adjB} =8.42e-03, p_{adjC} =1.20e-07.

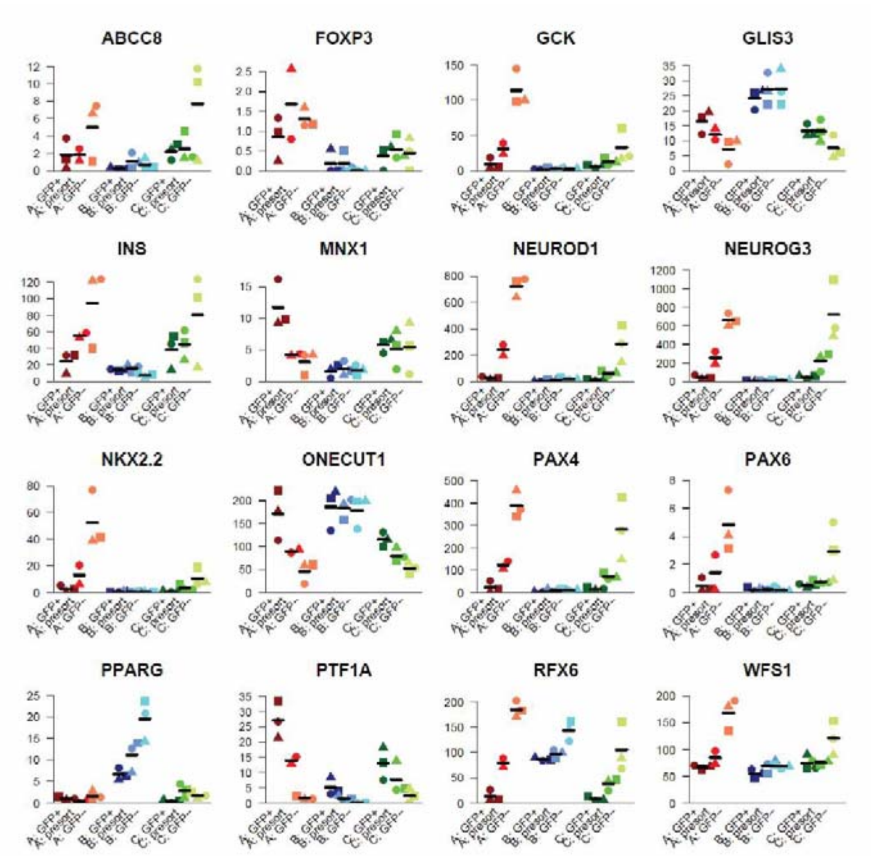

Supplementary figure 3. Expression of selected genes implicated in MODY and neonatal diabetes. Related to figure 4. Expression is shown as transcript per kilobase million (TPM) in pre-sorted, GFP- and GFP+ sorted cell populations across all three differentiation protocols. Graphs shows scatter plot and mean, with each symbol representing an independent differentiation.

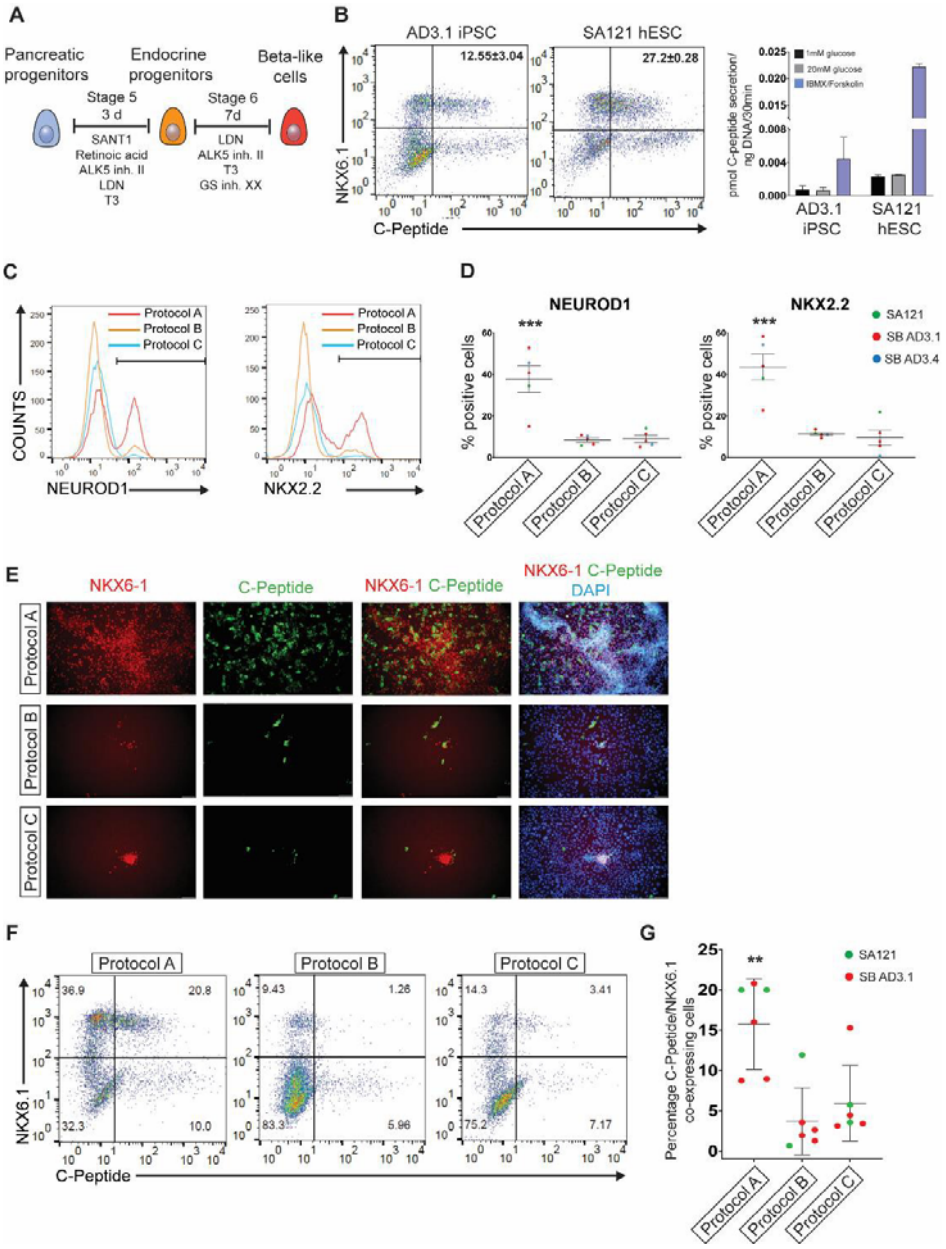
A
Pancreatic progenitors
Endocrine progenitors
Beta-like cells
Stage 5
3 d
SANT1
Retinoic acid
ALK5 inh. II
LDN
T3
Stage 6
7d
LDN
ALK5 inh. II
T3
GS inh. XX
B
AD3.1 iPSC
SA121 hESC
12.55±3.04
27.2±0.28
NKX6.1
C-Peptide
pmol C-peptide secretion/ ng DNA/30min
1mM glucose
20mM glucose
IBMX/Forskolin
AD3.1 iPSC
SA121 hESC
C
Protocol A
Protocol B
Protocol C
COUNTS
NEUROD1
NKX2.2
D
NEUROD1
NKX2.2
% positive cells
***
SA121
SB AD3.1
SB AD3.4
Protocol A
Protocol B
Protocol C
E
NKX6-1
C-Peptide
NKX6-1 C-Peptide
NKX6-1 C-Peptide DAPI
Protocol A
Protocol B
Protocol C
F
Protocol A
Protocol B
Protocol C
36.9
20.8
32.3
10.0
9.43
1.26
83.3
5.96
14.3
3.41
75.2
7.17
NKX6.1
C-Peptide
G
Percentage C-Ppetide/NKX6.1 co-expressing cells
**
SA121
SB AD3.1
Protocol A
Protocol B
Protocol C

Supplementary figure 4. Pancreatic endocrine differentiation of pancreatic progenitors derived from multiple protocols. **Related to figure 5**.

(A) Schematic outline of the protocol for differentiating the pancreatic progenitors from the three protocols towards first endocrine progenitors (stage 5) and subsequently towards beta-like cells (stage 6). (B) NKX6.1/C-Peptide flow cytometry pseudo color dot plots of AD3.1 iPSC and SA121 hESC lines differentiated to stage 6 for subsequent glucose stimulated insulin secretion (GSIS). Numbers in upper right quadrant shows percentage of NKX6.1+, C-Peptide+ cells (mean ± SD, n =2 for each line). Graph shows C-Peptide secretion normalized to DNA content of cells (mean ± SD, n =2 for each line). (C) Representative flow cytometry histograms demonstrating the quantification of NEUROD1 (left) and NKX2.2 (right) positive cells. Gate set based on a negative cell population and unstained cells. Color of the lines mark cells from each of the three protocols. Horizontal bar in both plots shows the gate for positive cells (D) Quantifications of NEUROD1 and NKX2.2 positive cells as shown in (C). Graphs shows percentage of NEUROD1 and NKX2.2 positive cells as mean ± SEM, n = 5 independent experiments (E) Immunofluorescence microscopy images of stage 6 cells derived from pancreatic progenitors from protocol A, B and C. Cells were stained with NKX6.1 and C-Peptide antibodies. DAPI is used to visualize the nuclei of all cells. Scalebar = 100µM. (F) Representative flow cytometry pseudo color dot plots of stage 6 cells derived from each of the three protocols. (G) Quantification of C-Peptide, NKX6.1 co-expressing cells as mean ± SEM, n = 6 independent experiments. For (D) and (G) Dots are color coded according to individual cell lines as indicated in legend of the figure. One-way ANOVA with Tukey test for multiple comparisons, $^{**}P < 0.01$, $^{***}P < 0.001$, different from the two other groups.

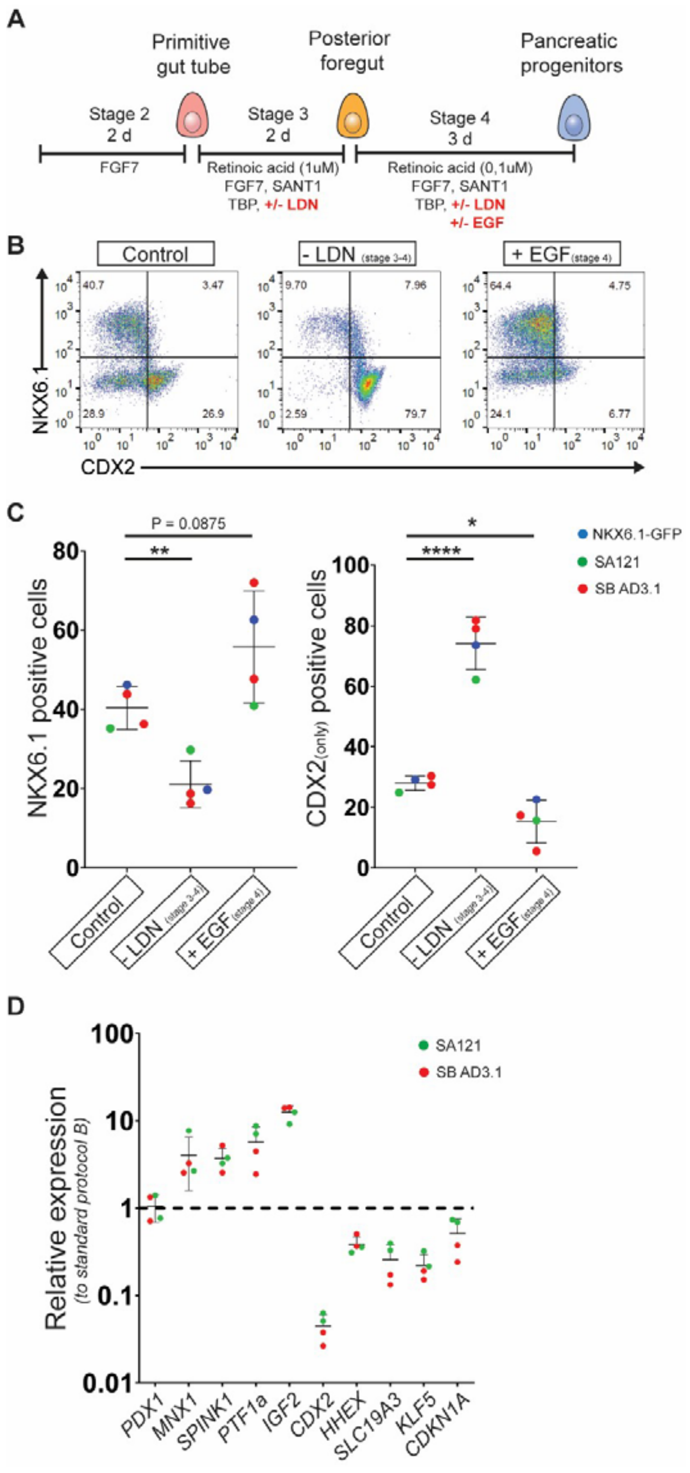

A
Primitive gut tube
Posterior foregut
Pancreatic progenitors
Stage 2 2 d
FGF7
Stage 3 2 d
Retinoic acid (1uM) FGF7, SANT1 TBP, +/- LDN
Stage 4 3 d
Retinoic acid (0,1uM) FGF7, SANT1 TBP, +/- LDN +/- EGF
B
Control
- LDN (stage 3-4)
+ EGF (stage 4)
NKX6.1
CDX2
40.7 3.47 28.9 26.9
9.70 7.96 2.59 79.7
64.4 4.75 24.1 6.77
C
P = 0.0875
**
*
****
NKX6.1 positive cells
CDX2(only) positive cells
NKX6.1-GFP
SA121
SB AD3.1
Control
- LDN (stage 3-4)
+ EGF (stage 4)
D
SA121
SB AD3.1
Relative expression (to standard protocol B)
PDX1 MNX1 SPINK1 PTF1a IGF2 CDX2 HHEX SLC19A3 KLF5 CDKN1A

Supplementary figure 5. Evaluation of BMP and EGF signalling in differentiation protocol A and B. Related to figure 5.

(A) Schematic outline of stage 2-4 of protocol A. Red text highlights compounds evaluated. (B) Representative examples of pseudo color dot plots of cells at the end of stage 4 differentiated with protocol A and modifications thereof. (C) Quantification of NKX6.1 positive (left) and $CDX2_{(only)}$ positive cells (right). Graphs shows percentage of positive cells as mean ± SEM, n = 4 independent experiments. Dots are color coded according to individual cell lines as indicated in legend of the figure. Unpaired t test applied to determine differences between individual groups. *P < 0.05, **P < 0.01, ****P < 0.0001. (D) Quantitative gene expression analysis of pancreatic progenitors derived using protocol B or a modified version of protocol B (Condition 3 in figure 5). Expression was normalized to the average expression of two housekeeping genes (*ACTB* and *HPRT1*). Figure shows expression of genes in the modified version of protocol B relative to the standard protocol B (dashed line). Mean ± SEM, n = 4.

RESOURCE TABLE
Reagents, antibodies, assays, cell lines and software applied.

REAGENT or RESOURCE	SOURCE	IDENTIFIER
Antibodies		
NKX6.1 Alexa 647 (FC 1:40)	BD Biosciences	Cat#563338; RRID: N/A
NKX6.1 PE (FC 1:40)	BD Biosciences	Cat#563023; RRID:AB_2716792
PDX1 Alexa 488 (FC 1:40)	BD Biosciences	Cat#562274; RRID:AB_10924596
NeuroD1 PE (FC 1:40)	BD Biosciences	Cat#563001; RRID:AB_2716791
NKX2.2 PE (FC 1:40)	BD Biosciences	Cat#564730; RRID: N/A
Ki-67 Alexa 488 (FC 1:20)	BD Biosciences	Cat#558616; RRID:AB_647087
Sox17 Alexa 488 (FC 1:40)	BD Biosciences	Cat#562205; RRID:AB_10893402
Oct3/4 Alexa 647 (FC 1:10)	BD Biosciences	Cat#560307; RRID:AB_1645319
CDX2 (FC 1:500)	ThermoFisher	Cat#RM-2116; RRID: N/A; clone EPR2764Y
Rabbit IgG (isotype control)	R&D systems	Cat#AB-105-C; RRID:AB_354266
PDX1 (IF 1:8000)	Abcam	Cat#AB47383; RRID:AB_2162359
NKX6.1 (IF 1:200)	DSHB	Cat#F55A12-S; RRID:AB_532379
C-Peptide/Pro-Insulin (IF 1:250)	DSHB	Cat#GN-ID4; RRID:AB_2255626
Donkey Anti-Rabbit Alexa488 (FC 1:500)	ThermoFisher	Cat#A21206; RRID:AB_2535792
Donkey Anti-Goat Alexa488 (IF 1:500)	ThermoFisher	
Donkey Anti-Mouse Alexa594 (IF 1:500)	ThermoFisher	
Donkey Anti-Rat Alexa488 (IF 1:500)	ThermoFisher	
DAPI solution (FC)	BD Biosciences	Cat#564907
LIVE/DEAD™ Fixable Violet Dead Cell Stain Kit	ThermoFisher	Cat#L34955
NucleoSpin RNA/Protein kit	Macherey-Nagel	Cat#740933.50
iScript cDNA Synthesis Kit	Bio-rad	Cat#170-8890
TagMan Gene Expression Master Mix	ThermoFisher Scientific	Cat##PN 4369016
ACTB TaqMan Assay	ThermoFisher Scientific	hs01060665_g1
HPRT1 TaqMan Assay	ThermoFisher Scientific	hs99999909_m1
PDX1 TaqMan Assay	ThermoFisher Scientific	hs00236830_m1
MNX1 TaqMan Assay	ThermoFisher Scientific	hs00907365_m1
PTF1A TaqMan Assay	ThermoFisher Scientific	hs00603586_g1
IGF2 TaqMan Assay	ThermoFisher Scientific	hs04188276_m1
CDX2 TagMan Assay	ThermoFisher Scientific	Hs01078080_m1
HHEX TaqMan Assay	ThermoFisher Scientific	hs00242160_m1
SPINK1 TaqMan Assay	ThermoFisher Scientific	hs00162154_m1
SLC19A3 TaqMan Assay	ThermoFisher Scientific	hs00228858_m1

KLF5 TaqMan Assay	ThermoFisher Scientific	hs00156145_m1
CDKN1A TaqMan Assay	ThermoFisher Scientific	hs00355782_m1
Bacterial and Virus Strains		
Biological Samples		
Chemicals, Peptides, and Recombinant Proteins		
hESC-qualified matrigel	BD biosciences	Cat#354277
Growth factor reduced matrigel	BD biosciences	Cat#356230
mTeSR1 medium	Stemcell technologies	Cat#05850
TeSR1-E8 medium	Stemcell technologies	Cat#05990
MCDB131 medium	ThermoFisher	Cat# 10372019
DMEM/F12 medium	ThermoFisher	Cat# 21331020
DMEM(HG) medium	ThermoFisher	Cat# 31966021
PBS-/- (without calcium and magnesium)	ThermoFisher	Cat#14190094
PBS+/+ (with calcium and magnesium)	ThermoFisher	Cat# 14040133
TrypLE select	ThermoFisher	Cat#12563011
4% Formaldehyde	VWR	Cat#9713.1000; CAS 50-00-0
Triton X-100	Sigma-Aldrich	Cat#T9284; CAS 9002-93-1
Donkey serum	Millipore	Cat#S30-100ML
$NaHCO_3$	ThermoFisher	Cat#25080060
Penicillin-Streptomycin solution	ThermoFisher	Cat#15140122
Glutamax	ThermoFisher	Cat#35050038
Glucose	Fisher scientific	Cat#D16500
BSA, fatty acid free	Proliant	Cat#68700
BSA Fraction V, fatty acid free	Sigma-Aldrich	Cat#10775835001
ITS-X	ThermoFisher	Cat#51500056
Zinc sulphate	Sigma-Aldrich	Cat# Z0251; CAS 7446-20-0
B-27 supplement (50x)	ThermoFisher	Cat#17504044
B-27 supplement (50x), minus vitamin A	ThermoFisher	Cat#12587010
Rock inhibitor	Sigma-Aldrich	Cat#Y0503; CAS 129830-38-2
Activin A	Peprotech	Cat#120-14
CHIR99021	Axon Medchem	Cat#1386; CAS 252917-06-9
L-Ascorbic acid	Sigma-Aldrich	Cat#A4544; CAS 50-81-7
Recombinant human KGF (FGF-7)	Peprotech	Cat#100-19
Retinoic acid	Sigma-Aldrich	Cat#R2625; CAS 302-79-4
SANT-1	Sigma-Aldrich	Cat#S4572; CAS 304909-07-7
LDN-193189	Stemgent	Cat#04-0074; CAS 1062368-24-4
TBP (PKC activator)	Merck Millipore	Cat#565740; CAS 497259-23-1
Heparin	Sigma-Aldrich	Cat#H3149; CAS 9041-08-1
3,3′,5-Triiodo-L-thyronine sodium salt (T3)	Sigma-Aldrich	CAT# T6397; CAS 55-06-1

ALK5 Inhibitor II	Enzo life sciences	Cat# ALX-270-445; CAS 446859-33-2
γ-Secretase Inhibitor XX	Merck Millipore	Cat#565789; CAS 209984-56-5
Recombinant Human EGF	R&D systems	Cat#236-EG
Recombinant Human Noggin	Peprotech	Cat#120-10C
Nicotinamide	Sigma-Aldrich	Cat#N0636; CAS 98-92-0
Critical Commercial Assays		
Deposited Data		
EGA RNA-seq		Study ID: EGAS00001003513 Data set: EGAS00001004823
EGA ATAC-seq		Study ID: EGAS00001003513 Data set: EGAS00001004824
Experimental Models: Cell Lines		
SB NEO1 Cl. 1	https://www.stembancc.org/ https://www.ebisc.org/	DOI: 10.1007/s00125-018-4612-4
SB AD2.1 hiPSC	https://www.stembancc.org/ https://www.ebisc.org/	DOI: 10.1007/s00125-018-4612-4 DOI: 10.1080/19382014.2016.1182276
SB AD3.1 hiPSC	https://www.stembancc.org/ https://www.ebisc.org/	DOI: 10.1007/s00125-018-4612-4
SB AD3.4 hiPSC	https://www.stembancc.org/ https://www.ebisc.org/	DOI: 10.1080/19382014.2016.1182276
SA121 hESC	Takara Bio	Cat# Y00020
NKX6.1-GFP hiPSC (clone 1a-21-3)	https://www.stembancc.org/ https://www.ebisc.org/	DOI: 10.1016/j.scr.2018.04.010
Experimental Models: Organisms/Strains		
Oligonucleotides		
Recombinant DNA		
Software and Algorithms		
FASTQC		https://www.bioinformatics.babraham.ac.uk/projects/fastqc/

STAR (v.2.5.1)	DOI: 10.1093/bioinformatics/bts635	https://github.com/alexdobin/STAR
Picard tools suite (v2.1.1) - MarkDuplicates		https://broadinstitute.github.io/picard/
featureCounts	DOI: 10.1093/bioinformatics/btt656	http://bioinf.wehi.edu.au/featureCounts/
Kundaje lab ATAC-seq pipeline		https://github.com/kundajelab/atac-seq-pipeline
Bowtie2 (v.2.2.6)	DOI: 10.1038/nmeth.1923.	http://bowtie-bio.sourceforge.net/bowtie2/index.shtml
Bedtools (v.2.26)	DOI:10.1093/bioinformatics/btq033	https://bedtools.readthedocs.io/en/latest/
MACSv2 (2.1.0)	DOI: 10.1186/gb-2008-9-9-r137	https://github.com/taoliu/MACS
DESeq2 (v.1.32.0)	DOI: 10.1186/s13059-014-0550-8	https://www.bioconductor.org/packages/release/bioc/html/DESeq2.html
Sva (v.3.28.0)	DOI: 10.1093/bioinformatics/bts034	http://bioconductor.org/packages/release/bioc/html/sva.html
edgeR (v.3.22.2)	DOI: 10.1093/bioinformatics/btp616	http://bioconductor.org/packages/release/bioc/html/edgeR.html
RUVseq (v.1.14.0)	DOI: 10.1038/nbt.2931	https://bioconductor.org/packages/release/bioc/html/RUVSeq.html
DAVID	DOI: 10.1186/gb-2007-8-9-r183	https://david.ncifcrf.gov/home.jsp
HOMER	DOI: 10.1016/j.molcel.2010.05.004	http://homer.ucsd.edu/homer/
msCentipede	DOI: 10.1371/journal.pone.0138030	https://github.com/rajanil/msCentipede
JASPAR Core	DOI: 10.1093/nar/gkx1126	http://jaspar.genereg.net/collection/core/
FIMO	DOI: 10.1093/bioinformatics/btr064	http://meme-suite.org/tools/fimo
atactk toolkit (v.0.1.6)		https://github.com/ParkerLab/atactk

ggplot2	https://cran.r-project.org/web/packages/ggplot2/citation.html	https://cran.r-project.org/web/packages/ggplot2/index.html
WGCNA (v.1.64.1)	DOI: 10.1186/1471-2105-9-559	https://cran.r-project.org/web/packages/WGCNA/index.html
AmiGO 2	DOI: 10.1093/bioinformatics/btn615	http://amigo.geneontology.org/amigo
Other		

SUPPLEMENTAL EXPERIMENTAL PROCEDURES

Maintenance and differentiation of hPSC lines

The following hPSC lines were applied in this study: SA121 hESC, SB NEO1.1 hiPSC, SB AD2.1 hiPSC, SB AD3.1 hiPSC, SB AD3.4 hiPSC and NKX6.1-GFP hiPSC line. Details on source of lines can be found in the resource table in the supplemental information. All hPSC were maintained on hESC-qualified matrigel in mTeSR1 with the exception of the NKX6.1-GFP hiPSC line, which was cultured in TeSR1-E8 medium at 37°C, 5% CO^2 as previously described (Perez-Alcantara et al., 2018). Briefly, cells were passaged every 3-4 days or when reaching 90-95% confluence. Medium was aspirated and the cells were washed once with PBS without calcium and magnesium (PBS-/-) and subsequently incubated with TrypLE select (3-5min, 37°C) to achieve a single cell solution. TrypLe select wash quenched with 3-4 volumes of medium with 5µM Rock inhibitor and cells were pelleted (300xG, 5min). Cell number was determined using a NucleoCounter NC-200 (Chemotec, Denmark) and cells were resuspended to the desired concentration in medium with 5µM Rock inhibitor. Cells were seeded in medium with 5uM Rock inhibitor onto tissue culture flasks (Nunclon delta surfaces, Thermo Scientific, #156367 or #156499) pre-coated with hESC-qualified matrigel for maintenance or onto 12-well tissue culture plates (CellBIND surfaces, Corning #3336) for differentiation. For differentiation, cells were seeded at 300.000-400.000 cells/cm^2 and 24h after seeding the cells, medium was aspirated and cells were washed once with PBS with calcium and magnesium (PBS+/+) before exposing the cells to differentiation medium. Stage 1 (definitive endoderm) was shared across all three protocols. Cells were incubated for 1 day with MCDB131 medium containing 0.5% BSA (w/v), 0.1% (v/v) Penicillin-Streptomycin, 1% (v/v) Glutamax, 10mM glucose, 100ng/ml Activin A and 3uM CHIR99021. For day 2, cells were cultured with MCDB131 medium containing 0.5% BSA (w/v), 0.1% (v/v) Penicillin-Streptomycin, 1% (v/v) Glutamax, 10mM glucose, 100ng/ml Activin A and 0.3uM CHIR99021. For day 3 cells were cultured with MCDB131 medium containing 0.5% BSA (w/v), 1.5 g/l $NaHCO_3$, 0.1% (v/v) Penicillin-Streptomycin (PS), 1% (v/v) Glutamax, 10mM glucose and 100ng/ml Activin A. Following induction of definitive endoderm, cells were differentiated with three pancreatic progenitor protocols outlined below. Importantly, differentiations with the three protocols were always performed side-by-side. Medium was prepared freshly for each stage of the differentiation protocol and cells were replenished with medium daily across all protocols and stages. Small molecules and growth factors were aliquoted to appropriate volumes to reduce the number of freeze-thaw cycles (≤3).

**PROTOCOL A adapted from (Rezania et al., 2014)**			
**Stage**	**Medium**	**Compounds**	**Key differences compared to original protocol**
**Stage 2** (2 days)	MCDB131, 0.1% PS, 1% glutamax, 1.5g/l $NaHCO_3$ 10mM glucose, 0.5% BSA, 0.25mM ascorbic acid	50ng/ml KGF (FGF7)	• Differences in maintenance of undifferentiated hPSC compared to original study

**Stage 3**<br>(2 days)	MCDB131, 0.1% PS, 1% glutamax, 2.5g/l $NaHCO_3$ 10mM glucose, 2% BSA, 0.25mM ascorbic acid , 1:200 (v/v) ITS-X	50ng/ml KGF (FGF7), 1µM Retinoic acid, 0.25µM SANT-1, 100nM LDN-193189, 200nM TBP	• CHIR99021 and Activin A used for DE induction compared to MCX-928 and GDF8 in the original publication.<br>• MCDB131 medium used for stage 3-4 compared to custom made medium (BLAR) reported in the original publication.<br>• Differences in vendors used for various supplements and factors.
**Stage 4**<br>(3 days)		2ng/ml KGF (FGF7), 0.1µM Retinoic acid, 0.25µM SANT-1, 200nM LDN-193189, 100nM TBP	

**PROTOCOL B adapted from (Russ et al., 2015)**			
**Stage**	**Medium**	**Compounds**	**Key differences compared to original protocol**
**Stage 2**<br>(2 days)	MCDB131, 0.1% PS, 1% glutamax, 1.5g/l $NaHCO_3$ 10mM glucose, 0.5% BSA, 0.25mM ascorbic acid	50ng/ml KGF (FGF7)	• Differences in maintenance of undifferentiated hPSC compared to original study<br>• Cells differentiated in 2D format compared to suspension culture in the original publication.<br>• Different protocol for DE induction compared to original publication<br>• Differences in medium and FGF7 concentration in stage 2.<br>• Different retinoic acid receptor agonist applied<br>• Differences in vendors used for various supplements and factors.<br>• Differences in length of stage 4.2 compared to original publication
**Stage 3**<br>(2 days)	DMEM(HG) 0.1% PS, 1% B-27 supplement	2µM Retinoic acid	
**Stage 4.1**<br>(1 day)		2µM Retinoic acid, 50ng/ml EGF	
**Stage 4.2**<br>(2 days)		50ng/ml KGF (FGF7), 50ng/ml EGF	

**PROTOCOL C adapted from (Nostro et al., 2015)**			
**Stage**	**Medium**	**Compounds**	**Key differences compared to original protocol**

**Stage 2**<br>(3 days)	DMEM/F12, 0.1% PS, 1% Glutamax, 1% N2 supplement, 1% B27 supplement (- Vitamin A), 0.05% BSA, 0.5mM Ascorbic acid	50ng/ml KGF (FGF7), 50ng/ml Noggin*<br>(**cell line dependent*)	• Differences in maintenance of undifferentiated hPSC compared to original study<br>• Different protocol for DE induction compared to original publication<br>• No Wnt3a in stage 2 of protocol<br>• FGF7 used instead of FGF10 throughout<br>• SANT-1 used compared to KAAD-Cyclopamine in original publication<br>• Differences in vendors used for various supplements and factors.<br>• Activin A during stage 4 of protocol.
**Stage 3**<br>(1 day)	DMEM(HG) 0.1% PS, 1% B-27 supplement (- Vitamin A), 0.5mM Ascorbic acid	2μM Retinoic acid, 50ng/ml KGF (FGF7), 50ng/ml Noggin	
**Stage 4**<br>(3 days)		100ng/ml EGF, 50ng/ml Noggin, 10mM Nicotinamide, 50ng/ml Activin A	

*Omission of Noggin from stage 2 of protocol C resulted in a significantly more efficient differentiation of the SB AD3.1 hiPSC line. The NKX6.1-GFP reporter hiPSC line was derived from the SB AD3.1 hiPSC line and thus, all differentiations of the NKX 6.1-GFP reporter hiPSC line with protocol C was carried out without Noggin during stage 2.

For subsequent endocrine differentiation of the pancreatic progenitors derived from all three protocols, stage 5 and stage 6 of the differentiation protocol outlined below was applied.

**ENDOCRINE DIFFERENTIATION adapted from (Rezania et al., 2014)**		
**Stage**	**Medium**	**Compounds**
**Stage 5**<br>(3 days)	MCDB131, 0.1% PS, 1% glutamax, 1.5g/l $NaHCO_3$ 20mM glucose, 2% BSA, 0.5% ITS-X, 10uM Zinc sulfate	0.05μM Retinoic acid, 0.25μM SANT-1, 100nM LDN-193189, 10μM ALK5i II, 1μM T3, 10μg/ml heparin
**Stage6**<br>(7 days)		100nM LDN-193189, 10μM ALK5i II, 1μM T3, 10μg/ml heparin, 100nM γ-secretase inhibitor XX

Immunofluorescence imaging

Cells were washed once in PBS and fixed directly in the wells of tissue culture plates by incubating with 4% formaldehyde for 20min at room temperature. The fixed cells were subsequently rinsed twice in PBS and then permeabilized for 10min at room temperature in PBS containing 0.5% Triton X-100. Following permeabilization, cells were incubated for 30 min at room temperature with a blocking solution consisting of 0.1 M Tris-HCl pH 7.4, 0.15 M NaCl, and 0.5% Tyramide Signal Amplification (TSA) immunohistochemistry kit blocking reagent. Cells were subsequently incubated with primary antibodies diluted in PBS with 0.1% Triton X-100 overnight at 4°C. Following incubation with primary antibodies, cells were washed three times in PBS and subsequently incubated with secondary antibodies and 4',6-diamidino-2-phenylindole in PBS with 0.1% Triton X-100 for 45min at room temperature. Images were acquired with an inverted fluorescence microscope (Olympus IX-81).

Flow cytometry analysis

Cells were harvested to a single cell suspension using TrypLE select, rinsed once in PBS and stained with Live/Dead cell kit for 10min on ice. Cells were subsequently washed and fixated for 20min at 4°C in 4% formaldehyde. Cells were washed in PBS and incubated for 30min in perm-buffer (PBS +/+ with 0.2% Triton X-100 and 5% Donkey serum) at 4°C. Following permeabilization, cells were pelleted (800 x G, 5min) and resuspended in block-buffer (PBS +/+ with 0.1% Triton X-100 and 5% Donkey serum) containing antibodies. Cells were incubated for 30min at room temperature covered from light and subsequently washed twice in PBS with 1% BSA. Cells were analysed on BD LSR Fortessa cell analyser (BD Biosciences) with 10.000-20.000 events recorded pr. sample.

Fluorescence activated cell sorting
An NKX6.1-T2A-GFP reporter iPSC lines was differentiated side-by-side to the pancreatic progenitor stage using the three differentiation protocols. A wild type iPSC line was also differentiated and used to define the gates for sorting the GFP- and GFP+ fractions. A total of three independent differentiations were performed and from each differentiation both GFP+ and GFP- cells as well as unsorted controls were FACS purified. Briefly, cells were harvested to a single cell suspension using TrypLE select, pelleted and resuspended in the stage 4 medium of the respective protocols (without factors) containing 1µg/ml DAPI solution and directly proceeded for sorting. Cells were sorted using a BD FACSAria Fusion (BD Biosciences) with the 100µM nozzle/20psi configuration. GFP+ and GFP- cells were sorted into Eppendorf tubes containing medium at 4°C. Between 50.000-100.000 cells were sorted for RNA sequencing and 45.000-60.000 cells were sorted for ATAC sequencing.
Real time quantitative PCR (RT-qPCR). RNA was isolated from harvested cells using NucleoSpin RNA/Protein kit. 500ng RNA was converted to cDNA using iScript cDNA Synthesis Kit and adjusted to a final volume of 200ul following reverse transcription. Gene expression was analysed using TagMan Gene Expression Assays (see resource table below). RT-qPCR reactions consisted of 0,5ul Tagman Gene expression assay, 2ul cDNA, 5ul TagMan Gene Expression Master Mix and 2,5ul H_2O. RT-qPCR was performed on a MX3005P qPCR system (Agilent Genomics) using a fast-2-step protocol (first 95°C for 1min, then 40 cycles of 95°C for 10sec, 60°C for 25sec). Expression values were normalized to the average expression of two housekeeping genes (ACTB and HPRT1) and relative expression to unsorted samples was calculated using the ΔΔCt method.

RNA sequencing and processing of sequence data
For RNA sequencing, cells were pelleted immediately following sorting and medium was removed. Cells were harvested and RNA extracted using TRIzol Reagent (ThermoFisher Scientific, Paisley, UK) as per the manufacturer's guidelines. Library preparation and sequencing was performed at the Oxford Genomics Centre (Wellcome Centre for Human Genetics, Oxford, UK) as previously described(van de Bunt et al., 2016). We prepared the Smart-Seq2 paired-end RNA-seq libraries for the 27 samples (3 differentiations x 3 protocols x 3 cell populations: presort, GFP+ and GFP-), and sequenced on Illumina HiSeq4000 to a mean depth of X (+/- X) million 75bp reads pairs per sample. The sequencing quality was assessed with FASTQC (http://www.bioinformatics.babraham.ac.uk/projects/fastqc/) and the raw sequencing reads were mapped to the human genome hg19 using STAR version 2.5.1 with default settings. The GENCODE v19 GTF was applied to guide the spliced alignment. Duplicated alignments were marked with the MarkDuplicates script from the Picard tools v2.1.1 suite (http://broadinstitute.github.io/picard). Gene expression was quantified with featureCounts using the GENCODE v19 GTF file. We assessed the library complexities for all the samples by comparing the number of genes detected at >1TPM with the percentage of total reads mapping within the top 100 expressed genes. We discovered that one of the presort samples from protocol A from differentiation 3 had much lower library complexity (X) than the remaining samples (X), therefore we removed it from further analyses.

Transposition reaction and Purification/ATAC-seq
ATAC-seq sample preparation was done as described previously(Buenrostro et al., 2013). Described shortly, following sorting cells were pelleted and washed in cold PBS. Cell pellets were gently resuspended in cold lysis buffer (10mM Tris-HCL, pH 7.4, 10mM NaCl, 3mM MgCl2, 0.1% IGEPAL CA-630) and immediately pelleted by centrifugation. The supernatant was discarded and the cell pellets were gently resuspended in 50µl transposition reaction mix (25µl 2 x TD buffer, 2.5µl Tn5 Transposase (Illumina), 22.5µl Nuclease free H_2O) and incubated at 37°C for 30min. Following the transposition reaction, the transposased DNA was purified using Qiagens MinElute Kit (Qiagen) according to manufacturer's instructions and eluted in 10µl elution buffer (10mM Tris buffer, pH 8). Eluted DNA was PCR amplified using Nextera PCR primers (Illumina) and NEBNext high fidelity PCR master mix (New England Biolabs) with the following cycling 1) 72°C, 5 min, 2) 98°C, 30 sec , 3) 98°C, 10 sec, 4) 63°C, 30 sec, 5) 72°C, 1 min, Repeating steps 3-5, 9-11x. PCR product was purified using Qiagens MinElute Kit (Qiagen) according to manufacturer's instructions and eluted in 20µl elution buffer (10mM Tris buffer, pH 8). Quality of final

library was assessed using Tapestation (agilent). Libraries were sequenced at the High-Throughput Genomics core at the Wellcome Centre for Human Genetics, University of Oxford, UK. Samples were sequenced as multiplexed pools on HiSeq4000 (Illumina) using 75-bp paired-end sequencing.
The ATAC-seq libraries for the 27 samples (3 differentiations x 3 protocols x 3 cell populations: presort, GFP+ and GFP-) were sequenced on Illumina HiSeq4000 to a mean depth of X (+/- X) million 75bp reads pairs per sample. The raw sequencing reads were processed with the Kundaje lab ATAC-seq pipeline (https://github.com/kundajelab/atac-seq-pipeline). The pipeline detects and trims the sequencing adaptors, maps the reads to the human reference genome build hg19 using Bowtie2 version 2.2.6. The duplicated alignments are marked with MarkDuplicates script from the Picard tolos suite, converts the BAM files to tagAlign format using bedtools versión 2.26 and calls the signal peaks using MACSv2 (2.1.0).

Principal component analysis
The global similarities between the samples for both the RNA-seq and ATAC-seq datasets were assessed through principal component analysis (PCA). The gene count tables were transformed with variance stabilizing transformation (vst) in the DESeq2 R package (v.1.32.0)(Love et al., 2014). The batch effects of the two multiplexing pools introduced for sequencing in the ATAC-seq dataset were corrected using the sva R package (v.3.28.0)(Leek et al., 2012). The PCA analysis was performed using the plotMDS function from the edgeR R package (v.3.22.2)(Robinson et al., 2010), using the setting gene.selection="common", and focusing on the top 500 most variable genes. For the PCA analysis of RNA-seq samples from this study together with the RNA-seq dataset from a study of human fetal pancreas populations (Ramond 2018), presented in Fig.3D, we merged the raw gene count tables for the two studies, performed the variance stabilizing transformation in DESeq2 on the merged count table, followed by sva batch correction for the effect of the study of origin.

Defining the common PP signature
To derive common transcriptomic and epigenomic signatures of pancreatic progenitors generated in this study with three differentiation protocols, we compared the data to the equivalent omics profiles of all stages of hPSC differentiation towards beta-line cells (Perez Alcantara 2018). To facilitate the comparison we applied the RUVs normalization implemented in the RUVseq (v.1.14.0) R package(Risso et al., 2014). We first applied the upper-quartile normalization to account for differences in library sizes, followed by the RUVs approach to estimate and remove the factors of unwanted variation. For that purpose we treated the presorted samples generated with protocol A as replicates of the control lines samples from the pancreatic endoderm stage (also generated with protocol A). After the RUVs normalization, we generated the normalized count tables for all the samples and used them to calculate CPM (counts-per-million) expression values using the cpm() function from the edgeR R package. We then calculated mean CPM values for each sample group (GFP cell population from each protocol, and all differentiation stages). We then defined pancreatic progenitor signatures within the GFP+ cell populations for each protocol separately; and then we used the intersection of the three individual protocol-specific signatures to define the common cross-protocol pancreatic progenitor signature. To define the sets of genes forming a pancreatic progenitor signature for each protocol, we required that the genes were expressed at >1 TPM in the GFP+ population from the given protocol, demonstrated stage selectivity (CV>1), as well as specificity to the pancreatic endoderm (Z-score >1) when evaluated together with the remaining stages. We calculated similar PP signatures in the presort, and GFP- populations as well. The PP signatures were derived in the same manner for both the RNA-seq and ATAC-seq datasets. Functional enrichments for the PP signature genes were calculated with DAVID Gene Functional Classification tool(Huang et al., 2007); raw enrichment p-values for selected Gene Ontology categories were then plotted as -log10(p-value) barplots in Fig.3C. Enrichment of known transcription factor binding motifs was calculated with HOMER(Heinz et al., 2010) using the default settings.

Footprinting analysis
msCentipede(Raj et al., 2015) was used to call sites bound by transcription factors. First, transcription factor binding motifs were called genome-wide using position weight matrices from JASPAR Core (non-redundant) 2018 (Khan et al., 2018) using FIMO (version 4.11.2, (Grant et al., 2011)) with default parameters. The model parameters were learned per experiment and GFP status using genome-wide called motifs and replicate bam files. Transcription factor binding motifs were called in each replicate peak file (for high quality peaks with q-value $< 10^{-6}$; per experiment and GFP status), and subsequently the replicates were merged and used as input for the inference step in msCentipede. Sites with the following scores were considered bound: LogPosOdds > 2, MultLikeRatio>1 and NegBinLikeRatio>1; and the mean counts per site were calculated using make_cut_site function in the atactk toolkit (v. 0.1.6)(https://github.com/ParkerLab/atactk) and plotted using ggplot2 (Wickham, 2016) in R (v. 3.4.3).

DEGs/DOCS
Testing for differential expressed genes (DEGs) and differentially open chromatin sites (DOCS) was performed with DESeq2 R package (v…). We compared data from each differentiation protocol to the data generated with the two other protocols, for each cell population (presort, GFP+ and GFP-) separately. We also compared the data between the GFP+ and GFP- cell populations for each protocol separately. In both RNA-seq and ATAC-seq analyses we adjusted for the effects of the differentiations, and in the ATAC-seq additionally also for the effects of the sequencing multiplex pool. We considered all genes and open chromatic sites with FDR-adjusted p-values<0.05 as
significantly differentially expressed.

WGCNA
We performed weighted gene co-expression analysis (WGCNA)(Langfelder and Horvath, 2008) on both the vst-transformed RNA-seq and ATAC-seq datasets. The RNA-seq analysis was performed on 15,555 protein-coding genes expressed at >=1 TPM in at least 3 samples. The ATAC-seq analysis was limited to 19,739 open chromatin peaks, with >=10 sequencing reads in at least 3 samples, and a coefficient of variation >0.5. Using the criteria of best fit to the scale free topology model we selected power of 6 for soft thresholding of RNA-seq data, and power of 3 for the ATAC-seq dataset, and required a minimum module size of 50 for both analyses. We identified 20 co-expressed gene modules in the RNA-seq dataset, and 11 modules of correlated open chromatin sites. The dimensionality reduction of these large datasets is achieved through assignment of a single eigengene value for each modules in each sample. The eigengene value represents the first principal component value for the given module genes or open chromatin regions. The module eigengene values for the samples with both RNA-seq and ATAC-seq data can then be correlated to derive pairs of highly correlated modules. These are likely to correspond to open chromatin at regulatory elements controlling the gene expression of the correlated genes.

Gene signatures and Gene Ontology functional enrichment testing
For each of the 20 co-expressed gene modules we tested enrichment in selected gene sets using the hypergeometric distribution. We investigated enrichments of the *in vitro* pancreatic progenitor signatures derived in this study, as
described in an earlier section, as well as the 500 gene PP signature derived through comparison of *in vitro* and *in vivo* multipotent pancreatic progenitors from a previous study by Cebola *et al*. 2015. We then tested whether any of the gene modules were enriched in selected developmental Gene Ontology terms: GO:0031016 pancreas development, GO:0001889 liver development , GO:0048565 digestive tract development and GO:0060575 intestinal
epithelial cell differentiation. Genes belonging to these Gene Ontology categories were identified using AmiGO 2 Ontology search function(Carbon et al., 2009), accessed on 10th April 2018. We tested whether any of the gene modules might represent cells with likely intestinal fate through enrichment of previously reported genes differentially and highly expressed in intestinal stem cells derived from duodenum, jejunum and ileum(Wang et al., 2015). We also performed differential gene expression to identify significantly up-regulated biomarkers of dorsal pancreas, hepatobiliary primordium and hepatic cords from laser capture dissection in human embryos(Jennings et
al., 2017). We then tested enrichment for these sets of markers with log2FC>0 and FDR q-value<0.05 in the co-expressed gene modules using the hypergeometric distribution. Enrichment of known transcription factor binding motifs within co-open chromatin modules was calculated with HOMER using the default settings(Heinz et al., 2010).

SUPPLEMENTAL REFERENCES

Buenrostro, J. D., Giresi, P. G., Zaba, L. C., Chang, H. Y. & Greenleaf, W. J. (2013). Transposition of native chromatin for fast and sensitive epigenomic profiling of open chromatin, DNA-binding proteins and nucleosome position. Nat Methods, **10,** 1213-8.

Carbon, S., Ireland, A., Mungall, C. J., Shu, S., Marshall, B., Lewis, S., Ami, G. O. H. & Web Presence Working, G. (2009). AmiGO: online access to ontology and annotation data. Bioinformatics, **25,** 288-9.

Grant, C. E., Bailey, T. L. & Noble, W. S. (2011). FIMO: scanning for occurrences of a given motif. Bioinformatics, **27,** 1017-8.

Heinz, S., Benner, C., Spann, N., Bertolino, E., Lin, Y. C., Laslo, P., Cheng, J. X., Murre, C., Singh, H. & Glass, C. K. (2010). Simple combinations of lineage-determining transcription factors prime cis-regulatory elements required for macrophage and B cell identities. Mol Cell, **38,** 576-89.

Huang, D. W., Sherman, B. T., Tan, Q., Collins, J. R., Alvord, W. G., Roayaei, J., Stephens, R., Baseler, M.

W., Lane, H. C. & Lempicki, R. A. (2007). The DAVID Gene Functional Classification Tool: a novel biological module-centric algorithm to functionally analyze large gene lists. Genome Biol, 8, R183.
Jennings, R. E., Berry, A. A., Gerrard, D. T., Wearne, S. J., Strutt, J., Withey, S., Chhatriwala, M., Piper Hanley, K., Vallier, L., Bobola, N., et al. (2017). Laser Capture and Deep Sequencing Reveals the Transcriptomic Programmes Regulating the Onset of Pancreas and Liver Differentiation in Human Embryos. Stem Cell Reports, 9, 1387-1394.
Khan, A., Fornes, O., Stigliani, A., Gheorghe, M., Castro-Mondragon, J. A., van der Lee, R., Bessy, A., Cheneby, J., Kulkarni, S. R., Tan, G., et al. (2018). JASPAR 2018: update of the open-access database of transcription factor binding profiles and its web framework. Nucleic Acids Res, 46, D260-D266.
Langfelder, P. & Horvath, S. (2008). WGCNA: an R package for weighted correlation network analysis. BMC Bioinformatics, 9, 559.
Leek, J. T., Johnson, W. E., Parker, H. S., Jaffe, A. E. & Storey, J. D. (2012). The sva package for removing batch effects and other unwanted variation in high-throughput experiments. Bioinformatics, 28, 882-3.
Love, M. I., Huber, W. & Anders, S. (2014). Moderated estimation of fold change and dispersion for RNA-seq data with DESeq2. Genome Biol, 15, 550.
Nostro, M. C., Sarangi, F., Yang, C., Holland, A., Elefanty, A. G., Stanley, E. G., Greiner, D. L. & Keller, G. (2015). Efficient generation of NKX6-1+ pancreatic progenitors from multiple human pluripotent stem cell lines. Stem Cell Reports, 4, 591-604.
Perez-Alcantara, M., Honore, C., Wesolowska-Andersen, A., Gloyn, A. L., McCarthy, M. I., Hansson, M., Beer, N. L. & van de Bunt, M. (2018). Patterns of differential gene expression in a cellular model of human islet development, and relationship to type 2 diabetes predisposition. Diabetologia, 61, 1614-1622.
Raj, A., Shim, H., Gilad, Y., Pritchard, J. K. & Stephens, M. (2015). msCentipede: Modeling Heterogeneity across Genomic Sites and Replicates Improves Accuracy in the Inference of Transcription Factor Binding. PLoS One, 10, e0138030.
Rezania, A., Bruin, J. E., Arora, P., Rubin, A., Batushansky, I., Asadi, A., O'Dwyer, S., Quiskamp, N., Mojibian, M., Albrecht, T., et al. (2014). Reversal of diabetes with insulin-producing cells derived in vitro from human pluripotent stem cells. Nat. Biotechnol, 32, 1121-1133.
Risso, D., Ngai, J., Speed, T. P. & Dudoit, S. (2014). Normalization of RNA-seq data using factor analysis of control genes or samples. Nat Biotechnol, 32, 896-902.
Robinson, M. D., McCarthy, D. J. & Smyth, G. K. (2010). edgeR: a Bioconductor package for differential expression analysis of digital gene expression data. Bioinformatics, 26, 139-40.
Russ, H. A., Parent, A. V., Ringler, J. J., Hennings, T. G., Nair, G. G., Shveygert, M., Guo, T., Puri, S., Haataja, L., Cirulli, V., et al. (2015). Controlled induction of human pancreatic progenitors produces functional beta-like cells in vitro. EMBO J, 34, 1759-72.
van de Bunt, M., Lako, M., Barrett, A., Gloyn, A. L., Hansson, M., McCarthy, M. I., Beer, N. L. & Honore, C. (2016). Insights into islet development and biology through characterization of a human iPSC-derived endocrine pancreas model. Islets, 8, 83-95.
Wang, X., Yamamoto, Y., Wilson, L. H., Zhang, T., Howitt, B. E., Farrow, M. A., Kern, F., Ning, G., Hong, Y., Khor, C. C., et al. (2015). Cloning and variation of ground state intestinal stem cells. Nature, 522, 173-8.
Wickham, H. (2016). ggplot2: Elegant Graphics for Data Analysis. Springer-Verlag New York.
