# Supplementary material for: Analysis of Differentiation Protocols Defines a Common Pancreatic Progenitor Molecular Signature and Guides Refinement of Endocrine Differentiation
Source: Stem Cell Reports. 2019 Dec 26;14(1):138–53. doi: 10.1016/j.stemcr.2019.11.010 (PMC6962645; doi:10.1016/j.stemcr.2019.11.010)
Supplement: Document S2. Article plus Supplemental Information [file mmc4.pdf]

Stem Cell Reports
Resource
OPEN ACCESS

Analysis of Differentiation Protocols Defines a Common Pancreatic Progenitor Molecular Signature and Guides Refinement of Endocrine Differentiation

Agata Wesolowska-Andersen,[1] Rikke Rejnholdt Jensen,[5] Marta Pérez Alcántara,[1] Nicola L. Beer,[2] Claire Duff,[2] Vibe Nylander,[2] Matthew Gosden,[4] Lorna Witty,[1] Rory Bowden,[1] Mark I. McCarthy,[1,2,3] Mattias Hansson,[5] Anna L. Gloyn,[1,2,3] and Christian Honore[5,*]
[1]Wellcome Centre Human Genetics, University of Oxford, OX3 7BN Oxford, UK
[2]Oxford Centre for Diabetes, Endocrinology and Metabolism, University of Oxford, OX3 7LE Oxford, UK
[3]NIHR Oxford Biomedical Research Centre, Churchill Hospital, OX3 7LE Oxford, UK
[4]The MRC Weatherall Institute of Molecular Medicine, University of Oxford, OX3 9DS Oxford, UK
[5]Stem Cell R&D, Novo Nordisk A/S, 2760 Måløv, Denmark
*Correspondence: clfh@novonordisk.com
https://doi.org/10.1016/j.stemcr.2019.11.010

SUMMARY

Several distinct differentiation protocols for deriving pancreatic progenitors (PPs) from human pluripotent stem cells have been described, but it remains to be shown how similar the PPs are across protocols and how well they resemble their *in vivo* counterparts. Here, we evaluated three differentiation protocols, performed RNA and assay for transposase-accessible chromatin using sequencing on isolated PPs derived with these, and compared them with fetal human pancreas populations. This enabled us to define a shared transcriptional and epigenomic signature of the PPs, including several genes not previously implicated in pancreas development. Furthermore, we identified a significant and previously unappreciated cross-protocol variation of the PPs through multi-omics analysis and demonstrate how such information can be applied to refine differentiation protocols for derivation of insulin-producing beta-like cells. Together, our study highlights the importance of a detailed characterization of defined cell populations derived from distinct differentiation protocols and provides a valuable resource for exploring human pancreatic development.

INTRODUCTION

Human pluripotent stem cells (hPSCs) have tremendous potential for modeling human diseases *in vitro* as well as for regenerative medicine in degenerative diseases. However, the realization of both these applications of hPSCs is dependent on the ability to derive the relevant cell lineages from hPSCs by directed differentiation.

In the context of pancreas development, studies in mice have demonstrated that exocrine, ductal, and endocrine lineages all derive from multipotent pancreatic progenitor (PP) cells, defined by co-expression of several transcription factors (TFs), including PDX1, NKX6.1, PTF1a, and SOX9 (Larsen and Grapin-Botton, 2017). Despite noteworthy differences in human pancreas development compared with mouse (Jennings et al., 2015; Nair and Hebrok, 2015), human PPs express a similar core network of TFs, including PDX1 and NKX6.1 (Petersen et al., 2018). When transplanted into immunocompromised mice, the hPSC-derived PPs are able to give rise to all lineages of the pancreas (Kelly et al., 2011; Kroon et al., 2008; Rezania et al., 2012, 2013), supporting their similarity to multipotent PPs observed during development. Knowledge gained from rodent models of pancreas development facilitated many of the advancements in differentiation protocols. For example, retinoic acid and fibroblast growth factor signaling are indispensable for the specification and expansion of PPs during development (Bhushan et al., 2001; Molotkov et al., 2005), and the majority of current differentiation protocols include agonists of these signaling pathways. However, there are also notable differences in protocols reported to differentiate hPSCs to PPs. For example, bone morphogenetic protein (BMP) signaling has been shown to promote a liver fate choice rather than pancreas development (Wandzioch and Zaret, 2009), and thus several protocols include BMP inhibitors during differentiation. However, a recent report argued for the exclusion of BMP inhibitors, since these were shown to promote a premature endocrine differentiation at the expense of PDX1/NKX6.1-positive PPs (Russ et al., 2015). There is also no consensus on inclusion of other pathway modulators, such as epidermal growth factor (EGF) or protein kinase C (PKC) agonists, in the differentiation protocols (Nostro et al., 2015; Rezania et al., 2014; Russ et al., 2015).

As hPSC-derived PPs are often defined by co-expression of a limited set of genes (e.g., *PDX1* and *NXK6.1*), it remains unclear how similar the PP cells derived from various differentiation protocols are and how well they represent embryonic development and subsequent development of more mature cell types of the pancreatic islet.

 © 2019 The Author(s).
This is an open access article under the CC BY license (http://creativecommons.org/licenses/by/4.0/).

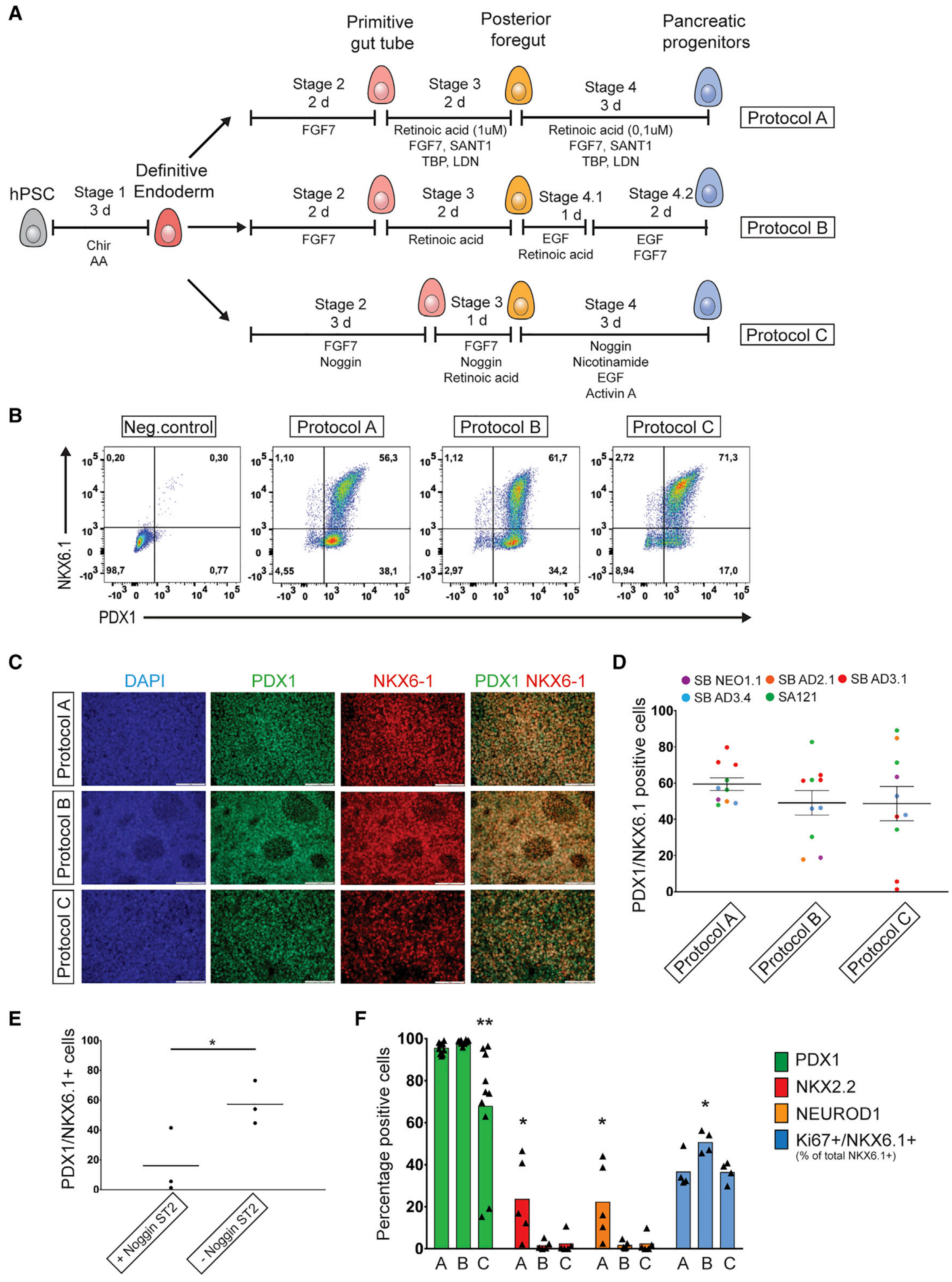

Figure 1. Derivation of PPs from hPSCs Using Multiple Differentiation Protocols

(A) Schematic outline of the three PP protocols applied.

(B) Representative examples of flow cytometry pseudo color dot plots of PPs from the three protocols stained for PDX1 and NKX6.1. Negative control is definitive endoderm cells.

(legend continued on next page)

To address these questions, we performed a detailed characterization of PPs derived using three differentiation protocols adapted from recent publications (Nostro et al., 2015; Rezania et al., 2014; Russ et al., 2015), although with several differences compared with the original publications. We were able to recapitulate several aspects of the reported protocols and achieved efficient differentiation toward PPs across all three protocols. Isolation of PPs allowed us to define their comprehensive gene expression and chromatin accessibility signatures shared across all protocols, which shed light on new endocrine pancreas development biology and will serve as a valuable resource for future studies in disease modeling and cellular replacement therapies. We also highlight several notable differences in the omics profiles of the PPs derived with the different protocols, which translate to differences in their ability to differentiate further toward the endocrine lineage. Finally, we illustrate the utility of these datasets to further optimize various stages of the differentiation protocols to improve the endocrine differentiation and the derivation of beta-like cells from hPSCs.

RESULTS

Efficient Derivation of PPs from Multiple hPSC Lines Using Three Distinct Differentiation Protocols

To compare PP differentiation protocols, we first applied a common protocol for efficient derivation of definitive endoderm (DE) from hPSCs (Rezania et al., 2014; Perez-Alcantara et al., 2018) (Figures S1A and S1B). DE was differentiated toward PPs using protocols outlined in Figure 1A. The three differentiation protocols (A, B, and C) were adapted from previously established protocols (Nostro et al., 2015; Rezania et al., 2014; Russ et al., 2015), but with several differences compared with the original publications (protocol details outlined in Supplemental Experimental Procedures, including key differences from the published protocols). Differentiation efficiency was assessed by flow cytometer and immunofluorescence imaging analysis of expression of the two key PP TFs: PDX1 and NKX6.1 (Figures 1B–1D). Across five hPSC lines we observed an average differentiation efficiency of 59%, 49%, and 48% PDX1/NKX6.1-positive cells derived using protocols A, B, and C, respectively (Figure 1D). A notable variation in the efficiency of PDX1/NKX6.1-positive cells was observed across differentiations (Figure 1D), which may be attributed to the different abilities of individual hPSC lines to differentiate to PPs across these protocols. The hPSC line SB AD3.1 displayed poor differentiation efficiency using protocol C (Figures 1D and S1C), but omission of Noggin during stage 2 of protocol C led to a significant improvement of its differentiation (Figure 1E). Extending this observation, we evaluated the effect of Noggin during stage 2 of all three protocols on the PP differentiation efficiency; however, we found that the effect was not consistent across either cell lines or differentiation protocols (Figure S1C).

A large proportion of the PDX1/NKX6.1-positive cells across the three protocols were proliferating, as demonstrated by the expression of the proliferation marker Ki67 (Figure 1F). Protocols B and C were originally reported to generate a high percentage of PPs, while limiting the commitment to the endocrine lineage (Russ et al., 2015; Nostro et al., 2015). In agreement with this, we observed very few cells expressing the endocrine markers NEUROD1 and NKX2.2 in protocol B and C, whereas protocol A gave rise to a significantly higher number of endocrine cells at this stage of the protocol (Figure 1F). Most of the endocrine progenitors generated were NKX6.1 negative (Figures S1D and S1E), in agreement with previous studies (Nostro et al., 2015; Petersen et al., 2017; Russ et al., 2015). In summary, we are able to recapitulate key aspects of the original reports and we show that all three differentiation protocols efficiently derive PDX1/NKX6.1-positive PPs across multiple hPSC lines.

Global Gene Expression and Chromatin Accessibility Analysis of hPSC-Derived PPs

We next aimed to characterize the PPs derived from the three differentiation protocols in more detail. We differentiated an NKX6.1-GFP reporter human induced PSC (hiPSC) line (Gupta et al., 2018) to PPs using the three

(C) Immunofluorescence images of PPs stained for PDX1 and NKX6.1. Scale bar, 100 μm.
(D) Quantification of PDX1 and NKX6.1 co-expressing cells based on the flow cytometric analysis shown in (B). Graph shows a scatterplot of the mean ± SEM of five individual hPSC lines. Dots are color coded according to individual cell lines (details in Figure S1C). n = 10 independent experiments.
(E) Percentage of PDX1 and NKX6.1 co-expressing cells from the SB AD3.1 hiPSC line differentiated with protocol C with or without 50 ng/mL Noggin included during stage two; n = 3 independent experiments; *$p < 0.05$, paired t test.
(F) Quantification of PDX1, NKX2.2, NEUROD1, and percentage NKX6.1+ cells co-expressing Ki67. Bars show means and dots represent individual differentiations. One-way ANOVA with the Tukey test for multiple comparisons, *$p < 0.05$, **$p < 0.01$, different from the two other groups. PDX1, n = 10 independent experiments, same hPSC lines as in (D). NKX2.2 and NEUROD1, n = 5 independent experiments, one for each of the following hPSC lines: SA121 hESC, SB NEO1.1 hiPSC, SB AD2.1 hiPSC, SB AD3.1 hiPSC, SB AD3.4 hiPSC. Ki67, n = 4 independent experiments, three using SB AD3.1 hiPSC and one using SA121 hESC.

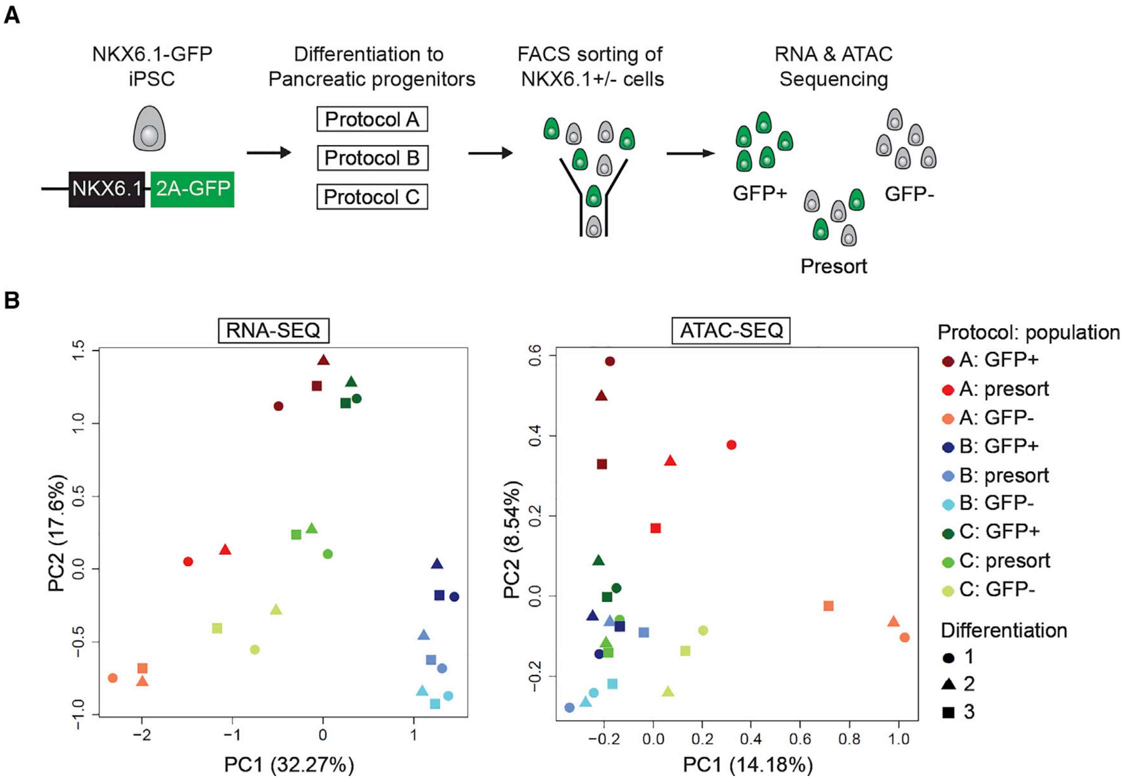

Figure 2. Global Gene Expression and Chromatin Accessibility Analysis of FACS-Isolated PP Populations

(A) Schematic showing the experimental setup. NKX6.1-GFP hiPSCs were differentiated side by side using all three protocols and GFP+ and GFP− cells as well as unsorted cells were collected following FACS for RNA and ATAC sequencing. Cells were collected from three independent differentiations of all three protocols.

(B) Principal component analysis (PCA) of RNA-seq (left) and ATAC-seq data (right). Legend applies to both PCA plots.

differentiation protocols and isolated both GFP+ and GFP− cells using fluorescence-activated cell sorting (FACS) (Figure 2A). Post-sort analysis of live cells demonstrated isolation of highly pure GFP+ and GFP− populations across all three protocols (Figures S2A and S2B) and efficient differentiation of the reporter line to PPs by all protocols was confirmed by flow cytometry analysis of GFP and NKX6.1 expression (Figures S2C and S2D). The isolated populations were processed for genome-wide transcriptome and chromatin accessibility analysis by RNA sequencing (RNA-seq) and assay for transposase-accessible chromatin (ATAC) using sequencing (ATAC-seq), respectively. Global principal component analysis (PCA) of the datasets (Figure 2B) revealed that samples clustered by protocol and by sorted cell population. Samples generated with protocol A were the most distinct and showed the largest separation of the GFP-sorted cell populations in both datasets. For all three protocols, we observed the presort samples clustering in between the sorted populations, as expected. In addition, we confirmed that expression of *NKX6.1* and *PDX1* was enriched in the GFP+ population for all protocols (Figures S2E and S2F).

Cross-Protocol PP Gene Expression and Chromatin Accessibility Signature

We then explored the similarities of the omics profiles of the PPs derived with the different protocols. For that purpose, we compared the samples generated in this study with gene expression and open chromatin profiles of our previously published differentiation model (Perez-Alcantara et al., 2018) across all seven stages of hPSC differentiation toward beta-like cells. The PCA analysis for both the RNA-seq (Figure 3A) and ATAC-seq (Figure 3E) datasets revealed that all the samples characterized in this study clustered, as expected, together with the pancreatic endoderm stage samples from the control dataset. Interestingly, the GFP− cells generated with protocol A clustered out close to the cells from the subsequent differentiation stage, the endocrine progenitors. This was most pronounced in the RNA-seq data, but also apparent in the ATAC-seq data, and is in line with the higher percentage of endocrine cells observed in the NKX6.1− cells with this protocol (Figures 1F, S1D, S1E, S2C, and S2D).

We then sought to derive a PP gene expression signature common to all three protocols by comparing the

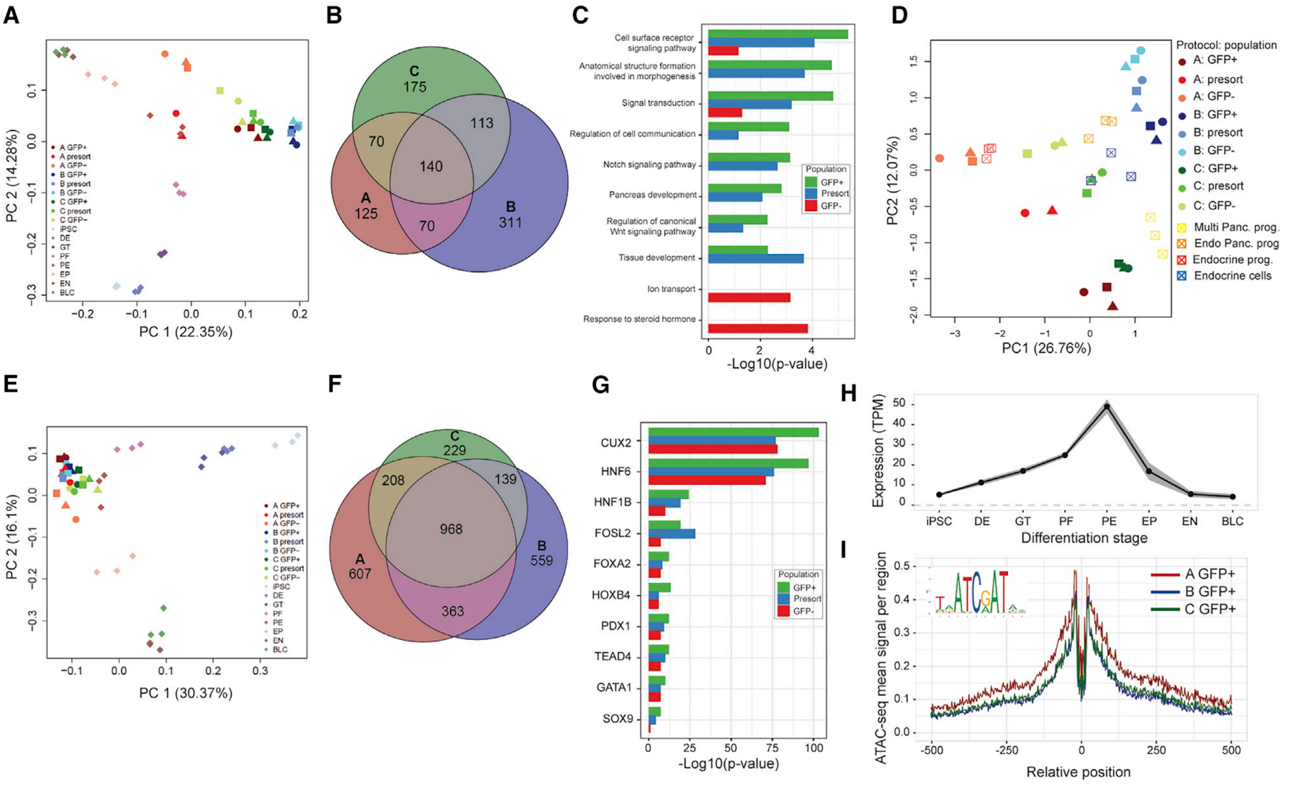

Figure 3. Common Transcriptomic and Epigenomic PP Signatures across Three Differentiation Protocols

(A) PCA of RNA-seq samples together with data collected at all stages of the hPSC differentiation toward beta-like cells generated with protocol A (Perez-Alcantara et al., 2018).

(B) Venn diagram of the RNA-seq GFP+ PP signatures generated separately for each of the protocols by comparison with cells at the other differentiation stages.

(C) Selected gene ontology enrichment of the common PP signature genes across the three protocols; shown separately for signatures derived with presort cells (in blue), GFP+ (in green), and GFP− (in red) cell populations. The length of the bar represents −log10 of the enrichment p value.

(D) PCA of RNA-seq samples from this study, together with transcriptomes of fetal pancreas cell subpopulations (Ramond et al., 2018).

(E) PCA of ATAC-seq samples from this study, together with data collected at all stages of the hPSC differentiation toward beta-like cells, generated with protocol A (Perez-Alcantara et al., 2018).

(F) Venn diagram of the ATAC-seq GFP+ PP signatures generated separately for each of the protocols by comparison with cells at the other differentiation stages.

(G) Enrichment of selected TFs within the common PP signature open chromatin peaks across the three protocols; shown separately for signatures derived with presort cells (in blue), GFP+ (in green), and GFP− (in red) cell populations.

(H) Mean expression of CUX2 gene across all stages of hPSC differentiation toward beta-like cells. Shaded gray area indicates ± SEM (Perez-Alcantara et al., 2018).

(I) Footprinting analysis of CUX2 binding motifs within open chromatin peaks of the GFP+ populations generated with the three differentiation protocols.

BLC, beta-like cells; EN, endocrine cells; ; EP, endocrine progenitors; GT, gut tube; PE, pancreatic endoderm/progenitors; PF, posterior foregut.

transcriptome profiles of the GFP+ cell populations from each of the three protocols separately with the gene expression profiles of the remaining differentiation stages. This resulted in 405, 634, and 498 genes defining the PPs in protocols A, B, and C, respectively, and we next defined the 140-gene common cross-protocol signature by intersecting the three individual protocol-specific signatures (Figure 3B). In a similar manner, we defined a 112-gene PP signature within the presorted cells, and a 129-gene signature within the GFP− cell populations (Table S1). Functional enrichment analysis revealed that several genes forming the signature were involved in cell surface receptor signaling pathways, including Notch and Wnt pathways, and included several genes previously implicated in pancreas

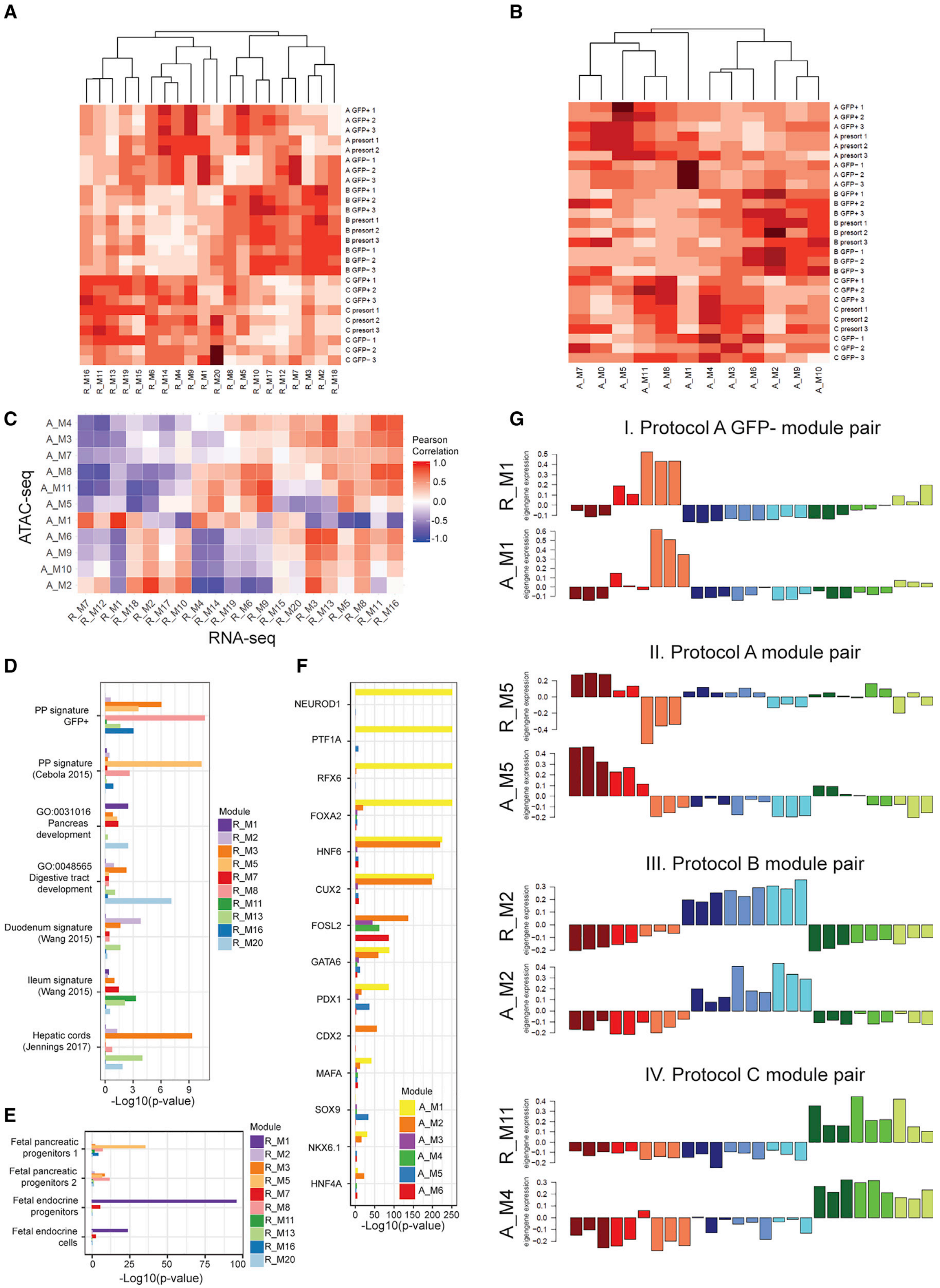

(legend on next page)

development (Figure 3C, Table S1). In addition, for an unbiased comparison with the transcriptomes of PPs *in vivo*, we compared the expression of the samples generated in this study with recently published transcriptomes of human fetal pancreas subpopulations (Ramond et al., 2018) (Figure 3D). We observed that the GFP− population derived with protocol A resembled closely the fetal endocrine progenitor population, in agreement with the high proportion of endocrine differentiation observed with this protocol. Interestingly, the GFP+ populations of protocols A and C clustered close to the fetal multipotent PPs, suggesting a high level of similarity between these *in vitro*-generated PP and human fetal multipotent PP cells. The other two fetal pancreatic cell subpopulations, comprising endocrine-biased PP and endocrine cells, clustered in between the presorted cell populations generated by protocols B and C.

In a similar manner, we derived the open chromatin PP signature, comprising a total of 968 ATAC-seq peaks within the GFP+ population (Figure 3F, Table S1). These open chromatin regions were significantly enriched in binding sites of CUX2 and HNF6 as well as several other TFs with previously established functions in pancreatic development and links to monogenic diabetes, including HNF1B, FOXA2, and PDX1 (Figure 3G). CUX2 was the most significantly enriched TF; however, it has to our knowledge not been implicated in pancreatic development. *CUX2* was expressed in the PP cell populations derived with all three protocols, and its expression was highest at this stage of differentiation in the control lines (Figure 3H). We have subsequently performed a footprinting analysis of the ATAC-seq peaks containing the CUX2 binding motif (Figure 3I) and observed a clear dip in the sequencing coverage around the investigated CUX2 sites, supporting the hypothesis that this TF is bound within the open chromatin at this stage.

Correlated Modules of Gene Expression and Open Chromatin Highlight Between-Protocol Differences

We next sought to determine the differences in the transcriptional and chromatin accessibility profiles of the PPs derived with the different protocols. We applied weighted gene co-expression network analysis (WGCNA) (Langfelder and Horvath, 2008) to our datasets, and conducted further analyses on the dimensionality reduced sets of modules combining correlated patterns of gene expression and open chromatin. We defined 20 modules of co-expressed genes from the RNA-seq data (Figure 4A, Table S2), as well as 11 modules of co-open chromatin sites from the ATAC-seq data (Figure 4B, Table S2). We then identified highly correlated pairs of RNA-seq and ATAC-seq modules, likely representing the modules of active open chromatin regulating the correlated genes' expression (Figure 4C). For each of the gene expression modules, we performed hypergeometric testing of enrichment for significant overlap with gene signatures of PPs from this and previous studies (Cebola et al., 2015), genes involved in selected developmental processes (Figure 4D, Table S2), as well as gene signatures of fetal pancreas cell subpopulations (Ramond et al., 2018) (Figure 4E, Table S2). For each of the open chromatin modules, we identified TF binding motifs enriched within the module's peaks (Figure 4F). We highlight a few of the most interesting correlated RNA-ATAC gene module pairs in Figure 4G.

The most distinct gene expression and correlated open chromatin pattern observed was for RNA module R_M1 and ATAC module A_M1 (Figure 4GI). Both these modules had the highest eigengene values, corresponding to the first principal component and summarizing the overall pattern of gene expression for all genes in the module, in the GFP− cell population from protocol A. The R_M1 genes were enriched in genes involved in pancreas development,

Figure 4. Protocol-Specific Differences in Transcriptomic and Epigenomic Profiles of PPs Generated with Three Differentiation Protocols

(A) Heatmap of RNA-seq co-expressed gene modules eigengenes across all RNA-seq samples. Higher red color intensities indicate higher eigengene values.

(B) Heatmap of ATAC-seq co-open chromatin modules eigengenes across all ATAC-seq samples. Higher red color intensities indicate higher eigengene values.

(C) Pairwise Pearson correlation heatmap of module eigengene values for RNA-seq and ATAC-seq modules.

(D) Hypergeometric enrichment p values for gene signatures of PP signatures, selected developmental gene ontologies, and gene signatures of selected intestinal and hepatic tissue/cell types. Only modules with enrichment p values <0.01 for any of the selected categories in (D) and (E) are plotted. Full list is available in Table S1.

(E) Hypergeometric enrichment p values for transcriptomic signatures of *in vivo* fetal pancreatic cell subpopulations from Ramond et al. (2018). Only modules with enrichment p values <0.01 for any of the selected categories in (D) and (E) are plotted.

(F) Enrichment p values of selected known TF binding sites within modules of co-open chromatin. Only modules with enrichment p values <1e−20 for any of the selected TFs are plotted.

(G) Selected highly correlated pairs of RNA-seq and ATAC-seq modules eigengenes. The height of the bars represents the eigengene values for each sample in the presented module. Bars are colored by each protocol/cell population, as outlined in figure legends for PCA plots in Figure 2B, and in other subsequent figures in this paper. I, protocol A GFP− module pair; II, protocol A GFP+ module pair; III, protocol B module pair; IV, protocol C module pair.

and very closely resembled the *in vivo* fetal endocrine progenitors, which was in line with the previous PCA analysis (Figure 3D). The A_M1 peaks were significantly enriched in binding sites of several TFs with known roles in pancreatic and endocrine development, including NEUROD1, PTF1A, RFX6, and FOXA2. This evidence corroborates that the GFP− population of protocol A represents a population of early endocrine progenitors, which is absent in the other two protocols.

Genes preferentially expressed in the GFP+ population of protocol A, and correlated open chromatin peaks, were captured in modules R_M5 and A_M5 (Figure 4GII). Importantly, these genes were also expressed in the GFP+ cell populations generated with protocols B and C, albeit at lower depth. The R_M5 genes were enriched in PP signatures derived earlier in this study, as well as in a previous study by Cebola and colleagues (Cebola et al., 2015), and were enriched in the transcriptomic signature of the *in vivo* multipotent PPs (Ramond et al., 2018). Open chromatin in module A_M5 was enriched in binding motifs of PDX1, SOX9, and PTF1a, all known as markers of PPs. This module pair represents the PP signatures preferential to protocol A but also present in the GFP+ populations from the other two protocols.

RNA modules R_M2 and R_M3 were both highly correlated with ATAC module A_M2 (Figure 4GIII) and represented genes preferentially expressed in all cell populations generated with protocol B. We noticed that the corresponding A_M2 open chromatin peaks were enriched in binding sites of HNF6 and CUX2, characteristic of the previously described PP open chromatin signature. However, we also noticed that the A_M2 module was the only module with significant enrichment in CDX2 binding sites. Based on previous reports of CDX2 regulating patterning of the intestinal epithelium (Grainger et al., 2010), we hypothesized that some of the cells differentiated with protocol B might be more likely to assume an intestinal fate. In line with this hypothesis, we found that R_M2 module genes were enriched in the intestinal gene signature of duodenum, and M3 genes in genes involved in digestive tract development and hepatic cords signature.

Finally, RNA gene modules M11, M13, and M16 were preferentially expressed in cells generated with protocol C. All these gene modules were correlated with ATAC module M4, particularly enriched for the FOSL2 binding sites. FOSL2 has not previously been implicated in pancreatic development. M16 module genes were enriched in the transcriptomic signature of the *in vivo* fetal multipotent PPs; however, both modules M11 and M16 showed enrichment in ileum gene signature, and M13 additionally in hepatic cords signature.

Given the magnitude of differences between the PPs derived with the different protocols reported here, we hypothesized that among the differentially expressed genes we might also find genes with well-established roles in pancreatic and endocrine development, and therefore of particular interest to the scientific community. We therefore investigated in more detail the gene expression profiles of genes previously implicated in maturity-onset diabetes of the young and neonatal diabetes (Flanagan et al., 2014; Yang and Chan, 2016) (Figure S3). For several genes we observed significant differences in the magnitude of expression between protocols, but we also found that some genes were only highly expressed in one or two of the protocols (e.g., *NKX2.2* in protocol A, *PPARG* in protocol B, *NEUROG3* in protocols A and C). We also observed some notable differences in the sorted cell population where a gene was preferentially expressed (e.g., the *RFX6* gene was enriched in the GFP− population of protocols A and C, while for protocol B both the GFP+ and GFP− cell population expressed *RFX6*). We anticipate that findings of any hPSC disease modeling studies for the genes highlighted here would be highly dependent on the choice of differentiation protocol.

In summary, analysis of pairs of correlated modules of gene expression and open chromatin provide an objective way to characterize the cell populations common and specific to each protocol. In addition, through gene ontology and transcription motif enrichment analyses, we were able to highlight the distinct developmental programs active in different protocols, as well as the TFs likely to control them.

Variable Efficiency of Differentiation of PPs Toward the Endocrine Lineage

We next sought to evaluate the ability of the PPs derived from the three protocols to differentiate toward the pancreatic endocrine lineage. To this end, we applied stage 5 and stage 6 of the pancreas endocrine differentiation protocol developed by Rezania et al. (2014) (Figure S4A). While this protocol did not give rise to mature endocrine cells in our hands (Figure S4B), we have previously shown it to be useful for studying endocrine development *in vitro* (Petersen et al., 2017; Ramond et al., 2018). The endocrine differentiation was evaluated for expression of the endocrine markers NEUROD1 and NKX2.2 at stage 5 and for expression of C-peptide, a marker of endogenous insulin production, at stage 6. Interestingly, protocol A displayed expression of NEUROD1 and NKX2.2 in a significant percentage of cells after three days of differentiation, whereas the endocrine induction from protocol B and C was significantly lower (Figures S4C and S4D). The poor endocrine induction of protocols B and C was also evident at stage 6 of the differentiation protocol, where only very few C-peptide+ cells were observed from protocols B and C (Figures S4E–S4G). In contrast, protocol A displayed a robust

induction of C-peptide+ cells at stage 6 of the protocol, with several of C-peptide+ cells co-expressing NKX6.1, indicative of differentiation toward beta-like cells (Figures S4E–S4G).

Improved Pancreatic Endocrine Differentiation Following Reduction of the Intestinal Marker CDX2

We next examined whether the RNA-seq and ATAC-seq profiles of the PPs could inform us on how to improve the endocrine differentiation. As described previously, we identified a gene module R_M2 preferentially expressed in protocol B that showed enrichment in an intestinal gene expression signature. We found that the correlated open chromatin module A_M2 was enriched in binding sites of the canonical intestinal TF CDX2. We confirmed that CDX2 was a member of the R_M2 gene module, and its expression was enriched in the NKX6.1+ cells of protocol B compared with protocols A and C (Figures 5A and 5B). We also observed CDX2-positive cells in protocol A, but in contrast to protocol B these were primarily NKX6.1-negative cells (Figures 5A, 5B, and S5). Omission of the BMP antagonist from protocol A led to a substantial increase in the CDX2-positive cells at the expense of NKX6.1-positive cells, suggesting that BMP signaling must be actively inhibited to prevent differentiation toward intestinal lineages. In contrast, addition of EGF during stage 4 of protocol A resulted in a significant reduction in CDX2-positive cells compared with the standard protocol A (Figure S5).

We hypothesized that the intestinal gene expression signature detected in PPs of protocol B could explain the low efficiency of differentiation toward the endocrine lineage (Figures S4C and S4D). To test this hypothesis, we evaluated ten modifications of protocol B, including several conditions with BMP antagonists aiming at maintaining the expression of the PP markers PDX1 and NKX6.1, while reducing the expression of CDX2. Interestingly, several of the modified conditions of protocol B maintained a high proportion of PDX1 and NKX6.1-positive cells (Figures 5C and 5E), but only two of these conditions (condition 3 and 5) also demonstrated a substantial reduction in CDX2 expression (Figures 5D and 5E). We subsequently evaluated the expression of select markers of the pancreatic and intestinal lineage in one of the modifications of protocol B resulting in reduction of CDX2 expression (condition 3). As expected, *CDX2* mRNA expression was downregulated in the modified protocol, as were several other genes from the intestinal gene expression signature, while several genes related to pancreas development were upregulated (Figure S5D).

We then tested whether the reduced CDX2 expression in protocol B resulted in improved ability to differentiate toward the pancreatic endocrine lineage. To this end, we differentiated PPs with protocol B, as well as its two modifications resulting in reduced CDX2 expression toward endocrine progenitors and endocrine cells, as described above (Figure S4A). Interestingly, inclusion of Noggin in stage 2–4 of protocol B (condition 3) resulted in a significant increase in the number of cells expressing the endocrine progenitor markers NEUROD1 and NKX2.2 (Figure 6A), compared with protocol B without the presence of a BMP inhibitor. Despite the reduced CDX2 expression in PPs in condition 5, this did not result in an improved differentiation toward the endocrine lineage (Figure 6A). To test whether the increased endocrine differentiation observed with the inclusion of Noggin in stage 2–4 of protocol B led to an increase in generation of beta-like cells, the cells were differentiated to the end of stage 6 (Rezania et al., 2014) (Figure S4A). We observed a significant increase in cells co-expressing the beta cell markers C-peptide and NKX6.1, when Noggin was included during stage 2–4 of protocol B (condition 3) compared with the standard protocol B (Figures 6B–6D).

DISCUSSION

In the present study, we applied three differentiation protocols for deriving PPs from hPSCs (Nostro et al., 2015; Rezania et al., 2014; Russ et al., 2015). All three protocols allowed for efficient generation of hPSC-derived PPs at levels comparable with the original reports. Interestingly, we noticed that certain hPSC lines displayed varying efficiency depending on the protocol applied. Variation in the ability to differentiate toward various lineages across hPSC lines has previously been reported (Osafune et al., 2008; Rouhani et al., 2014); however, our study highlights that such differences could possibly also be specific to the protocol used for deriving a particular cell lineage. We further demonstrated that modifications can be made to protocols, allowing for increasing the efficiency of otherwise poorly differentiating hPSC lines. Exclusion of Noggin during stage 2 rescued the ability of one hPSC line to generate PPs with protocol C. Previous studies investigated the effect of modulating the BMP signaling pathway during specification to the pancreatic lineage (Nostro et al., 2011; Shahjalal et al., 2014) but our results suggest that BMP signaling may influence the cell lineage choice at even earlier stages of differentiation. The effect of Noggin appeared to be dependent on the differentiation protocols but also of the hPSC lines applied. This suggests that variation in endogenous BMP signaling during the early stages of differentiation can influence the choice of fate of hPSC lines and modulation thereof could be an avenue for further protocol optimization for hPSC lines that prove difficult to differentiate toward the pancreatic lineage.

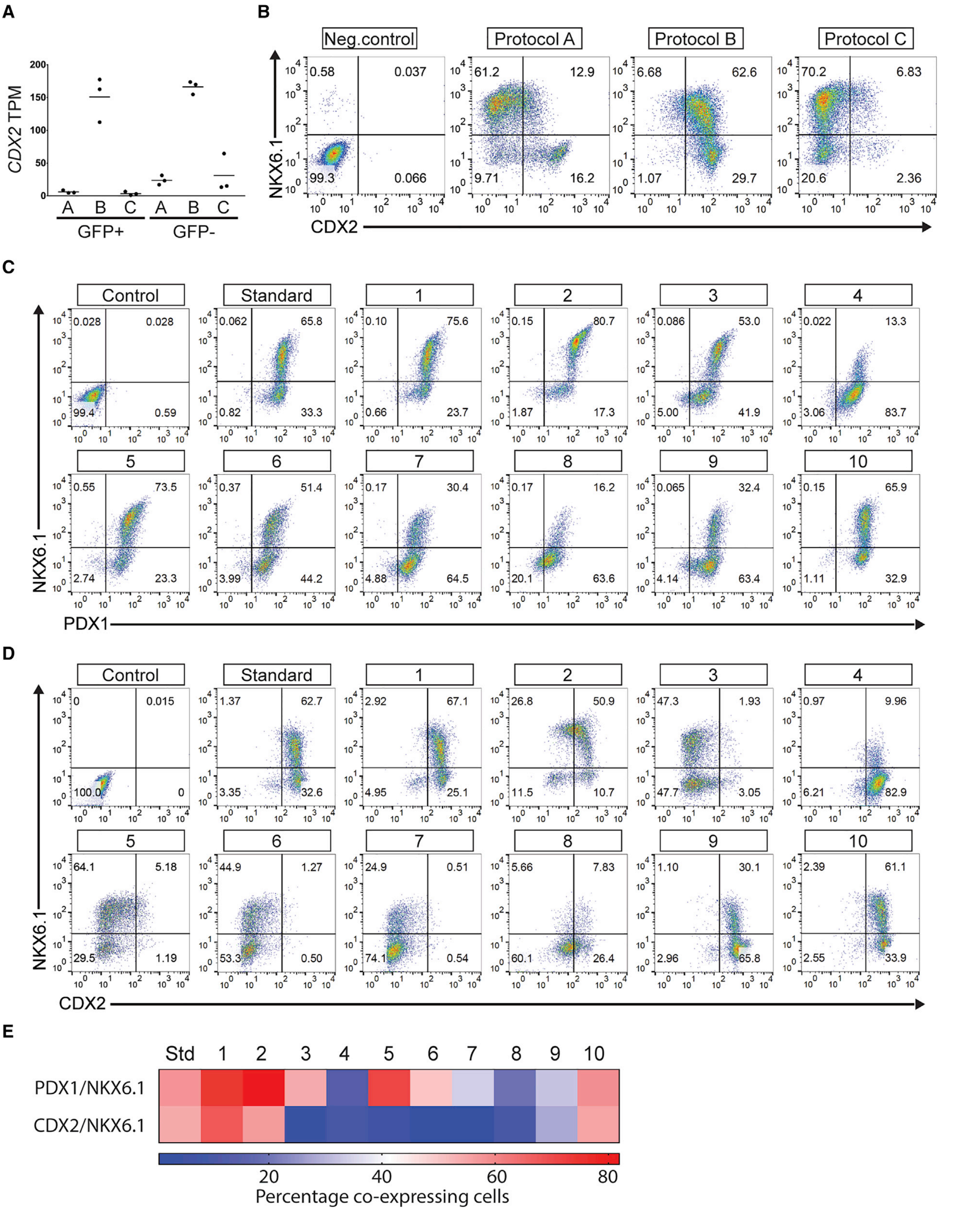

(legend on next page)

We performed RNA-seq and ATAC-seq of isolated NKX6.1-positive cells, which allowed us to define a transcriptomic and epigenomic signature for *in vitro*-generated PPs from the three differentiation protocols, through comparison with equivalent data from all the stages of hPSC differentiation toward beta-like cells. Of note, this approach excludes genes expressed at multiple stages of the hPSC differentiation toward beta-like cells and therefore not unique to the PP stage, including *PDX1* and *NKX6.1*, which are continuously expressed from the posterior foregut and pancreatic progenitor stages of the differentiation protocol, respectively. Globally, the gene expression and chromatin accessibility profiles for cells generated with all three protocols clustered together with the pancreatic endoderm stage samples of the control lines. *In vivo* differentiation of hPSC-derived PPs to the specific lineages of the pancreas is considered the most stringent method for evaluating the competence of these cells. Thus, one limitation in the current study is that we did not evaluate the competence of the PPs from the three protocols *in vivo*. Nonetheless, the PPs of protocols A and C showed significant similarities to the *in vivo* PPs derived from human fetal pancreas, suggesting that these protocols recapitulate several aspects of human pancreas development. Among the genes forming the *in vitro* PP signature we found several genes involved in pancreas development, including *ONECUT1* (Jacquemin et al., 2003) and *SOX9* (Seymour et al., 2007), as well as genes involved in Notch and Wnt signaling pathways, with previously well-established functions in pancreatic development (Pan and Wright, 2011). The open chromatin regions forming the epigenomic PP signature were significantly enriched in binding motifs of several known pancreatic TFs, including HNF6, HNF1B, FOXA2, PDX1, and SOX9. We observed that CUX2 motifs were the most significantly enriched in these common open chromatin regions. In line with this, we found that the *CUX2* gene's expression during differentiation peaks at the pancreatic endoderm stage, and we observed evidence for CUX2 being bound within the open chromatin of the samples generated in this study through ATAC-seq footprinting analysis. Interestingly, a previous study demonstrated the expression of *CUX2* in early fetal human dorsal pancreas (Jennings et al., 2017), and more recently the presence of CUX2 motifs in accessible chromatin regions were shown to be differentially enriched in mouse endocrine progenitors across different stages of development (Scavuzzo et al., 2018). Based on this evidence, we suggest CUX2 as a potential novel regulator of pancreatic development.

We then sought to highlight the protocol-specific differences in the omics profiles of the PPs. We focused on modules of co-expressed genes and correlated open chromatin grouping genes preferentially expressed in each of the protocols. We observed a significantly higher endocrine commitment in the NKX6.1-negative compartment of protocol A compared with protocols B and C. This is in agreement with the original reports, where Nostro et al. (2015) and Russ et al. (2015) developed protocols with the specific purpose of limiting the endocrine differentiation in place of PPs. Thus, we were able to recapitulate important features of the three differentiation protocols. Interestingly, the NKX6.1-negative population of protocol A clustered closely together with isolated human fetal endocrine progenitors (Ramond et al., 2018), suggesting that the endocrine differentiation observed *in vitro* shares many similarities with fetal endocrine development. On the other hand, genes with the highest expression in the GFP+ population from protocol A were enriched in gene signatures of the *in vivo* fetal PPs from the same study, as well as in the signatures of *in vitro* PPs from this and a previous study (Cebola et al., 2015).

Further, we noted that some of the genes preferentially expressed in cells generated with protocol B were suggestive of these cells assuming a non-endocrine fate. We noted enrichments in genes forming previously reported intestinal signatures, and we observed that open chromatin regions correlated with expression of these genes were enriched in binding motifs of CDX2, a marker of intestinal development, and *CDX2* gene expression was also most highly expressed in protocol B cells. A recent study

Figure 5. Reduction of Expression of the Intestinal Marker CDX2 in PPs

(A) TPM for *CDX2* in GFP− and GFP+ sorted cell populations from differentiation protocols A, B, and C. Graph shows scatterplot of mean with each dot representing individual differentiations.

(B) Flow cytometry analysis of NKX6.1 and CDX2 of PPs from the three differentiation protocols. DE was used as negative control. Representative pseudo color dot plots of five individual differentiations.

(C and D) Representative pseudo color dot plots of two individual differentiations. Ten modifications of protocol B were assessed for the ability to maintain PDX1 and NKX6.1 expression (C) while simultaneously reducing expression of CDX2 (D). Representative pseudo color dot plots of cells stained for NKX6.1 and PDX1 (C) or NKX6.1 and CDX2 (D). DE cells were used as negative controls.

(E) Heatmap summarizing the percentage of PDX1/NKX6.1 and CDX2/NKX6.1 co-expressing cells. Average percentage of one differentiation each of SB AD3.1 and SB AD3.4 hiPSC lines (n = 2 independent differentiations). Conditions tested were (1) 50 ng/mL Noggin, ST2; (2) 50 ng/mL Noggin, ST2-3; (3) 50 ng/mL Noggin, ST2-4; (4) ST4 only 2 days; (5) 100 nM LDN, ST2; (6) 100 nM LDN, ST2-3; (7) 100 nM LDN, ST2-4; (8) 50 ng/mL Activin A, ST4.2; (9) without KGF, ST4.2; (10) reduced retinoic concentration (0.2 μM) second day of ST3.

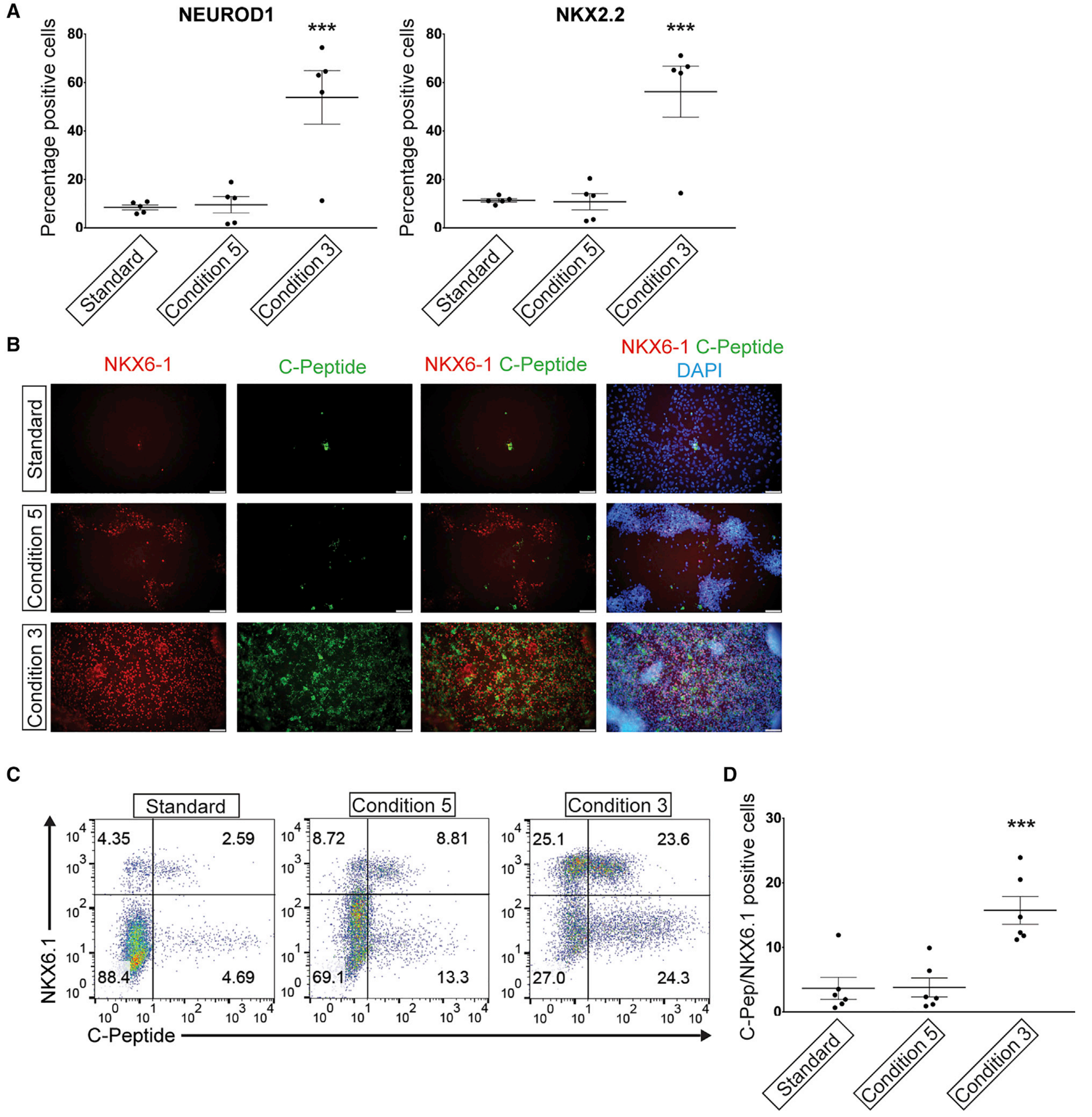

Figure 6. Improved Endocrine Differentiation Following Reduction of CDX2 Expression in PPs

(A) Flow cytometry-based quantifications of stage 5 endocrine progenitor differentiation of protocol B and the two modified conditions (condition 5 and 3). Scatterplots show percentage of NEUROD1 and NKX2.2-positive cells as means ± SEM, n = 5 independent experiments (three with SB AD3.1 hiPSC, one with SB AD3.4 hiPSC, and one with SA121 hESC).

(B) Immunofluorescence microscopy images of stage 6 cells stained with NKX6.1 and C-peptide antibodies. DAPI is used to visualize the nuclei of all cells. Scale bar, 100 μm.

(C) Representative pseudo color dot plots of stage 6 beta-like cells stained for C-peptide and NKX6.1. Numbers mark the percentage of cells in each quadrant.

(legend continued on next page)

reported the expression of *CDX2* in the dorsal pancreas of human fetal tissue (Jennings et al., 2017). However, we have previously performed single-cell gene expression analysis of human fetal pancreata and found no evidence of *CDX2* expression in human PPs (Ramond et al., 2018). This discrepancy could perhaps be explained by the different stages of human pancreas development interrogated in the two studies. Thus, it remains unclear whether PDX1, NKX6.1, and CDX2 co-expressing progenitor cells occur at some point during human pancreas development. CDX2-positive cells were also observed in protocol A but the majority of these were NKX6.1 negative. Excluding the BMP antagonists from protocol A resulted in a significant increase of CDX2-positive cells at the expense of NKX6.1-positive PPs, illustrating that inhibiting BMP signaling is necessary to prevent differentiation toward an intestinal fate. This observation is in agreement with a previous study demonstrating that BMP signaling inhibition during differentiation of hPSCs to PPs resulted in reduced *CDX2* expression (Shahjalal et al., 2014). Interestingly, addition of EGF to the final stage of protocol A resulted in a reduction of the percentage of CDX2-positive cells. These results illustrate how evaluating individual components of a differentiation protocol can elucidate their specific roles in the differentiation and guide improving the protocol efficiency.

We hypothesized that *CDX2* expression and the intestinal gene signature enriched in the PPs from protocol B could suggest a more posterior patterning during the differentiation, which might explain the inability of the PPs from protocol B to differentiate further toward the pancreatic endocrine lineage. In the present study, we found that inclusion of the BMP antagonist Noggin resulted in reduced expression of *CDX2* and other genes specific to the intestinal gene expression module, and subsequently in successful differentiation down the endocrine lineage. Interestingly, another BMP antagonist, LDN193189, also reduced CDX2 expression, but this was not accompanied by an improved differentiation toward the endocrine lineage. The reason for this difference is unclear but may relate to the different mechanisms of the two BMP antagonists. Together, these results illustrate the importance of monitoring CDX2 expression during differentiation toward PPs. Interestingly, the original report describing protocol B argued for the omission of BMP antagonist during pancreatic specification in order for efficient derivation of PDX1/NKX6.1 co-expressing PPs (Russ et al., 2015); however, CDX2 expression was not assessed in this study. It thus remains unclear whether CDX2 expression in the PPs is inherent to this protocol or a consequence of differences in experimental conditions and cell lines applied in this study compared with the original study (Russ et al., 2015). It is also plausible that, since the endocrine differentiation protocol applied in this study was originally developed in combination with protocol A (Rezania et al., 2014), it may not be directly applicable to other protocols. Whether there are differences in the signaling pathways that promote endocrine differentiation from the PPs derived from protocols A, B, and C remains to be shown.

There are many challenges associated with faithfully reproducing differentiation protocols, most recently demonstrated in a study comparing iPSC-derived neurons using a well-defined protocol across five different laboratories (Volpato et al., 2018). In addition, several modifications were made to the protocols applied in the current study compared with the original publications. Thus, it should be emphasized that our study should not be seen as a direct head-to-head comparison of the three protocols. Nonetheless, our work demonstrates the importance of assessing apparently similar cell populations derived using distinct differentiation protocols. This approach allowed us to define a comprehensive transcriptional and epigenomic signature for PPs that will serve as a valuable resource for studying pancreas development. However, our findings also highlighted significant variation in the PPs derived from the three protocols, which should warrant caution for using hPSCs to interrogate roles of specific genes in disease and development. Finally, we demonstrate the utility of benchmarking *in vitro*-derived cell populations to their *in vivo* counterparts, which allows for the identification of markers useful for improving the differentiation of hPSCs toward beta-like cells.

EXPERIMENTAL PROCEDURES

Please refer to Supplemental Experimental Procedures for detailed description of experimental procedures.

Maintenance and Differentiation of hPSC Lines

All hPSCs were maintained on human embryonic stem cell-qualified Matrigel in mTeSR1 medium, except for the NKX6.1-GFP hiPSC line, which was cultured in TeSR1-E8 medium, at 37°C, 5% CO_2, and passaged at 90%–95% confluence using TrypLE select, as previously described (Perez-Alcantara et al., 2018).

(D) Quantification of C-peptide/NKX6.1 double positive by flow cytometry as shown in (C) (conditions 5 and 3). Scatterplot shows percentage of C-peptide/NKX6.1 double-positive cells as means ± SEM, n = 6 independent experiments (four with SB AD3.1 hiPSC, two with SA121 hESC).
(A and D) One-way ANOVA with Tukey test for multiple comparisons, ***p < 0.001, different from the two other groups.

FACS

Briefly, differentiated cells were harvested to a single-cell suspension using TrypLE select, pelleted and resuspended in the stage 4 medium of the respective protocols (without factors) containing 1 μg/mL DAPI solution, and directly proceeded for sorting. Cells were sorted using a BD FACSAria Fusion (BD Biosciences).

RNA-seq

For RNA-seq, cells were pelleted immediately following sorting and medium was removed. Cells were harvested and RNA extracted using TRIzol Reagent (ThermoFisher Scientific, Paisley, UK) as per the manufacturer's guidelines. Smart-Seq2 paired-end RNA-seq libraries were sequenced on Illumina HiSeq4000 to a mean depth of 37.7 (± 1.8) million 75 bp reads pairs per sample.

Transposition Reaction and Purification for ATAC-Seq

Following sorting, cells were pelleted and washed in cold PBS. Cell pellets were gently resuspended in cold lysis buffer (10 mM Tris-HCL, pH 7.4, 10 mM NaCl, 3 mM $MgCl_2$, 0.1% IGEPAL CA-630) and immediately pelleted by centrifugation. The supernatant was discarded and the cell pellets were gently resuspended in 50 μL of transposition reaction mix (25 μL 2 of TD buffer, 2.5 μL of Tn5 Transposase [Illumina], 22.5 μL of nuclease-free H_2O) and incubated at 37°C for 30 min. Following the transposition reaction, the transposed DNA was purified using QIAGEN's MinElute Kit according to manufacturer's instructions and eluted in 10 μL of elution buffer (10 mM Tris buffer, pH 8).

Statistics

Statistical analyses were performed using GraphPad Prism (V8.0.2). Means were compared with unpaired Student's t test and one-way ANOVA with Tukey test for multiple comparisons.

ACCESSION NUMBERS

RNA-seq and ATAC-seq raw data have been deposited in the European Genome-phenome Archive, accession number EGAS00001003513. Dataset (RNA-seq): EGAS00001004823. Dataset (ATAC-seq): EGAS00001004824.

SUPPLEMENTAL INFORMATION

Supplemental Information can be found online at https://doi.org/10.1016/j.stemcr.2019.11.010.

AUTHOR CONTRIBUTIONS

A.W.A. performed all bioinformatics analysis, interpreted the data, and wrote the manuscript. R.R.J. performed *in vitro* differentiation experiments, data analysis, and interpreted data. M.P.A. provided data for ATAC-seq analysis of control iPSC lines and performed bioinformatics analysis of these. N.L.B., C.D., V.N., M.G., L.W., and R.B. provided experimental and scientific input. M.I.M., M.H. and A.L.G. provided scientific input and edited the manuscript. C.H. performed *in vitro* differentiation experiments, data analysis, interpreted data, and wrote the manuscript. All authors approved the manuscript.

ACKNOWLEDGMENTS

A.L.G. is a Wellcome Trust Senior Fellow in Basic Biomedical Science. M.I.M. is a Wellcome Senior Investigator. This work was funded in Oxford by the Wellcome Trust (095101 [A.L.G.], 200837 [A.L.G.], 098381 [M.I.M.], 212259 [M.I.M.] 106130 [A.L.G., M.I.M.], 203141 (A.L.G., M.I.M.], 203141 [M.I.M.], Medical Research Council (MR/L020149/1) [M.I.M., A.L.G.], European Union Horizon 2020 Programme (T2D Systems) [A.L.G.], and NIH (U01-DK105535; U01-DK085545) [M.I.M., A.L.G.]. The research was funded by the National Institute for Health Research (NIHR) Oxford Biomedical Research Centre (BRC) [A.L.G., M.I.M.]. The views expressed are those of the author(s) and not necessarily those of the NHS, the NIHR, or the Department of Health. Furthermore, the research leading to these results has received support from the Innovative Medicines Initiative (IMI) Joint Undertaking (JU) under grant agreement n° 115439, resources of which are composed of financial contribution from the European Union's Seventh Framework Programme (FP7/2007-2013) and European Federation of Pharmaceutical Industries and Associations (EFPIA) companies' in kind contribution with additional specific support from Eli Lilly. This publication reflects only the authors' views and neither the IMI JU nor EFPIA nor the European Commission are liable for any use that may be made of the information contained therein. In addition, this work was in part supported by the Wellcome Trust via core funding to the Wellcome Centre for Human Genetics (awards 090532/Z/09/Z and 203141/Z/16/Z). M.H., R.R.J., and C.H. are employees of Novo Nordisk A/S and hold shares in Novo Nordisk A/S. N.L.B. is an employee of Novo Nordisk Ltd, but all experimental work was carried out while under the employment of University of Oxford. M.I.M. is a Wellcome Senior Investigator and an NIHR Senior Investigator. He serves on advisory panels for Pfizer, NovoNordisk, Zoe Global; has received honoraria from Merck, Pfizer, NovoNordisk, and Eli Lilly; has stock options in Zoe Global; and has received research funding from Abbvie, Astra Zeneca, Boehringer Ingelheim, Eli Lilly, Janssen, Merck, NovoNordisk, Pfizer, Roche, Sanofi Aventis, Servier, and Takeda. The views expressed in this article are those of the authors and not necessarily those of the NHS, the NIHR, or the Department of Health.

Received: April 23, 2019
Revised: November 29, 2019
Accepted: November 29, 2019
Published: December 26, 2019

REFERENCES

Bhushan, A., Itoh, N., Kato, S., Thiery, J.P., Czernichow, P., Bellusci, S., and Scharfmann, R. (2001). Fgf10 is essential for maintaining the proliferative capacity of epithelial progenitor cells during early pancreatic organogenesis. Development *128*, 5109–5117.

Cebola, I., Rodriguez-Segui, S.A., Cho, C.H., Bessa, J., Rovira, M., Luengo, M., Chhatriwala, M., Berry, A., Ponsa-Cobas, J., Maestro, M.A., et al. (2015). TEAD and YAP regulate the enhancer network of human embryonic pancreatic progenitors. Nat. Cell Biol. *17*, 615–626.

Flanagan, S.E., De Franco, E., Lango Allen, H., Zerah, M., Abdul-Rasoul, M.M., Edge, J.A., Stewart, H., Alamiri, E., Hussain, K., Wallis, S., et al. (2014). Analysis of transcription factors key for mouse pancreatic development establishes NKX2-2 and MNX1 mutations as causes of neonatal diabetes in man. Cell Metab. *19*, 146–154.

Grainger, S., Savory, J.G., and Lohnes, D. (2010). Cdx2 regulates patterning of the intestinal epithelium. Dev. Biol. *339*, 155–165.

Gupta, S.K., Wesolowska-Andersen, A., Ringgaard, A.K., Jaiswal, H., Song, L., Hastoy, B., Ingvorsen, C., Taheri-Ghahfarokhi, A., Magnusson, B., Maresca, M., et al. (2018). NKX6.1 induced pluripotent stem cell reporter lines for isolation and analysis of functionally relevant neuronal and pancreas populations. Stem Cell Res. *29*, 220–231.

Jacquemin, P., Lemaigre, F.P., and Rousseau, G.G. (2003). The Onecut transcription factor HNF-6 (OC-1) is required for timely specification of the pancreas and acts upstream of Pdx-1 in the specification cascade. Dev. Biol. *258*, 105–116.

Jennings, R.E., Berry, A.A., Strutt, J.P., Gerrard, D.T., and Hanley, N.A. (2015). Human pancreas development. Development *142*, 3126–3137.

Jennings, R.E., Berry, A.A., Gerrard, D.T., Wearne, S.J., Strutt, J., Withey, S., Chhatriwala, M., Piper Hanley, K., Vallier, L., Bobola, N., et al. (2017). Laser capture and deep sequencing reveals the transcriptomic programmes regulating the onset of pancreas and liver differentiation in human embryos. Stem Cell Reports *9*, 1387–1394.

Kelly, O.G., Chan, M.Y., Martinson, L.A., Kadoya, K., Ostertag, T.M., Ross, K.G., Richardson, M., Carpenter, M.K., D'Amour, K.A., Kroon, E., et al. (2011). Cell-surface markers for the isolation of pancreatic cell types derived from human embryonic stem cells. Nat. Biotechnol. *29*, 750–756.

Kroon, E., Martinson, L.A., Kadoya, K., Bang, A.G., Kelly, O.G., Eliazer, S., Young, H., Richardson, M., Smart, N.G., Cunningham, J., et al. (2008). Pancreatic endoderm derived from human embryonic stem cells generates glucose-responsive insulin-secreting cells in vivo. Nat. Biotechnol. *26*, 443–452.

Langfelder, P., and Horvath, S. (2008). WGCNA: an R package for weighted correlation network analysis. BMC Bioinformatics *9*, 559.

Larsen, H.L., and Grapin-Botton, A. (2017). The molecular and morphogenetic basis of pancreas organogenesis. Semin. Cell Dev. Biol. *66*, 51–68.

Molotkov, A., Molotkova, N., and Duester, G. (2005). Retinoic acid generated by Raldh2 in mesoderm is required for mouse dorsal endodermal pancreas development. Dev. Dyn. *232*, 950–957.

Nair, G., and Hebrok, M. (2015). Islet formation in mice and men: lessons for the generation of functional insulin-producing beta-cells from human pluripotent stem cells. Curr. Opin. Genet. Dev. *32*, 171–180.

Nostro, M.C., Sarangi, F., Ogawa, S., Holtzinger, A., Corneo, B., Li, X., Micallef, S.J., Park, I.H., Basford, C., Wheeler, M.B., et al. (2011). Stage-specific signaling through TGFbeta family members and WNT regulates patterning and pancreatic specification of human pluripotent stem cells. Development *138*, 861–871.

Nostro, M.C., Sarangi, F., Yang, C., Holland, A., Elefanty, A.G., Stanley, E.G., Greiner, D.L., and Keller, G. (2015). Efficient generation of NKX6-1+ pancreatic progenitors from multiple human pluripotent stem cell lines. Stem Cell Reports *4*, 591–604.

Osafune, K., Caron, L., Borowiak, M., Martinez, R.J., Fitz-Gerald, C.S., Sato, Y., Cowan, C.A., Chien, K.R., and Melton, D.A. (2008). Marked differences in differentiation propensity among human embryonic stem cell lines. Nat. Biotechnol. *26*, 313–315.

Pan, F.C., and Wright, C. (2011). Pancreas organogenesis: from bud to plexus to gland. Dev. Dyn. *240*, 530–565.

Perez-Alcantara, M., Honore, C., Wesolowska-Andersen, A., Gloyn, A.L., McCarthy, M.I., Hansson, M., Beer, N.L., and van de Bunt, M. (2018). Patterns of differential gene expression in a cellular model of human islet development, and relationship to type 2 diabetes predisposition. Diabetologia *61*, 1614–1622.

Petersen, M.B.K., Azad, A., Ingvorsen, C., Hess, K., Hansson, M., Grapin-Botton, A., and Honore, C. (2017). Single-cell gene expression analysis of a human ESC model of pancreatic endocrine development reveals different paths to beta-cell differentiation. Stem Cell Reports *9*, 1246–1261.

Petersen, M.B.K., Goncalves, C.A.C., Kim, Y.H., and Grapin-Botton, A. (2018). Recapitulating and deciphering human pancreas development from human pluripotent stem cells in a dish. Curr. Top. Dev. Biol. *129*, 143–190.

Ramond, C., Beydag-Tasoz, B.S., Azad, A., van de Bunt, M., Petersen, M.B.K., Beer, N.L., Glaser, N., Berthault, C., Gloyn, A.L., Hansson, M., et al. (2018). Understanding human fetal pancreas development using subpopulation sorting, RNA sequencing and single-cell profiling. Development *145*. https://doi.org/10.1242/dev.165480.

Rezania, A., Bruin, J.E., Riedel, M.J., Mojibian, M., Asadi, A., Xu, J., Gauvin, R., Narayan, K., Karanu, F., O'Neil, J.J., et al. (2012). Maturation of human embryonic stem cell-derived pancreatic progenitors into functional islets capable of treating pre-existing diabetes in mice. Diabetes *61*, 2016–2029.

Rezania, A., Bruin, J.E., Xu, J., Narayan, K., Fox, J.K., O'Neil, J.J., and Kieffer, T.J. (2013). Enrichment of human embryonic stem cell-derived NKX6.1-expressing pancreatic progenitor cells accelerates the maturation of insulin-secreting cells in vivo. Stem Cells *31*, 2432–2442.

Rezania, A., Bruin, J.E., Arora, P., Rubin, A., Batushansky, I., Asadi, A., O'Dwyer, S., Quiskamp, N., Mojibian, M., Albrecht, T., et al. (2014). Reversal of diabetes with insulin-producing cells derived in vitro from human pluripotent stem cells. Nat. Biotechnol. *32*, 1121–1133.

Rouhani, F., Kumasaka, N., de Brito, M.C., Bradley, A., Vallier, L., and Gaffney, D. (2014). Genetic background drives transcriptional variation in human induced pluripotent stem cells. PLoS. Genet. *10*, e1004432.

Russ, H.A., Parent, A.V., Ringler, J.J., Hennings, T.G., Nair, G.G., Shveygert, M., Guo, T., Puri, S., Haataja, L., Cirulli, V., et al. (2015). Controlled induction of human pancreatic progenitors produces functional beta-like cells in vitro. EMBO J. *34*, 1759–1772.

Scavuzzo, M.A., Hill, M.C., Chmielowiec, J., Yang, D., Teaw, J., Sheng, K., Kong, Y., Bettini, M., Zong, C., Martin, J.F., et al. (2018). Endocrine lineage biases arise in temporally distinct endocrine progenitors during pancreatic morphogenesis. Nat. Commun. *9*, 3356.

Seymour, P.A., Freude, K.K., Tran, M.N., Mayes, E.E., Jensen, J., Kist, R., Scherer, G., and Sander, M. (2007). SOX9 is required for maintenance of the pancreatic progenitor cell pool. Proc. Natl. Acad. Sci. U S A *104*, 1865–1870.

Shahjalal, H.M., Shiraki, N., Sakano, D., Kikawa, K., Ogaki, S., Baba, H., Kume, K., and Kume, S. (2014). Generation of insulin-producing beta-like cells from human iPS cells in a defined and completely xeno-free culture system. J. Mol. Cell Biol. *6*, 394–408.

Volpato, V., Smith, J., Sandor, C., Ried, J.S., Baud, A., Handel, A., Newey, S.E., Wessely, F., Attar, M., Whiteley, E., et al. (2018). Reproducibility of molecular phenotypes after long-term differentiation to human iPSC-derived neurons: a multi-site omics study. Stem Cell Reports *11*, 897–911.

Wandzioch, E., and Zaret, K.S. (2009). Dynamic signaling network for the specification of embryonic pancreas and liver progenitors. Science *324*, 1707–1710.

Yang, Y., and Chan, L. (2016). Monogenic diabetes: what it teaches us on the common forms of type 1 and type 2 diabetes. Endocr. Rev. *37*, 190–222.

Stem Cell Reports, Volume *14*

Supplemental Information

Analysis of Differentiation Protocols Defines a Common Pancreatic Progenitor Molecular Signature and Guides Refinement of Endocrine Differentiation

Agata Wesolowska-Andersen, Rikke Rejnholdt Jensen, Marta Pérez Alcántara, Nicola L. Beer, Claire Duff, Vibe Nylander, Matthew Gosden, Lorna Witty, Rory Bowden, Mark I. McCarthy, Mattias Hansson, Anna L. Gloyn, and Christian Honore

SUPPLEMENTAL FIGURES

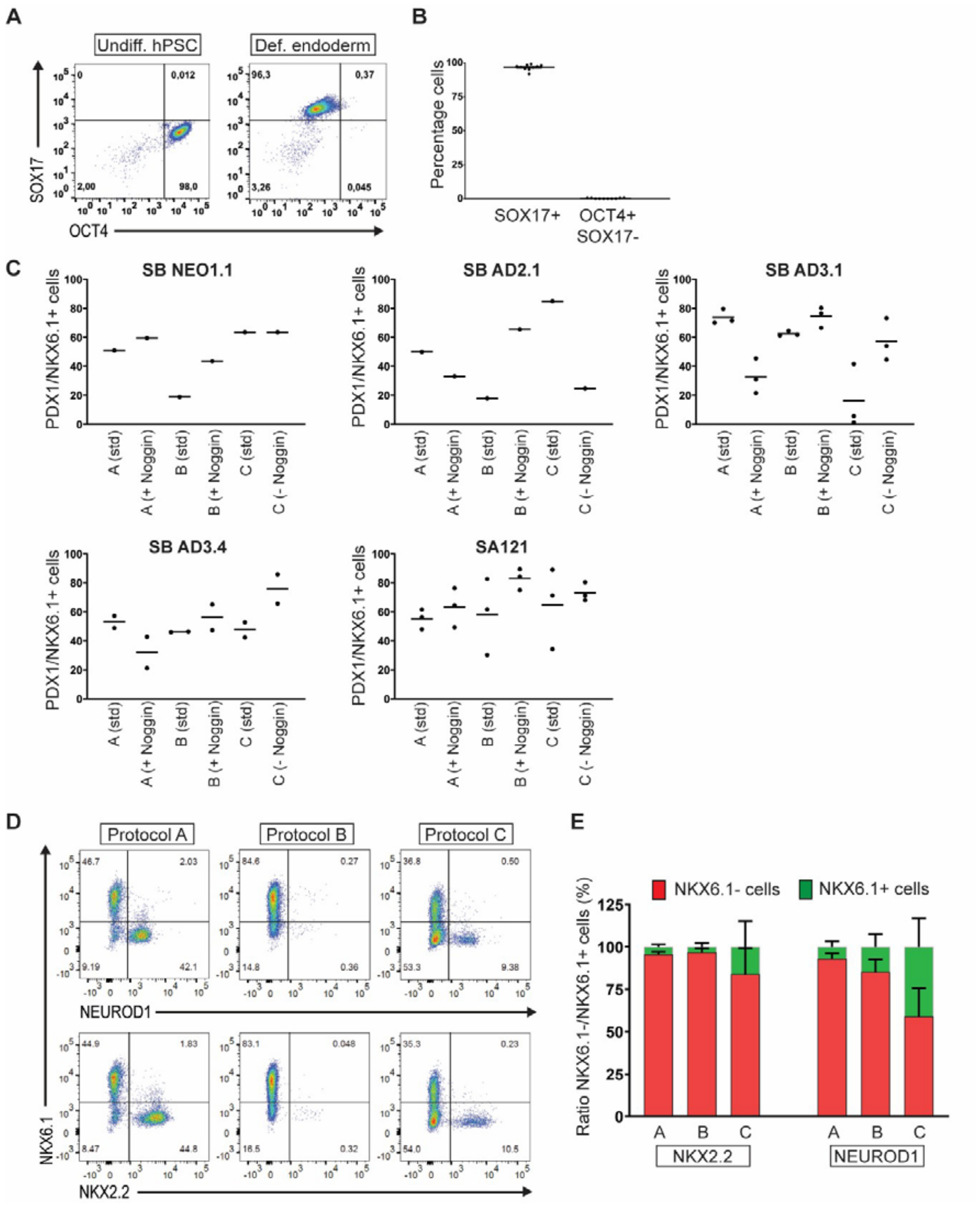

Supplementary figure 1. Definitive endoderm and pancreatic progenitor differentiation efficiency. Related to figure 1.

(A) Representative example of flow cytometry pseudo color dot plots of undifferentiated hPSC and definitive endoderm cells stained for OCT4 and SOX17. (B) Percentage of SOX17+ cells and OCT4+, SOX17- cells at the definitive endoderm stage of the protocol. Data presented as mean ± SEM, n = 10 independent experiments (SA121 hESC n = 3, SB NEO1.1 hiPSC n = 1, SB AD2.1 hiPSC n = 1, SB AD3.1 hiPSC n = 3, SB AD3.4 hiPSC n = 2). (C) Quantification of PDX1 and NKX6.1 co-expressing cells across five individual hPSC lines differentiated with protocol A, B and C. The effect of inclusion/omission of Noggin during stage 2 of each protocol was evaluated side-by-side compared to the standard protocols (outlined in figure 1A). Graphs show scatter plot of mean, n = 1-3 independent experiments, depending on cell line. (D) representative example of flow cytometry pseudo color dot plots of hPSC-derived pancreatic progenitors from protocol A, B and C. Cells are analysed for NKX6.1/NEUROD1 (top) and NKX6,1/NKX2.2 expression (bottom). (E) Ratio of NKX2.2 and NEUROD1 expressing cells negative (red) or positive (green) for NKX6.1 expression. Data presented as mean ± SEM, n = 5 independent experiments.

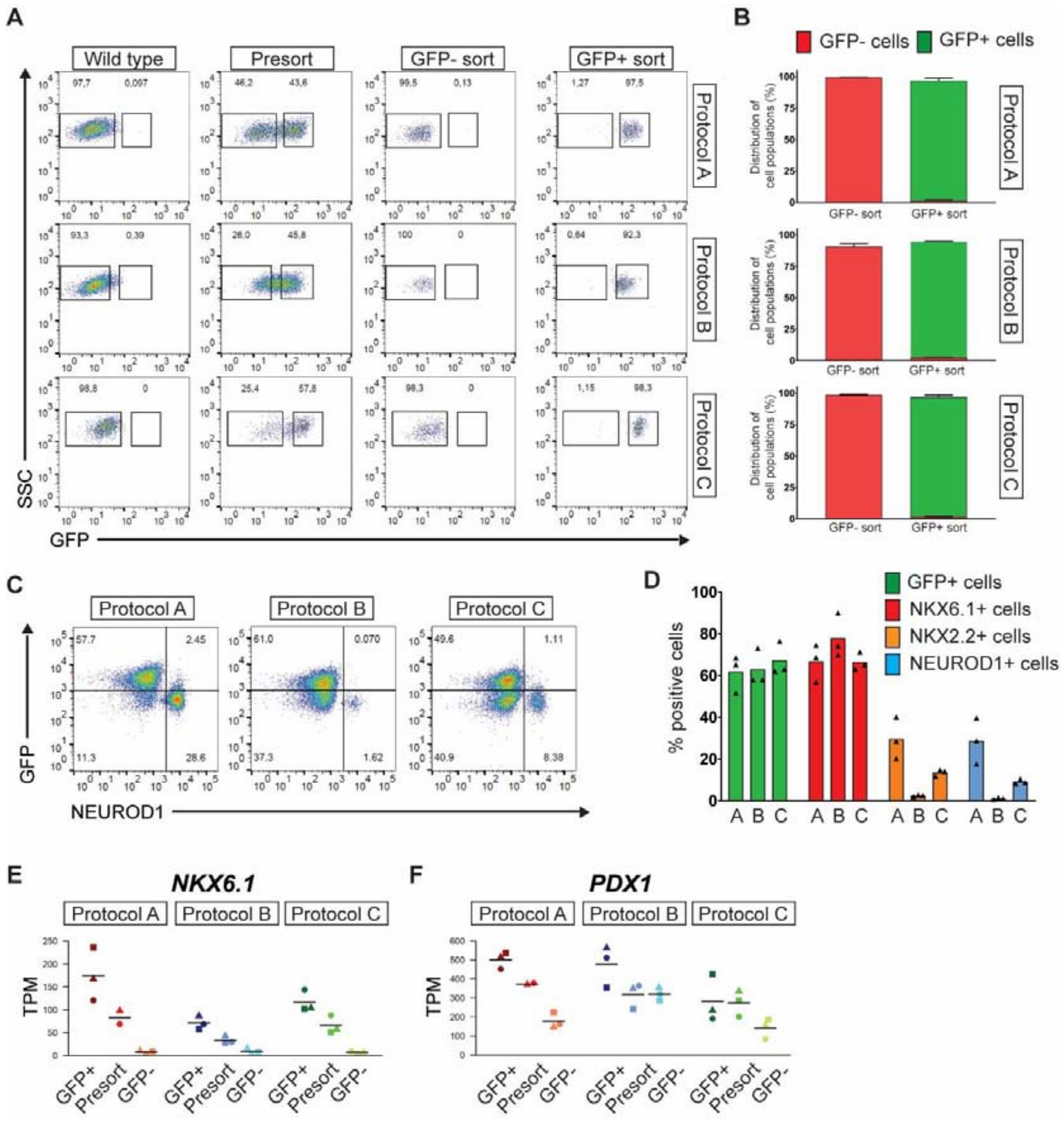

Supplementary figure 2. Confirmation of isolation of NKX6.1+ and NKX6.1- cell populations by FACS. Related to figure 2.

(A) Representative example of flow cytometry pseudo color dot plots of wild type hiPSC line (wild type), presorted NKX6.1-GFP iPSC (presort) and the GFP- and GFP+ sorted fractions from all three protocols analyzed for purity post sorting. Boxes indicates gates labelling GFP+ and GFP- cell fractions (B) Graph showing the percentage of GFP- and GFP+ cells in each of the two sorted fractions across all three differentiation protocols. Graphs shows mean ± SEM of three independent differentiations/sorting experiments (n = 3). (C) Representative examples of pseudo color dot plots of NKX6.1-GFP hiPSC line differentiated to pancreatic progenitors using the three protocols. Cells are analysed for GFP and NEUROD1 expression and illustrates that the endocrine cells (NEUROD1+ cells) are mainly found in the GFP- (NKX6.1-) compartment. (D) Flow cytometry quantification of GFP+, NKX6.1+, NKX2.2+ and NEUROD1+ cells from the three experiments used for the cell sorting experiments. Bars show mean and dots represent individual differentiations (n = 3). GFP+ expression was assessed on live cells, whereas NKX6.1, NKX2.2 and NEUROD1 expression analysis was performed on fixed and permeabilized cells. (E-F) Transcript per kilobase million (TPM) for *NKX6.1* and *PDX1* in presorted, GFP- and GFP+ sorted cell populations across all three differentiation protocols. Graphs shows scatter plot and mean, with each symbol representing an independent differentiation. *NKX6.1*: p_{adjA}=4.05e-101, p_{adjB}=1.60e-27, p_{adjC}=5.97e-64; *PDX1*: p_{adjA} =2.70e-19, p_{adjB} =8.42e-03, p_{adjC} =1.20e-07.

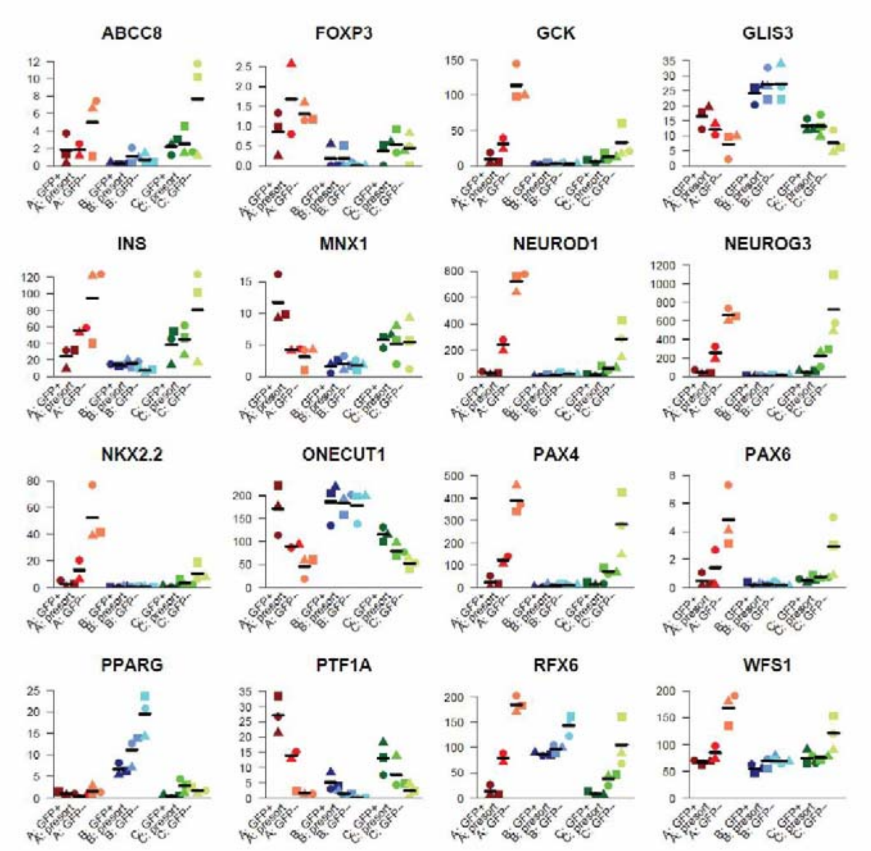

Supplementary figure 3. Expression of selected genes implicated in MODY and neonatal diabetes. Related to figure 4. Expression is shown as transcript per kilobase million (TPM) in pre-sorted, GFP- and GFP+ sorted cell populations across all three differentiation protocols. Graphs shows scatter plot and mean, with each symbol representing an independent differentiation.

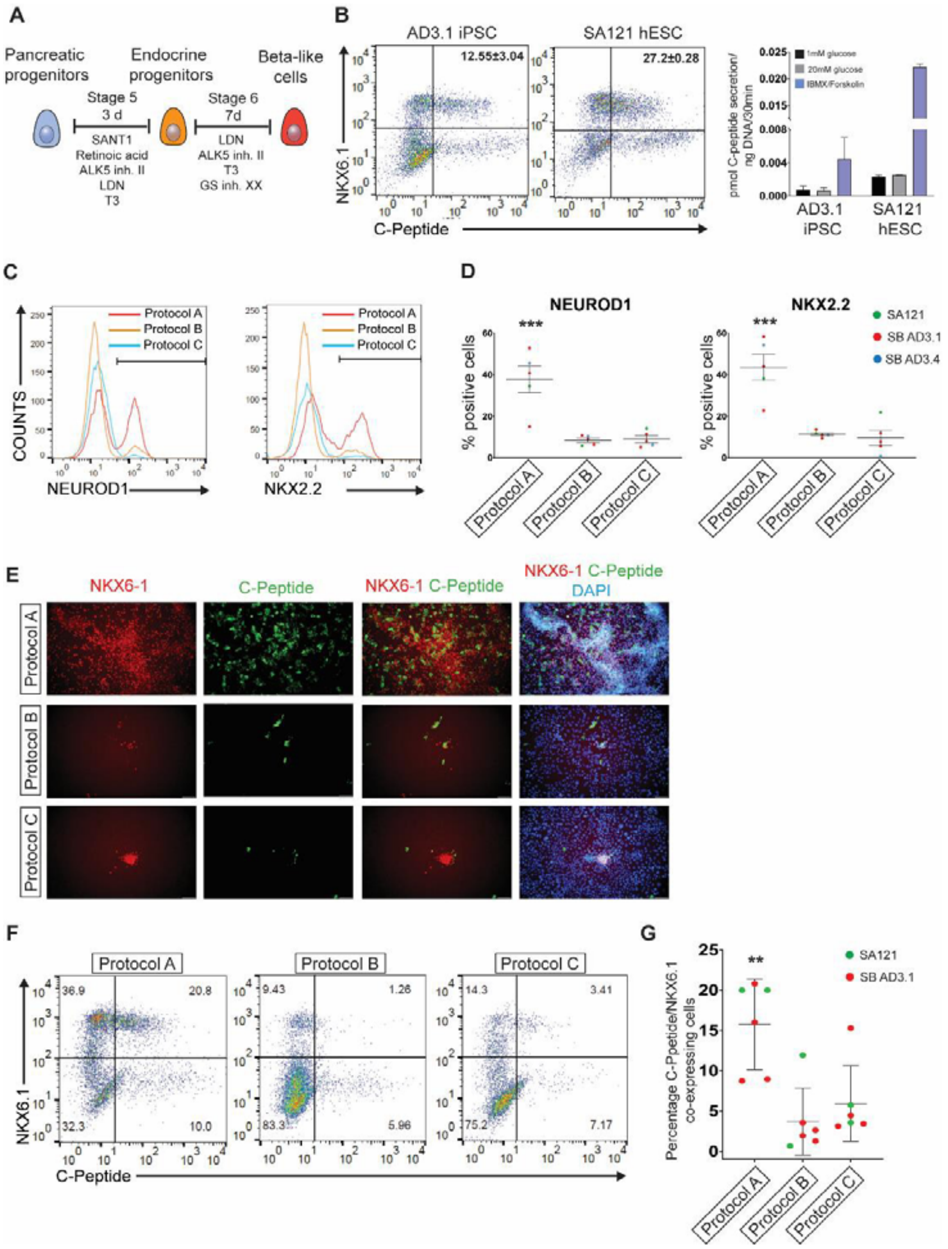
A
Pancreatic progenitors
Endocrine progenitors
Beta-like cells
Stage 5
3 d
SANT1
Retinoic acid
ALK5 inh. II
LDN
T3
Stage 6
7d
LDN
ALK5 inh. II
T3
GS inh. XX
B
AD3.1 iPSC
SA121 hESC
12.55±3.04
27.2±0.28
NKX6.1
C-Peptide
1mM glucose
20mM glucose
IBMX/Forskolin
pmol C-peptide secretion/ ng DNA/30min
AD3.1 iPSC
SA121 hESC
C
Protocol A
Protocol B
Protocol C
COUNTS
NEUROD1
NKX2.2
D
NEUROD1
NKX2.2
% positive cells
***
SA121
SB AD3.1
SB AD3.4
E
NKX6-1
C-Peptide
NKX6-1 C-Peptide
NKX6-1 C-Peptide DAPI
F
36.9
20.8
32.3
10.0
9.43
1.26
83.3
5.96
14.3
3.41
75.2
7.17
G
**
Percentage C-Ppetide/NKX6.1 co-expressing cells

Supplementary figure 4. Pancreatic endocrine differentiation of pancreatic progenitors derived from multiple protocols. **Related to figure 5**.
(A) Schematic outline of the protocol for differentiating the pancreatic progenitors from the three protocols towards first endocrine progenitors (stage 5) and subsequently towards beta-like cells (stage 6). (B) NKX6.1/C-Peptide flow cytometry pseudo color dot plots of AD3.1 iPSC and SA121 hESC lines differentiated to stage 6 for subsequent glucose stimulated insulin secretion (GSIS). Numbers in upper right quadrant shows percentage of NKX6.1+, C-Peptide+ cells (mean ± SD, n =2 for each line). Graph shows C-Peptide secretion normalized to DNA content of cells (mean ± SD, n =2 for each line). (C) Representative flow cytometry histograms demonstrating the quantification of NEUROD1 (left) and NKX2.2 (right) positive cells. Gate set based on a negative cell population and unstained cells. Color of the lines mark cells from each of the three protocols. Horizontal bar in both plots shows the gate for positive cells (D) Quantifications of NEUROD1 and NKX2.2 positive cells as shown in (C). Graphs shows percentage of NEUROD1 and NKX2.2 positive cells as mean ± SEM, n = 5 independent experiments (E) Immunofluorescence microscopy images of stage 6 cells derived from pancreatic progenitors from protocol A, B and C. Cells were stained with NKX6.1 and C-Peptide antibodies. DAPI is used to visualize the nuclei of all cells. Scalebar = 100µM. (F) Representative flow cytometry pseudo color dot plots of stage 6 cells derived from each of the three protocols. (G) Quantification of C-Peptide, NKX6.1 co-expressing cells as mean ± SEM, n = 6 independent experiments. For (D) and (G) Dots are color coded according to individual cell lines as indicated in legend of the figure. One-way ANOVA with Tukey test for multiple comparisons, $**P < 0.01$, $***P < 0.001$, different from the two other groups.

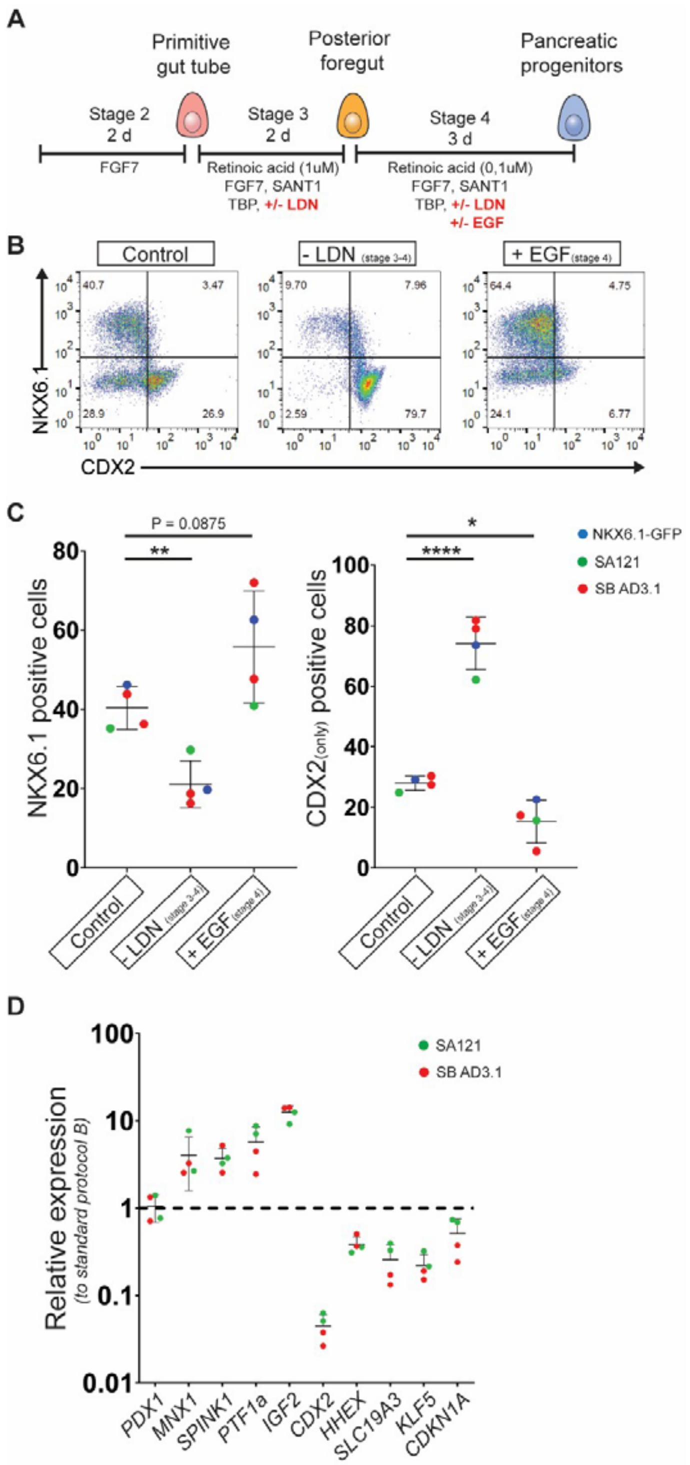
A
Primitive gut tube
Posterior foregut
Pancreatic progenitors
Stage 2
2 d
Stage 3
2 d
Stage 4
3 d
FGF7
Retinoic acid (1uM)
FGF7, SANT1
TBP, +/- LDN
Retinoic acid (0,1uM)
FGF7, SANT1
TBP, +/- LDN
+/- EGF
B
Control
- LDN (stage 3-4)
+ EGF (stage 4)
40.7
3.47
28.9
26.9
9.70
7.96
2.59
79.7
64.4
4.75
24.1
6.77
NKX6.1
CDX2
C
P = 0.0875
**
*
****
NKX6.1 positive cells
CDX2(only) positive cells
NKX6.1-GFP
SA121
SB AD3.1
Control
- LDN (stage 3-4)
+ EGF (stage 4)
D
SA121
SB AD3.1
Relative expression
(to standard protocol B)
PDX1
MNX1
SPINK1
PTF1a
IGF2
CDX2
HHEX
SLC19A3
KLF5
CDKN1A

Supplementary figure 5. Evaluation of BMP and EGF signalling in differentiation protocol A and B. Related to figure 5.

(A) Schematic outline of stage 2-4 of protocol A. Red text highlights compounds evaluated. (B) Representative examples of pseudo color dot plots of cells at the end of stage 4 differentiated with protocol A and modifications thereof. (C) Quantification of NKX6.1 positive (left) and $CDX2_{(only)}$ positive cells (right). Graphs shows percentage of positive cells as mean ± SEM, n = 4 independent experiments. Dots are color coded according to individual cell lines as indicated in legend of the figure. Unpaired t test applied to determine differences between individual groups. *P < 0.05, **P < 0.01, ****P < 0.0001. (D) Quantitative gene expression analysis of pancreatic progenitors derived using protocol B or a modified version of protocol B (Condition 3 in figure 5). Expression was normalized to the average expression of two housekeeping genes (*ACTB* and *HPRT1*). Figure shows expression of genes in the modified version of protocol B relative to the standard protocol B (dashed line). Mean ± SEM, n = 4.

RESOURCE TABLE
Reagents, antibodies, assays, cell lines and software applied.

REAGENT or RESOURCE	SOURCE	IDENTIFIER
Antibodies		
NKX6.1 Alexa 647 (FC 1:40)	BD Biosciences	Cat#563338; RRID: N/A
NKX6.1 PE (FC 1:40)	BD Biosciences	Cat#563023; RRID:AB_2716792
PDX1 Alexa 488 (FC 1:40)	BD Biosciences	Cat#562274; RRID:AB_10924596
NeuroD1 PE (FC 1:40)	BD Biosciences	Cat#563001; RRID:AB_2716791
NKX2.2 PE (FC 1:40)	BD Biosciences	Cat#564730; RRID: N/A
Ki-67 Alexa 488 (FC 1:20)	BD Biosciences	Cat#558616; RRID:AB_647087
Sox17 Alexa 488 (FC 1:40)	BD Biosciences	Cat#562205; RRID:AB_10893402
Oct3/4 Alexa 647 (FC 1:10)	BD Biosciences	Cat#560307; RRID:AB_1645319
CDX2 (FC 1:500)	ThermoFisher	Cat#RM-2116; RRID: N/A; clone EPR2764Y
Rabbit IgG (isotype control)	R&D systems	Cat#AB-105-C; RRID:AB_354266
PDX1 (IF 1:8000)	Abcam	Cat#AB47383; RRID:AB_2162359
NKX6.1 (IF 1:200)	DSHB	Cat#F55A12-S; RRID:AB_532379
C-Peptide/Pro-Insulin (IF 1:250)	DSHB	Cat#GN-ID4; RRID:AB_2255626
Donkey Anti-Rabbit Alexa488 (FC 1:500)	ThermoFisher	Cat#A21206; RRID:AB_2535792
Donkey Anti-Goat Alexa488 (IF 1:500)	ThermoFisher	
Donkey Anti-Mouse Alexa594 (IF 1:500)	ThermoFisher	
Donkey Anti-Rat Alexa488 (IF 1:500)	ThermoFisher	
DAPI solution (FC)	BD Biosciences	Cat#564907
LIVE/DEAD™ Fixable Violet Dead Cell Stain Kit	ThermoFisher	Cat#L34955
NucleoSpin RNA/Protein kit	Macherey-Nagel	Cat#740933.50
iScript cDNA Synthesis Kit	Bio-rad	Cat#170-8890
TagMan Gene Expression Master Mix	ThermoFisher Scientific	Cat##PN 4369016
ACTB TaqMan Assay	ThermoFisher Scientific	hs01060665_g1
HPRT1 TaqMan Assay	ThermoFisher Scientific	hs99999909_m1
PDX1 TaqMan Assay	ThermoFisher Scientific	hs00236830_m1
MNX1 TaqMan Assay	ThermoFisher Scientific	hs00907365_m1
PTF1A TaqMan Assay	ThermoFisher Scientific	hs00603586_g1
IGF2 TaqMan Assay	ThermoFisher Scientific	hs04188276_m1
CDX2 TagMan Assay	ThermoFisher Scientific	Hs01078080_m1
HHEX TaqMan Assay	ThermoFisher Scientific	hs00242160_m1
SPINK1 TaqMan Assay	ThermoFisher Scientific	hs00162154_m1
SLC19A3 TaqMan Assay	ThermoFisher Scientific	hs00228858_m1

KLF5 TaqMan Assay	ThermoFisher Scientific	hs00156145_m1
CDKN1A TaqMan Assay	ThermoFisher Scientific	hs00355782_m1
Bacterial and Virus Strains		
Biological Samples		
Chemicals, Peptides, and Recombinant Proteins		
hESC-qualified matrigel	BD biosciences	Cat#354277
Growth factor reduced matrigel	BD biosciences	Cat#356230
mTeSR1 medium	Stemcell technologies	Cat#05850
TeSR1-E8 medium	Stemcell technologies	Cat#05990
MCDB131 medium	ThermoFisher	Cat# 10372019
DMEM/F12 medium	ThermoFisher	Cat# 21331020
DMEM(HG) medium	ThermoFisher	Cat# 31966021
PBS-/- (without calcium and magnesium)	ThermoFisher	Cat#14190094
PBS+/+ (with calcium and magnesium)	ThermoFisher	Cat# 14040133
TrypLE select	ThermoFisher	Cat#12563011
4% Formaldehyde	VWR	Cat#9713.1000; CAS 50-00-0
Triton X-100	Sigma-Aldrich	Cat#T9284; CAS 9002-93-1
Donkey serum	Millipore	Cat#S30-100ML
$NaHCO_3$	ThermoFisher	Cat#25080060
Penicillin-Streptomycin solution	ThermoFisher	Cat#15140122
Glutamax	ThermoFisher	Cat#35050038
Glucose	Fisher scientific	Cat#D16500
BSA, fatty acid free	Proliant	Cat#68700
BSA Fraction V, fatty acid free	Sigma-Aldrich	Cat#10775835001
ITS-X	ThermoFisher	Cat#51500056
Zinc sulphate	Sigma-Aldrich	Cat# Z0251; CAS 7446-20-0
B-27 supplement (50x)	ThermoFisher	Cat#17504044
B-27 supplement (50x), minus vitamin A	ThermoFisher	Cat#12587010
Rock inhibitor	Sigma-Aldrich	Cat#Y0503; CAS 129830-38-2
Activin A	Peprotech	Cat#120-14
CHIR99021	Axon Medchem	Cat#1386; CAS 252917-06-9
L-Ascorbic acid	Sigma-Aldrich	Cat#A4544; CAS 50-81-7
Recombinant human KGF (FGF-7)	Peprotech	Cat#100-19
Retinoic acid	Sigma-Aldrich	Cat#R2625; CAS 302-79-4
SANT-1	Sigma-Aldrich	Cat#S4572; CAS 304909-07-7
LDN-193189	Stemgent	Cat#04-0074; CAS 1062368-24-4
TBP (PKC activator)	Merck Millipore	Cat#565740; CAS 497259-23-1
Heparin	Sigma-Aldrich	Cat#H3149; CAS 9041-08-1
3,3′,5-Triiodo-L-thyronine sodium salt (T3)	Sigma-Aldrich	CAT# T6397; CAS 55-06-1

ALK5 Inhibitor II	Enzo life sciences	Cat# ALX-270-445; CAS 446859-33-2
γ-Secretase Inhibitor XX	Merck Millipore	Cat#565789; CAS 209984-56-5
Recombinant Human EGF	R&D systems	Cat#236-EG
Recombinant Human Noggin	Peprotech	Cat#120-10C
Nicotinamide	Sigma-Aldrich	Cat#N0636; CAS 98-92-0
Critical Commercial Assays		
Deposited Data		
EGA RNA-seq		Study ID: EGAS00001003513 Data set: EGAS00001004823
EGA ATAC-seq		Study ID: EGAS00001003513 Data set: EGAS00001004824
Experimental Models: Cell Lines		
SB NEO1 Cl. 1	https://www.stembancc.org/ https://www.ebisc.org/	DOI: 10.1007/s00125-018-4612-4
SB AD2.1 hiPSC	https://www.stembancc.org/ https://www.ebisc.org/	DOI: 10.1007/s00125-018-4612-4 DOI: 10.1080/19382014.2016.1182276
SB AD3.1 hiPSC	https://www.stembancc.org/ https://www.ebisc.org/	DOI: 10.1007/s00125-018-4612-4
SB AD3.4 hiPSC	https://www.stembancc.org/ https://www.ebisc.org/	DOI: 10.1080/19382014.2016.1182276
SA121 hESC	Takara Bio	Cat# Y00020
NKX6.1-GFP hiPSC (clone 1a-21-3)	https://www.stembancc.org/ https://www.ebisc.org/	DOI: 10.1016/j.scr.2018.04.010
Experimental Models: Organisms/Strains		
Oligonucleotides		
Recombinant DNA		
Software and Algorithms		
FASTQC		https://www.bioinformatics.babraham.ac.uk/projects/fastqc/

STAR (v.2.5.1)	DOI: 10.1093/bioinformatics/bts635	https://github.com/alexdobin/STAR
Picard tools suite (v2.1.1) - MarkDuplicates		https://broadinstitute.github.io/picard/
featureCounts	DOI: 10.1093/bioinformatics/btt656	http://bioinf.wehi.edu.au/featureCounts/
Kundaje lab ATAC-seq pipeline		https://github.com/kundajelab/atac-seq-pipeline
Bowtie2 (v.2.2.6)	DOI: 10.1038/nmeth.1923.	http://bowtie-bio.sourceforge.net/bowtie2/index.shtml
Bedtools (v.2.26)	DOI:10.1093/bioinformatics/btq033	https://bedtools.readthedocs.io/en/latest/
MACSv2 (2.1.0)	DOI: 10.1186/gb-2008-9-9-r137	https://github.com/taoliu/MACS
DESeq2 (v.1.32.0)	DOI: 10.1186/s13059-014-0550-8	https://www.bioconductor.org/packages/release/bioc/html/DESeq2.html
Sva (v.3.28.0)	DOI: 10.1093/bioinformatics/bts034	http://bioconductor.org/packages/release/bioc/html/sva.html
edgeR (v.3.22.2)	DOI: 10.1093/bioinformatics/btp616	http://bioconductor.org/packages/release/bioc/html/edgeR.html
RUVseq (v.1.14.0)	DOI: 10.1038/nbt.2931	https://bioconductor.org/packages/release/bioc/html/RUVSeq.html
DAVID	DOI: 10.1186/gb-2007-8-9-r183	https://david.ncifcrf.gov/home.jsp
HOMER	DOI: 10.1016/j.molcel.2010.05.004	http://homer.ucsd.edu/homer/
msCentipede	DOI: 10.1371/journal.pone.0138030	https://github.com/rajanil/msCentipede
JASPAR Core	DOI: 10.1093/nar/gkx1126	http://jaspar.genereg.net/collection/core/
FIMO	DOI: 10.1093/bioinformatics/btr064	http://meme-suite.org/tools/fimo
atactk toolkit (v.0.1.6)		https://github.com/ParkerLab/atactk

ggplot2	https://cran.r-project.org/web/packages/ggplot2/citation.html	https://cran.r-project.org/web/packages/ggplot2/index.html
WGCNA (v.1.64.1)	DOI: 10.1186/1471-2105-9-559	https://cran.r-project.org/web/packages/WGCNA/index.html
AmiGO 2	DOI: 10.1093/bioinformatics/btn615	http://amigo.geneontology.org/amigo
Other		

SUPPLEMENTAL EXPERIMENTAL PROCEDURES

Maintenance and differentiation of hPSC lines

The following hPSC lines were applied in this study: SA121 hESC, SB NEO1.1 hiPSC, SB AD2.1 hiPSC, SB AD3.1 hiPSC, SB AD3.4 hiPSC and NKX6.1-GFP hiPSC line. Details on source of lines can be found in the resource table in the supplemental information. All hPSC were maintained on hESC-qualified matrigel in mTeSR1 with the exception of the NKX6.1-GFP hiPSC line, which was cultured in TeSR1-E8 medium at 37°C, 5% CO^2 as previously described (Perez-Alcantara et al., 2018). Briefly, cells were passaged every 3-4 days or when reaching 90-95% confluence. Medium was aspirated and the cells were washed once with PBS without calcium and magnesium (PBS-/-) and subsequently incubated with TrypLE select (3-5min, 37°C) to achieve a single cell solution. TrypLe select wash quenched with 3-4 volumes of medium with 5µM Rock inhibitor and cells were pelleted (300xG, 5min). Cell number was determined using a NucleoCounter NC-200 (Chemotec, Denmark) and cells were resuspended to the desired concentration in medium with 5µM Rock inhibitor. Cells were seeded in medium with 5uM Rock inhibitor onto tissue culture flasks (Nunclon delta surfaces, Thermo Scientific, #156367 or #156499) pre-coated with hESC-qualified matrigel for maintenance or onto 12-well tissue culture plates (CellBIND surfaces, Corning #3336) for differentiation. For differentiation, cells were seeded at 300.000-400.000 cells/cm^2 and 24h after seeding the cells, medium was aspirated and cells were washed once with PBS with calcium and magnesium (PBS+/+) before exposing the cells to differentiation medium. Stage 1 (definitive endoderm) was shared across all three protocols. Cells were incubated for 1 day with MCDB131 medium containing 0.5% BSA (w/v), 0.1% (v/v) Penicillin-Streptomycin, 1% (v/v) Glutamax, 10mM glucose, 100ng/ml Activin A and 3uM CHIR99021. For day 2, cells were cultured with MCDB131 medium containing 0.5% BSA (w/v), 0.1% (v/v) Penicillin-Streptomycin, 1% (v/v) Glutamax, 10mM glucose, 100ng/ml Activin A and 0.3uM CHIR99021. For day 3 cells were cultured with MCDB131 medium containing 0.5% BSA (w/v), 1.5 g/l $NaHCO_3$, 0.1% (v/v) Penicillin-Streptomycin (PS), 1% (v/v) Glutamax, 10mM glucose and 100ng/ml Activin A. Following induction of definitive endoderm, cells were differentiated with three pancreatic progenitor protocols outlined below. Importantly, differentiations with the three protocols were always performed side-by-side. Medium was prepared freshly for each stage of the differentiation protocol and cells were replenished with medium daily across all protocols and stages. Small molecules and growth factors were aliquoted to appropriate volumes to reduce the number of freeze-thaw cycles (≤3).

**PROTOCOL A adapted from (Rezania et al., 2014)**			
**Stage**	**Medium**	**Compounds**	**Key differences compared to original protocol**
**Stage 2** (2 days)	MCDB131, 0.1% PS, 1% glutamax, 1.5g/l $NaHCO_3$ 10mM glucose, 0.5% BSA, 0.25mM ascorbic acid	50ng/ml KGF (FGF7)	• Differences in maintenance of undifferentiated hPSC compared to original study

**Stage 3** (2 days)	MCDB131, 0.1% PS, 1% glutamax, 2.5g/l $NaHCO_3$ 10mM glucose, 2% BSA, 0.25mM ascorbic acid , 1:200 (v/v) ITS-X	50ng/ml KGF (FGF7), 1µM Retinoic acid, 0.25µM SANT-1, 100nM LDN-193189, 200nM TBP	• CHIR99021 and Activin A used for DE induction compared to MCX-928 and GDF8 in the original publication.<br>• MCDB131 medium used for stage 3-4 compared to custom made medium (BLAR) reported in the original publication.<br>• Differences in vendors used for various supplements and factors.
**Stage 4** (3 days)		2ng/ml KGF (FGF7), 0.1µM Retinoic acid, 0.25µM SANT-1, 200nM LDN-193189, 100nM TBP	

**PROTOCOL B adapted from (Russ et al., 2015)**			
**Stage**	**Medium**	**Compounds**	**Key differences compared to original protocol**
**Stage 2** (2 days)	MCDB131, 0.1% PS, 1% glutamax, 1.5g/l $NaHCO_3$ 10mM glucose, 0.5% BSA, 0.25mM ascorbic acid	50ng/ml KGF (FGF7)	• Differences in maintenance of undifferentiated hPSC compared to original study<br>• Cells differentiated in 2D format compared to suspension culture in the original publication.<br>• Different protocol for DE induction compared to original publication<br>• Differences in medium and FGF7 concentration in stage 2.<br>• Different retinoic acid receptor agonist applied<br>• Differences in vendors used for various supplements and factors.<br>• Differences in length of stage 4.2 compared to original publication
**Stage 3** (2 days)	DMEM(HG) 0.1% PS, 1% B-27 supplement	2µM Retinoic acid	
**Stage 4.1** (1 day)		2µM Retinoic acid, 50ng/ml EGF	
**Stage 4.2** (2 days)		50ng/ml KGF (FGF7), 50ng/ml EGF	

**PROTOCOL C adapted from (Nostro et al., 2015)**			
**Stage**	**Medium**	**Compounds**	**Key differences compared to original protocol**

**Stage 2**<br>(3 days)	DMEM/F12, 0.1% PS, 1% Glutamax, 1% N2 supplement, 1% B27 supplement (- Vitamin A), 0.05% BSA, 0.5mM Ascorbic acid	50ng/ml KGF (FGF7), 50ng/ml Noggin*<br>(*cell line dependent*)	• Differences in maintenance of undifferentiated hPSC compared to original study<br>• Different protocol for DE induction compared to original publication<br>• No Wnt3a in stage 2 of protocol<br>• FGF7 used instead of FGF10 throughout<br>• SANT-1 used compared to KAAD-Cyclopamine in original publication<br>• Differences in vendors used for various supplements and factors.<br>• Activin A during stage 4 of protocol.
**Stage 3**<br>(1 day)	DMEM(HG) 0.1% PS, 1% B-27 supplement (- Vitamin A), 0.5mM Ascorbic acid	2µM Retinoic acid, 50ng/ml KGF (FGF7), 50ng/ml Noggin	
**Stage 4**<br>(3 days)		100ng/ml EGF, 50ng/ml Noggin, 10mM Nicotinamide, 50ng/ml Activin A	

*Omission of Noggin from stage 2 of protocol C resulted in a significantly more efficient differentiation of the SB AD3.1 hiPSC line. The NKX6.1-GFP reporter hiPSC line was derived from the SB AD3.1 hiPSC line and thus, all differentiations of the NKX 6.1-GFP reporter hiPSC line with protocol C was carried out without Noggin during stage 2.

For subsequent endocrine differentiation of the pancreatic progenitors derived from all three protocols, stage 5 and stage 6 of the differentiation protocol outlined below was applied.

**ENDOCRINE DIFFERENTIATION adapted from (Rezania et al., 2014)**		
**Stage**	**Medium**	**Compounds**
**Stage 5**<br>(3 days)	MCDB131, 0.1% PS, 1% glutamax, 1.5g/l $NaHCO_3$ 20mM glucose, 2% BSA, 0.5% ITS-X, 10uM Zinc sulfate	0.05µM Retinoic acid, 0.25µM SANT-1, 100nM LDN-193189, 10µM ALK5i II, 1µM T3, 10µg/ml heparin
**Stage6**<br>(7 days)		100nM LDN-193189, 10µM ALK5i II, 1µM T3, 10µg/ml heparin, 100nM γ-secretase inhibitor XX

Immunofluorescence imaging

Cells were washed once in PBS and fixed directly in the wells of tissue culture plates by incubating with 4% formaldehyde for 20min at room temperature. The fixed cells were subsequently rinsed twice in PBS and then permeabilized for 10min at room temperature in PBS containing 0.5% Triton X-100. Following permeabilization, cells were incubated for 30 min at room temperature with a blocking solution consisting of 0.1 M Tris-HCl pH 7.4, 0.15 M NaCl, and 0.5% Tyramide Signal Amplification (TSA) immunohistochemistry kit blocking reagent. Cells were subsequently incubated with primary antibodies diluted in PBS with 0.1% Triton X-100 overnight at 4°C. Following incubation with primary antibodies, cells were washed three times in PBS and subsequently incubated with secondary antibodies and 4',6-diamidino-2-phenylindole in PBS with 0.1% Triton X-100 for 45min at room temperature. Images were acquired with an inverted fluorescence microscope (Olympus IX-81).

Flow cytometry analysis

Cells were harvested to a single cell suspension using TrypLE select, rinsed once in PBS and stained with Live/Dead cell kit for 10min on ice. Cells were subsequently washed and fixated for 20min at 4°C in 4% formaldehyde. Cells were washed in PBS and incubated for 30min in perm-buffer (PBS +/+ with 0.2% Triton X-100 and 5% Donkey serum) at 4°C. Following permeabilization, cells were pelleted (800 x G, 5min) and resuspended in block-buffer (PBS +/+ with 0.1% Triton X-100 and 5% Donkey serum) containing antibodies. Cells were incubated for 30min at room temperature covered from light and subsequently washed twice in PBS with 1% BSA. Cells were analysed on BD LSR Fortessa cell analyser (BD Biosciences) with 10.000-20.000 events recorded pr. sample.

Fluorescence activated cell sorting
An NKX6.1-T2A-GFP reporter iPSC lines was differentiated side-by-side to the pancreatic progenitor stage using the three differentiation protocols. A wild type iPSC line was also differentiated and used to define the gates for sorting the GFP- and GFP+ fractions. A total of three independent differentiations were performed and from each differentiation both GFP+ and GFP- cells as well as unsorted controls were FACS purified. Briefly, cells were harvested to a single cell suspension using TrypLE select, pelleted and resuspended in the stage 4 medium of the respective protocols (without factors) containing 1µg/ml DAPI solution and directly proceeded for sorting. Cells were sorted using a BD FACSAria Fusion (BD Biosciences) with the 100µM nozzle/20psi configuration. GFP+ and GFP- cells were sorted into Eppendorf tubes containing medium at 4°C. Between 50.000-100.000 cells were sorted for RNA sequencing and 45.000-60.000 cells were sorted for ATAC sequencing.
Real time quantitative PCR (RT-qPCR). RNA was isolated from harvested cells using NucleoSpin RNA/Protein kit. 500ng RNA was converted to cDNA using iScript cDNA Synthesis Kit and adjusted to a final volume of 200ul following reverse transcription. Gene expression was analysed using TagMan Gene Expression Assays (see resource table below). RT-qPCR reactions consisted of 0,5ul Tagman Gene expression assay, 2ul cDNA, 5ul TagMan Gene Expression Master Mix and 2,5ul H_2O. RT-qPCR was performed on a MX3005P qPCR system (Agilent Genomics) using a fast-2-step protocol (first 95°C for 1min, then 40 cycles of 95°C for 10sec, 60°C for 25sec). Expression values were normalized to the average expression of two housekeeping genes (ACTB and HPRT1) and relative expression to unsorted samples was calculated using the ΔΔCt method.

RNA sequencing and processing of sequence data
For RNA sequencing, cells were pelleted immediately following sorting and medium was removed. Cells were harvested and RNA extracted using TRIzol Reagent (ThermoFisher Scientific, Paisley, UK) as per the manufacturer's guidelines. Library preparation and sequencing was performed at the Oxford Genomics Centre (Wellcome Centre for Human Genetics, Oxford, UK) as previously described(van de Bunt et al., 2016). We prepared the Smart-Seq2 paired-end RNA-seq libraries for the 27 samples (3 differentiations x 3 protocols x 3 cell populations: presort, GFP+ and GFP-), and sequenced on Illumina HiSeq4000 to a mean depth of X (+/- X) million 75bp reads pairs per sample. The sequencing quality was assessed with FASTQC (http://www.bioinformatics.babraham.ac.uk/projects/fastqc/) and the raw sequencing reads were mapped to the human genome hg19 using STAR version 2.5.1 with default settings. The GENCODE v19 GTF was applied to guide the spliced alignment. Duplicated alignments were marked with the MarkDuplicates script from the Picard tools v2.1.1 suite (http://broadinstitute.github.io/picard). Gene expression was quantified with featureCounts using the GENCODE v19 GTF file. We assessed the library complexities for all the samples by comparing the number of genes detected at >1TPM with the percentage of total reads mapping within the top 100 expressed genes. We discovered that one of the presort samples from protocol A from differentiation 3 had much lower library complexity (X) than the remaining samples (X), therefore we removed it from further analyses.

Transposition reaction and Purification/ATAC-seq
ATAC-seq sample preparation was done as described previously(Buenrostro et al., 2013). Described shortly, following sorting cells were pelleted and washed in cold PBS. Cell pellets were gently resuspended in cold lysis buffer (10mM Tris-HCL, pH 7.4, 10mM NaCl, 3mM MgCl2, 0.1% IGEPAL CA-630) and immediately pelleted by centrifugation. The supernatant was discarded and the cell pellets were gently resuspended in 50µl transposition reaction mix (25µl 2 x TD buffer, 2.5µl Tn5 Transposase (Illumina), 22.5µl Nuclease free H_2O) and incubated at 37°C for 30min. Following the transposition reaction, the transposased DNA was purified using Qiagens MinElute Kit (Qiagen) according to manufacturer's instructions and eluted in 10µl elution buffer (10mM Tris buffer, pH 8). Eluted DNA was PCR amplified using Nextera PCR primers (Illumina) and NEBNext high fidelity PCR master mix (New England Biolabs) with the following cycling 1) 72°C, 5 min, 2) 98°C, 30 sec , 3) 98°C, 10 sec, 4) 63°C, 30 sec, 5) 72°C, 1 min, Repeating steps 3-5, 9-11x. PCR product was purified using Qiagens MinElute Kit (Qiagen) according to manufacturer's instructions and eluted in 20µl elution buffer (10mM Tris buffer, pH 8). Quality of final

library was assessed using Tapestation (agilent). Libraries were sequenced at the High-Throughput Genomics core at the Wellcome Centre for Human Genetics, University of Oxford, UK. Samples were sequenced as multiplexed pools on HiSeq4000 (Illumina) using 75-bp paired-end sequencing.
The ATAC-seq libraries for the 27 samples (3 differentiations x 3 protocols x 3 cell populations: presort, GFP+ and GFP-) were sequenced on Illumina HiSeq4000 to a mean depth of X (+/- X) million 75bp reads pairs per sample. The raw sequencing reads were processed with the Kundaje lab ATAC-seq pipeline (https://github.com/kundajelab/atac-seq-pipeline). The pipeline detects and trims the sequencing adaptors, maps the reads to the human reference genome build hg19 using Bowtie2 version 2.2.6. The duplicated alignments are marked with MarkDuplicates script from the Picard tolos suite, converts the BAM files to tagAlign format using bedtools versión 2.26 and calls the signal peaks using MACSv2 (2.1.0).

Principal component analysis
The global similarities between the samples for both the RNA-seq and ATAC-seq datasets were assessed through principal component analysis (PCA). The gene count tables were transformed with variance stabilizing transformation (vst) in the DESeq2 R package (v.1.32.0)(Love et al., 2014). The batch effects of the two multiplexing pools introduced for sequencing in the ATAC-seq dataset were corrected using the sva R package (v.3.28.0)(Leek et al., 2012). The PCA analysis was performed using the plotMDS function from the edgeR R package (v.3.22.2)(Robinson et al., 2010), using the setting gene.selection="common", and focusing on the top 500 most variable genes. For the PCA analysis of RNA-seq samples from this study together with the RNA-seq dataset from a study of human fetal pancreas populations (Ramond 2018), presented in Fig.3D, we merged the raw gene count tables for the two studies, performed the variance stabilizing transformation in DESeq2 on the merged count table, followed by sva batch correction for the effect of the study of origin.

Defining the common PP signature
To derive common transcriptomic and epigenomic signatures of pancreatic progenitors generated in this study with three differentiation protocols, we compared the data to the equivalent omics profiles of all stages of hPSC differentiation towards beta-line cells (Perez Alcantara 2018). To facilitate the comparison we applied the RUVs normalization implemented in the RUVseq (v.1.14.0) R package(Risso et al., 2014). We first applied the upper-quartile normalization to account for differences in library sizes, followed by the RUVs approach to estimate and remove the factors of unwanted variation. For that purpose we treated the presorted samples generated with protocol A as replicates of the control lines samples from the pancreatic endoderm stage (also generated with protocol A). After the RUVs normalization, we generated the normalized count tables for all the samples and used them to calculate CPM (counts-per-million) expression values using the cpm() function from the edgeR R package. We then calculated mean CPM values for each sample group (GFP cell population from each protocol, and all differentiation stages). We then defined pancreatic progenitor signatures within the GFP+ cell populations for each protocol separately; and then we used the intersection of the three individual protocol-specific signatures to define the common cross-protocol pancreatic progenitor signature. To define the sets of genes forming a pancreatic progenitor signature for each protocol, we required that the genes were expressed at >1 TPM in the GFP+ population from the given protocol, demonstrated stage selectivity (CV>1), as well as specificity to the pancreatic endoderm (Z-score >1) when evaluated together with the remaining stages. We calculated similar PP signatures in the presort, and GFP-populations as well. The PP signatures were derived in the same manner for both the RNA-seq and ATAC-seq datasets. Functional enrichments for the PP signature genes were calculated with DAVID Gene Functional Classification tool(Huang et al., 2007); raw enrichment p-values for selected Gene Ontology categories were then plotted as -log10(p-value) barplots in Fig.3C. Enrichment of known transcription factor binding motifs was calculated with HOMER(Heinz et al., 2010) using the default settings.

Footprinting analysis
msCentipede(Raj et al., 2015) was used to call sites bound by transcription factors. First, transcription factor binding motifs were called genome-wide using position weight matrices from JASPAR Core (non-redundant) 2018 (Khan et al., 2018) using FIMO (version 4.11.2, (Grant et al., 2011)) with default parameters. The model parameters were learned per experiment and GFP status using genome-wide called motifs and replicate bam files. Transcription factor binding motifs were called in each replicate peak file (for high quality peaks with q-value < 10^{-6}; per experiment and GFP status), and subsequently the replicates were merged and used as input for the inference step in msCentipede. Sites with the following scores were considered bound: LogPosOdds > 2, MultLikeRatio>1 and NegBinLikeRatio>1; and the mean counts per site were calculated using make_cut_site function in the atactk toolkit (v. 0.1.6)(https://github.com/ParkerLab/atactk) and plotted using ggplot2 (Wickham, 2016) in R (v. 3.4.3).

DEGs/DOCS

Testing for differential expressed genes (DEGs) and differentially open chromatin sites (DOCS) was performed with DESeq2 R package (v…). We compared data from each differentiation protocol to the data generated with the two other protocols, for each cell population (presort, GFP+ and GFP-) separately. We also compared the data between the GFP+ and GFP- cell populations for each protocol separately. In both RNA-seq and ATAC-seq analyses we adjusted for the effects of the differentiations, and in the ATAC-seq additionally also for the effects of the sequencing multiplex pool. We considered all genes and open chromatic sites with FDR-adjusted p-values<0.05 as
significantly differentially expressed.

WGCNA

We performed weighted gene co-expression analysis (WGCNA)(Langfelder and Horvath, 2008) on both the vst-transformed RNA-seq and ATAC-seq datasets. The RNA-seq analysis was performed on 15,555 protein-coding genes expressed at >=1 TPM in at least 3 samples. The ATAC-seq analysis was limited to 19,739 open chromatin peaks, with >=10 sequencing reads in at least 3 samples, and a coefficient of variation >0.5. Using the criteria of best fit to the scale free topology model we selected power of 6 for soft thresholding of RNA-seq data, and power of 3 for the ATAC-seq dataset, and required a minimum module size of 50 for both analyses. We identified 20 co-expressed gene modules in the RNA-seq dataset, and 11 modules of correlated open chromatin sites. The dimensionality reduction of these large datasets is achieved through assignment of a single eigengene value for each modules in each sample. The eigengene value represents the first principal component value for the given module genes or open chromatin regions. The module eigengene values for the samples with both RNA-seq and ATAC-seq data can then be correlated to derive pairs of highly correlated modules. These are likely to correspond to open chromatin at regulatory elements controlling the gene expression of the correlated genes.

Gene signatures and Gene Ontology functional enrichment testing

For each of the 20 co-expressed gene modules we tested enrichment in selected gene sets using the hypergeometric distribution. We investigated enrichments of the *in vitro* pancreatic progenitor signatures derived in this study, as
described in an earlier section, as well as the 500 gene PP signature derived through comparison of *in vitro* and *in vivo* multipotent pancreatic progenitors from a previous study by Cebola *et al*. 2015. We then tested whether any of the gene modules were enriched in selected developmental Gene Ontology terms: GO:0031016 pancreas development, GO:0001889 liver development , GO:0048565 digestive tract development and GO:0060575 intestinal
epithelial cell differentiation. Genes belonging to these Gene Ontology categories were identified using AmiGO 2 Ontology search function(Carbon et al., 2009), accessed on 10th April 2018. We tested whether any of the gene modules might represent cells with likely intestinal fate through enrichment of previously reported genes differentially and highly expressed in intestinal stem cells derived from duodenum, jejunum and ileum(Wang et al., 2015). We also performed differential gene expression to identify significantly up-regulated biomarkers of dorsal pancreas, hepatobiliary primordium and hepatic cords from laser capture dissection in human embryos(Jennings et
al., 2017). We then tested enrichment for these sets of markers with log2FC>0 and FDR q-value<0.05 in the co-expressed gene modules using the hypergeometric distribution. Enrichment of known transcription factor binding motifs within co-open chromatin modules was calculated with HOMER using the default settings(Heinz et al., 2010).

SUPPLEMENTAL REFERENCES

Buenrostro, J. D., Giresi, P. G., Zaba, L. C., Chang, H. Y. & Greenleaf, W. J. (2013). Transposition of native chromatin for fast and sensitive epigenomic profiling of open chromatin, DNA-binding proteins and nucleosome position. Nat Methods, 10, 1213-8.

Carbon, S., Ireland, A., Mungall, C. J., Shu, S., Marshall, B., Lewis, S., Ami, G. O. H. & Web Presence Working, G. (2009). AmiGO: online access to ontology and annotation data. Bioinformatics, 25, 288-9.

Grant, C. E., Bailey, T. L. & Noble, W. S. (2011). FIMO: scanning for occurrences of a given motif. Bioinformatics, 27, 1017-8.

Heinz, S., Benner, C., Spann, N., Bertolino, E., Lin, Y. C., Laslo, P., Cheng, J. X., Murre, C., Singh, H. & Glass, C. K. (2010). Simple combinations of lineage-determining transcription factors prime cis-regulatory elements required for macrophage and B cell identities. Mol Cell, 38, 576-89.

Huang, D. W., Sherman, B. T., Tan, Q., Collins, J. R., Alvord, W. G., Roayaei, J., Stephens, R., Baseler, M.

W., Lane, H. C. & Lempicki, R. A. (2007). The DAVID Gene Functional Classification Tool: a novel biological module-centric algorithm to functionally analyze large gene lists. Genome Biol, 8, R183.
Jennings, R. E., Berry, A. A., Gerrard, D. T., Wearne, S. J., Strutt, J., Withey, S., Chhatriwala, M., Piper Hanley, K., Vallier, L., Bobola, N., et al. (2017). Laser Capture and Deep Sequencing Reveals the Transcriptomic Programmes Regulating the Onset of Pancreas and Liver Differentiation in Human Embryos. Stem Cell Reports, 9, 1387-1394.
Khan, A., Fornes, O., Stigliani, A., Gheorghe, M., Castro-Mondragon, J. A., van der Lee, R., Bessy, A., Cheneby, J., Kulkarni, S. R., Tan, G., et al. (2018). JASPAR 2018: update of the open-access database of transcription factor binding profiles and its web framework. Nucleic Acids Res, 46, D260-D266.
Langfelder, P. & Horvath, S. (2008). WGCNA: an R package for weighted correlation network analysis. BMC Bioinformatics, 9, 559.
Leek, J. T., Johnson, W. E., Parker, H. S., Jaffe, A. E. & Storey, J. D. (2012). The sva package for removing batch effects and other unwanted variation in high-throughput experiments. Bioinformatics, 28, 882-3.
Love, M. I., Huber, W. & Anders, S. (2014). Moderated estimation of fold change and dispersion for RNA-seq data with DESeq2. Genome Biol, 15, 550.
Nostro, M. C., Sarangi, F., Yang, C., Holland, A., Elefanty, A. G., Stanley, E. G., Greiner, D. L. & Keller, G. (2015). Efficient generation of NKX6-1+ pancreatic progenitors from multiple human pluripotent stem cell lines. Stem Cell Reports, 4, 591-604.
Perez-Alcantara, M., Honore, C., Wesolowska-Andersen, A., Gloyn, A. L., McCarthy, M. I., Hansson, M., Beer, N. L. & van de Bunt, M. (2018). Patterns of differential gene expression in a cellular model of human islet development, and relationship to type 2 diabetes predisposition. Diabetologia, 61, 1614-1622.
Raj, A., Shim, H., Gilad, Y., Pritchard, J. K. & Stephens, M. (2015). msCentipede: Modeling Heterogeneity across Genomic Sites and Replicates Improves Accuracy in the Inference of Transcription Factor Binding. PLoS One, 10, e0138030.
Rezania, A., Bruin, J. E., Arora, P., Rubin, A., Batushansky, I., Asadi, A., O'Dwyer, S., Quiskamp, N., Mojibian, M., Albrecht, T., et al. (2014). Reversal of diabetes with insulin-producing cells derived in vitro from human pluripotent stem cells. Nat. Biotechnol, 32, 1121-1133.
Risso, D., Ngai, J., Speed, T. P. & Dudoit, S. (2014). Normalization of RNA-seq data using factor analysis of control genes or samples. Nat Biotechnol, 32, 896-902.
Robinson, M. D., McCarthy, D. J. & Smyth, G. K. (2010). edgeR: a Bioconductor package for differential expression analysis of digital gene expression data. Bioinformatics, 26, 139-40.
Russ, H. A., Parent, A. V., Ringler, J. J., Hennings, T. G., Nair, G. G., Shveygert, M., Guo, T., Puri, S., Haataja, L., Cirulli, V., et al. (2015). Controlled induction of human pancreatic progenitors produces functional beta-like cells in vitro. EMBO J, 34, 1759-72.
van de Bunt, M., Lako, M., Barrett, A., Gloyn, A. L., Hansson, M., McCarthy, M. I., Beer, N. L. & Honore, C. (2016). Insights into islet development and biology through characterization of a human iPSC-derived endocrine pancreas model. Islets, 8, 83-95.
Wang, X., Yamamoto, Y., Wilson, L. H., Zhang, T., Howitt, B. E., Farrow, M. A., Kern, F., Ning, G., Hong, Y., Khor, C. C., et al. (2015). Cloning and variation of ground state intestinal stem cells. Nature, 522, 173-8.
Wickham, H. (2016). ggplot2: Elegant Graphics for Data Analysis. Springer-Verlag New York.
